# Pembrolizumab in Patients With Advanced Clear Cell Gynecological Cancer: A Phase 2 Nonrandomized Clinical Trial

**DOI:** 10.1001/jamaoncol.2024.6797

**Published:** 2025-02-06

**Authors:** Rebecca Kristeleit, Michael-John Devlin, Andrew Clamp, Charlie Gourley, René Roux, Marcia Hall, Rachel Nirsimloo, Valentinos Kounnis, Lesley Sage, Priya Narayanan, C. Simon Herrington, Rupali Arora, Laura Farrelly, Laura Hughes, Nicholas Counsell, Rowan E. Miller

**Affiliations:** 1 Guy’s and St Thomas’ NHS Foundation Trust and Comprehensive Cancer Centre, King’s College London, London, United Kingdom; 2 Queen Mary University of London, London, United Kingdom; 3 The Christie NHS Foundation Trust and University of Manchester, Manchester, United Kingdom; 4 Edinburgh Cancer Centre, Western General Hospital, Edinburgh, United Kingdom; 5 Nicola Murray Centre for Ovarian Cancer Research, Cancer Research United Kingdom Scotland Centre, Institute of Genetics and Cancer, University of Edinburgh, Edinburgh, United Kingdom; 6 Oxford University Hospitals NHS Foundation Trust, Churchill Hospital, Oxford, United Kingdom; 7 Hillingdon Hospitals NHS Foundation Trust, Mount Vernon Hospital, Middlesex, United Kingdom; 8 Gynae-Oncology Trials Group United Kingdom and Ovacome, London, United Kingdom; 9 University College London Hospitals, London, United Kingdom; 10 Cancer Research United Kingdom & UCL Cancer Trials Centre, University College London, London, United Kingdom

## Abstract

**Importance:**

Advanced clear cell gynecological cancers (CCGCs) have a poor prognosis, with response rates to second-line chemotherapy less than 8%. Preliminary clinical activity with programmed cell death 1 protein (PD-1) inhibitors reported in CCGC merits further investigation.

**Objective:**

To assess the clinical benefit of pembrolizumab in patients with previously treated advanced CCGC.

**Design, Setting, and Participants:**

The PEACOCC trial is a single-arm multicenter phase 2 trial conducted at 5 UK centers investigating the clinical benefit and safety of pembrolizumab. PD-1 inhibitor–naive patients with histologically confirmed advanced CCGC, radiological disease progression following 1 or more prior courses of chemotherapy, and an Eastern Cooperative Oncology Group (ECOG) performance status (PS) score of 0 to 1 were included. Patients were enrolled from March 2019 to October 2021, with data collected until July 2024.

**Interventions:**

Pembrolizumab, 200 mg, intravenously every 21 days up to 2 years until progression, discontinuation due to toxic effects, or patient/clinician decision. Up to 1 year of retreatment on diseases progression, if stable disease, partial response, or complete response at 2 years.

**Main Outcomes and Measures:**

The primary end point was progression-free survival (PFS) rate at 12 weeks using Response Evaluation Criteria in Solid Tumors version 1.1 to detect a 12-week PFS rate of 33% or greater and exclude a PFS rate of less than 15%, with 90% power and 1-sided 5% significance level. Secondary end points included objective response rate, duration of response, PFS, overall survival, safety, and quality of life.

**Results:**

A total of 48 patients were eligible. The median (range) age was 58.5 (32-77) years, and 26 (54%) had an ECOG PS score of 0 and 22 (46%) had an ECOG PS score of 1; 41 (85%) had ovarian, 6 (13%) had endometrial, and 1 (2%) had cervical advanced CCGC. The median (range) courses prior therapy was 3 (1-6); 19 patients (40%) received prior anti-angiogenic therapy, and 19 (40%) had a platinum-free interval of more than 12 months. Grade 3 treatment-related adverse events were observed in 9 patients (19%), and no patients had grade 4 or 5 adverse events. A total of 45 of 46 patients (98%) had mismatch repair–proficient tumors. The 12-week PFS rate was 42% (95% CI, 28-57), and the best objective response rate was 25% (95% CI, 14-40), with 12 partial responses. After a median follow-up of 46.9 months (95% CI, 43.4-55.0), the median PFS was 2.7 months (95% CI, 1.3-5.4), and the median overall survival was 14.8 months (95% CI, 6.7-28.2).

**Conclusions and Relevance:**

The PEACOCC trial showed clinical benefit with pembrolizumab in patients with previously treated advanced CCGC, of whom all except 1 had MMR-proficient disease. Clinical outcomes were durable with an overall tolerable safety profile, justifying further evaluation of pembrolizumab monotherapy for advanced CCGC in a randomized clinical trial.

**Trial Registration:**

ClinicalTrials.gov Identifier: NCT03425565

## Key Points

**Question:**

Does the programmed cell death 1 protein inhibitor pembrolizumab demonstrate clinical benefit as monotherapy in patients with advanced clear cell gynecological cancer (CCGC) previously treated with chemotherapy?

**Findings:**

This phase 2 nonrandomized clinical trial including 48 patients met the primary end point with a 12-week progression-free survival rate of 42%, with the confidence interval exceeding the prestated lower bound of 15%, indicating pembrolizumab is effective in patients with previously treated advanced CCGC. The overall response rate was 25%, the median duration of response 13.1 months, the median overall survival was 14.8 months, and the safety profile was overall tolerable.

**Meaning:**

Pembrolizumab showed clinical benefit in patients with previously treated advanced CCGC, warranting further evaluation in a randomized clinical trial.

## Teaser

This phase nonrandomized clinical trial assesses the clinical benefit of pembrolizumab in patients with previously treated advanced clear cell gynecological cancer.

## Introduction

Clear cell gynecological cancers (CCGCs) represent a subgroup of malignant tumors characterized by distinct clinicopathological features, including an association with endometriosis, which is present pathologically in 50% of cases. Patients with clear cell cancer tend to be younger, present more commonly with early-stage disease, and have a higher risk of arterial venous thrombosis and hypercalcaemia.^1,2^ While clear cell ovarian cancers (CCOC; including fallopian tube and primary peritoneal cancers) are most common, representing up to 15% of all epithelial ovarian cancers (EOC), 5% to 8% of clear cell tumors of the endometrium (CCEC), and less than 5% of CCGCs of the vagina, vulva, and cervix.^1,2,3,4,5^ Advanced CCOC studies consistently demonstrate significantly poorer prognosis than other subtypes,^5,6,7^ with response rates to first-line carboplatin-paclitaxel chemotherapy of 25%^8^ and to second-line chemotherapy of less than 8%.^9,10^ CCEC represents a difficult-to-treat subgroup, which are relatively resistant to radiotherapy or chemotherapy and have poorer survival than other.^1,4^ Consequently, CCGC represent a disease subtype where there is significant unmet need, and efforts to identify effective therapies are required.

These anatomically distinct tumors have overlapping gene expression profiles and possess similar mutational landscapes, with frequent variants in *ARID1A* (around 50% of CCOC), *PIK3CA*, and *PTEN*, which supports treating them together as a single histopathological entity.^4,11,12,13,14,15^ CCGC have an immune-rich tumor microenvironment contributed to by various mechanisms, including mismatch repair (MMR) deficiency, estimated at 5% to 10% of CCOC, frequent *ARID1A* variants, and upregulation of IL-6 and other pro-inflammatory cytokine signaling. These features as well as a high level of programmed cell death 1 ligand 1 (PD-L1) and PD-L2 expression suggest a possible role for immune checkpoint inhibitors (ICIs).^4,11,14,16,17,18,19,20,21^

Furthermore, anecdotal evidence from ICI trials performed in EOC suggested a superior response rate in CCOC than other EOC subtypes.^22,23,24,25^ For example, in the randomized phase 2 NRG GY003 trial, comparing ipilimumab and nivolumab with nivolumab in EOC, patients with CCOC were 5-fold more likely to respond than other subtypes.^25^ To our knowledge, PEACOCC is the only study examining pembrolizumab monotherapy in CCGC to date.

We sought to assess clinical activity of the programmed cell death 1 protein (PD-1) inhibitor pembrolizumab in patients with advanced CCGC who have previously received at least 1 course of prior chemotherapy.

## Methods

The PEACOCC trial is sponsored by the University College London and was conducted in accordance with the Research Governance Framework for Health and Community Care, Medicines for Human Use (Clinical Trials) Regulations, and the Declaration of Helsinki.^26^ The study was reviewed and approved by Yorkshire & The Humber–Sheffield Research Ethics Committee and the Medicines and Healthcare products Regulatory Agency. All trial patients provided written informed consent. This study followed the Transparent Reporting of Evaluations With Nonrandomized Designs (TREND) reporting guideline.

### Patient Population

Eligible patients were 18 years or older and had a diagnosis of advanced CCGC, including ovarian (including primary peritoneal and fallopian tube), endometrial, vaginal, vulval, or cervical cancer. Mixed histology was allowed, provided that more than 50% of tumor specimen was of clear cell histology as determined by specialist gynecological histopathologist at trial sites. Patients must have received at least 1 prior course of platinum chemotherapy (including in the adjuvant setting). Prior therapy with an anti–PD-1/PD-L1 therapy was not permitted. Patients had an Eastern Cooperative Oncology Group (ECOG) performance status (PS) score of 0 or 1; measurable disease per Response Evaluation Criteria in Solid Tumors (RECIST) version 1.1, as assessed by the investigator; and disease amenable to biopsy. Patients with a known diagnosis of autoimmune disease, second malignancy, central nervous system metastases and/or carcinomatous meningitis, grade 2 or higher hypercalcaemia, untreated venous thrombosis, or hospitalization for bowel obstruction within 4 weeks were excluded.

### Study Design and Treatment

The PEACOCC trial is a UK multicenter, single-arm phase 2 clinical trial. All patients were enrolled to receive pembrolizumab, 200 mg, intravenously once every 3 weeks for up to 35 cycles. Treatment was continued until completion of 35 cycles, radiographic progression, unacceptable toxic effects, investigator’s discretion, or patient withdrawal of consent. Any patients with a confirmed radiological complete response (CR) could discontinue treatment providing they had been treated for at least 24 weeks with pembrolizumab and had at least 2 treatments with pembrolizumab beyond the date of CR. Patients treated with 35 cycles of pembrolizumab were eligible for retreatment at the time of disease progression. For further details, the trial protocol can be found in Supplement 1. Comprehensive longitudinal tissue sampling, including mandatory tumor biopsies, were collected throughout the study.

### Assessments

Tumor assessments were conducted using computed tomography imaging performed at baseline and at 6 and 12 weeks after treatment initiation and thereafter every 12 weeks until progression, as assessed by investigator according to RECIST version 1.1. Retrospective immunohistochemistry for MMR proteins (MLH1, PMS2, MSH2, and MSH6) as well as PD-1, PD-L1, ARID1A, and p53 was analyzed at Queen Mary’s University London and Health Services laboratories on 4-mm-thick formalin-fixed paraffin-embedded (FFPE) diagnostic tissue sections. Antibodies and their respective concentrations are shown in eTable 1 in Supplement 2. Sections were reviewed centrally by 2 independent specialist gynecological histopathologists (C.S.H. and R.A.).

Hematoxylin-eosin staining was performed using the Leica ST5010 Autostainer XL (Leica Biosystems). FFPE sections were deparaffinised in xylene (X/0200/17; Thermo Fisher Scientific), then rehydrated with successive mixtures of ethanol (M/455/17; Thermo Fisher Scientific) of decreasing concentrations (100%, 90%, and 70%) followed by wash in water. Slides were submerged in acid alcohol, 1% (3803651E; Leica Biosystems) followed by eosin (3801601E; Leica Biosystems) and then hematoxylin (3801542E; Leica Biosystems) before dehydration with successive mixtures of ethanol of increasing concentration (70%, 90%, and 100%) and finally xylene.

### Statistical Analysis

The primary end point was progression-free survival (PFS) rate at 12 weeks, defined as the number of patients alive with CR or partial response (PR) or stable disease (SD) maintained at 12 weeks using RECIST version 1.1; numbers and percentages are presented with exact binomial confidence intervals. A total of 48 eligible patients were required to detect a 12-week PFS rate of 33% or greater and exclude an unacceptable rate of 15% or less, with 90% power and 1-sided 5% significance level (A’Hern design).^27^ Secondary end points included objective response rate (ORR), duration of response, PFS, overall survival (OS), safety (Common Terminology Criteria for Adverse Events version 5.0), and quality of life (Functional Assessment of Cancer Therapy–Ovarian version 4.0). Kaplan-Meier methods were used to describe PFS and OS, and mixed modeling was used to describe quality of life. Long-term patient follow-up is now available for estimates of OS and exploratory analyses by immunohistochemistry antibodies. Patients were enrolled from March 2019 to October 2021, with data collected until July 2024. Data analyses were performed using SAS software version 9.4 (SAS Institute) and GraphPad Prism, version 9.3.1 for Windows (GraphPad).

## Results

### Patient Characteristics

**Figure 1.**
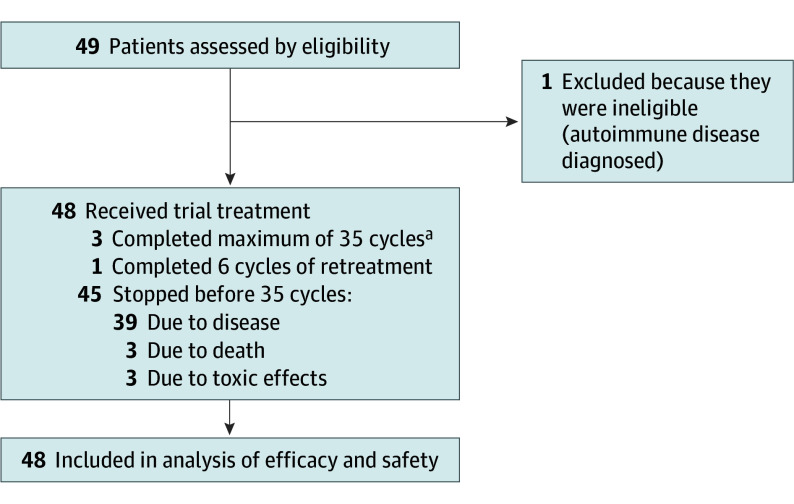
CONSORT Study Flow Diagram ^a^Maximum permitted protocol treatment duration (initial treatment stage).

A total of 49 patients were enrolled across 5 UK centers (eTable 2 in Supplement 2). One ineligible patient was withdrawn following enrollment due to a diagnosis of Graves disease prior to receiving their first cycle of trial treatment; patients with active autoimmune disease were ineligible. This patient’s data were excluded from the analysis population. The CONSORT study flow diagram is presented in Figure 1.

Baseline characteristics are summarized in Table 1. Of 48 included patients, the median (range) age at registration was 58.5 (32-77) years; 26 (54%) had an ECOG PS score of 0 and 22 (46%) had an ECOG PS of 1. A total of 41 patients (85%) had ovarian, 6 (13%) had endometrial, and 1 (2%) cervical clear cell cancer. Patients had received a median (range) of 3 (1-6) prior courses of systemic therapy, including 19 (40%) with a prior anti-angiogenic therapy, and 19 (40%) had a platinum-free interval greater than 12 months.

**Table 1.** Baseline Characteristics

Characteristic	Patients, No. (%) (N = 48)
Age, median (range), y	58.5 (32-77)
ECOG performance status score	
0	26 (54)
1	22 (46)
Type of cancer	
Ovarian CCC	41 (85)
Endometrial CCC	6 (13)
Cervical CCC	1 (2)
FIGO stage at diagnosis	
I	18 (38)
II	9 (19)
III	14 (29)
IV	7 (15)
Previous surgery	
Yes	43 (90)
No	5 (10)
Previous radiotherapy	
Yes	11 (23)
No	37 (77)
Previous systemic therapy	
Yes	48 (100)
No	0
Courses of previous systemic therapy, median (range)	3 (1-6)
Platinum-free interval, mo	
<6	14 (29)
6-12	15 (31)
>12	19 (40)
Brain metastasis at baseline	
Yes	0
No	48 (100)
Cancer antigen 125, median (range), U/mL	117.5 (5-4375)

Abbreviations: CCC, clear cell cancer; ECOG, Eastern Cooperative Oncology Group; FIGO, International Federation of Gynecology and Obstetrics.

SI conversion factor: To convert cancer antigen 125 to kU/L, multiply by 1.

### Efficacy

The trial’s primary end point was met: 42% (95% CI, 28-57) of eligible patients who received pembrolizumab achieved disease control and were progression-free at 12 weeks, with the 95% CI exceeding the prestated lower bound of 15%. In total, at 12 weeks, there were 10 (21%) with PR, 10 (21%) with SD, 27 (56%) who progressed and/or died, and 1 (2%) with missing data, giving a 12-week ORR of 21% (95% CI, 10-35). The best ORR with pembrolizumab use was 25% (95% CI, 14-40), with 12 partial responses in total.

Imaging assessment to assess 12-week disease status was not feasible for 1 patient who died after 12 weeks. This patient was included as having no confirmed disease control at 12 weeks for the primary analysis. Excluding this patient, in a sensitivity analysis, 20 of 47 patients achieved disease control and were progression free at 12 weeks (43%; 95% CI, 28-58), with a 12-week ORR of 21% (95% CI, 11-36) and a best ORR of 26% (95% CI, 14-40).

**Figure 2.**
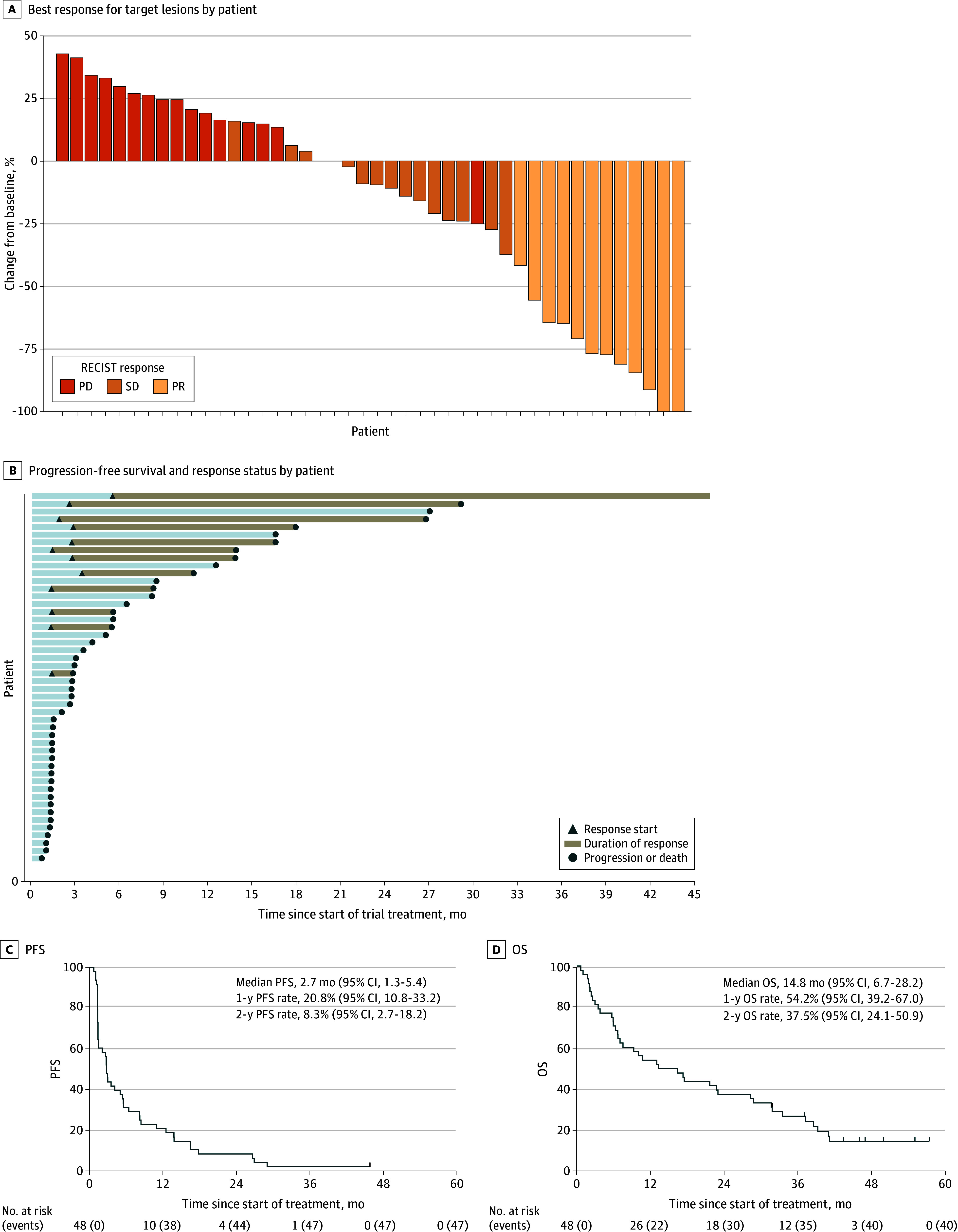
Treatment Efficacy A total of 4 eligible patients had no imaging after treatment; 3 died and 1 could not be imaged due to intercurrent illness. OS indicates overall survival; PD, progressive disease; PFS, progression-free survival; PR, partial response; SD, stable disease.

Treatment responses are detailed in Table 2, with a waterfall plot of best target lesion response in Figure 2A and a swimmer plot in Figure 2B. In patients with confirmed disease control at 12 weeks (n = 20), the median duration of disease control from start of pembrolizumab was 11.7 months (95% CI, 5.5-16.5). In patients achieving disease response (n = 12), the median time from start of pembrolizumab to disease response was 2.2 months (95% CI, 1.3-2.8) and the median duration of disease response was 13.1 months (95% CI, 4.2-26.5).

**Table 2.** Treatment Response

Outcome	Patients, No. (%) (N = 48)	
	12-wk Response	Best response^a^
Treatment response		
Complete response	0	0
Partial response	10 (21)	12 (25)
Stable disease	10 (21)	16 (33)
Progressive disease	19 (40)	16 (33)
Progressed and died	5 (10)	NA
Died^b^	3 (6)	3 (6)
Missing^c^	1 (2)	1 (2)
Disease control rate	20 (42)	28 (58)
Disease response rate	10 (21)	12 (25)

Abbreviation: NA, not applicable.

^a^ Best response at any time while taking treatment; this was achieved at the 6-week and/or 12-week assessment in all patients.

^b^ For these 3 patients no imaging assessment was done, and the patients died before 12 weeks.

^c^ For this patient, no imaging assessment was done, and the patient died after 12 weeks.

After a median follow-up of 46.9 months (95% CI, 43.4-55.0; data cut-off July 2024), 40 patients had died, 7 were alive following disease progression, and 1 was alive and progression free. The median PFS was 2.7 months (95% CI, 1.3-5.4), with 1-year and 2-year PFS rates of 21% (95% CI, 11-33) and 8% (95% CI, 3-18), respectively (Figure 2C). The median OS was 14.8 months (95% CI, 6.7-28.2), with 1-year and 2-year OS rates of 54% (95% CI, 39-67) and 38% (95% CI, 24-51), respectively (Figure 2D). Three patients (6%) completed the maximum 2 years of trial treatment, with 2 achieving PR and 1 achieving SD. One patient was eligible and enrolled for retreatment with pembrolizumab following first progression. This patient received 6 cycles of pembrolizumab, which was then stopped due to grade 3 immune-related hepatitis. Best response to retreatment was SD, and second progression occurred 7.1 months following first progression. The second patient was not retreated at disease progression, and the third patient continued in the trial with PR.

### Compliance and Safety

All 48 patients who were treated stopped or completed pembrolizumab, receiving a median (IQR) of 4.5 (2.5-12) cycles. A total of 39 patients (81%) stopped treatment due to disease progression, 3 (6%) due to toxic effects (grade 3 acute kidney injury; grade 3 encephalitis; grade 3 metabolic [diabetic] ketoacidosis), 3 (6%) died due to CCGC, and 3 (6%) completed the maximum 35 cycles.

A total of 33 patients (69%) experienced at least 1 treatment-related adverse event (TRAE), summarized in Table 3. Nine patients (19%) experienced a grade 3 TRAE, with raised alanine aminotransferase concentrations and hyperthyroidism being the most common, each seen in 2 patients (4%). No grade 4 or grade 5 TRAEs were observed. Grade 1 to 5 TRAEs and grade 3 or higher adverse events of any relatedness are detailed in eTables 3 and 4 in Supplement 2, respectively.

**Table 3.** Treatment-Related Adverse Events^a^

Adverse event^b^	Patients, No. (%) (N = 48)				
	Grade 1	Grade 2	Grade 3	Grade 4	Grade 5
Adverse event					
Acute kidney injury	0	0	1 (2)	0	0
Alanine aminotransferase increased	1 (2)	2 (4)	2 (4)^c^	0	0
Alkaline phosphatase increased	2 (4)	2 (4)	1 (2)	0	0
Anaemia	0	0	1 (2)	0	0
Anorexia	4 (8)	3 (6)	0	0	0
Aspartate aminotransferase increased	1 (2)	2 (4)	1 (2)^c^	0	0
Cough	3 (6)	0	0	0	0
Diarrhea	8 (17)	1 (2)	0	0	0
Dry mouth	4 (8)	1 (2)	0	0	0
Dry skin	4 (8)	0	0	0	0
Dysgeusia	3 (6)	0	0	0	0
Fatigue	8 (17)	6 (13)	0	0	0
GGT increased	0	0	1 (2)^c^	0	0
Hyperthyroidism	3 (6)	2 (4)	2 (4)	0	0
Hypothyroidism	3 (6)	7 (15)	0	0	0
Immune-related hepatitis	0	0	1 (2)^c^	0	0
Metabolism and nutrition disorders: ketoacidosis	0	0	1 (2)	0	0
Mucositis oral	4 (8)	0	0	0	0
Nausea	5 (10)	2 (4)	0	0	0
Nervous system disorders: noninfective encephalitis	0	0	1 (2)	0	0
Pruritus	8 (17)	2 (4)	0	0	0
Rash maculopapular	6 (13)	1 (2)	0	0	0
Skin and subcutaneous tissue disorders: rash	2 (4)	1 (2)	0	0	0
Vomiting	3 (6)	0	0	0	0
Any adverse event^d^	10 (21)	14 (29)	9 (19)^c^	0	0

Abbreviation: GGT, γ-glutamyltransferase.

^a^ Includes grade 1 and 2 adverse events observed in more than 5% of patients and any grade 3 to 5 adverse events.

^b^ Maximum grade of each term per patient (ie, each patient may be counted for more than 1 adverse event term).

^c^ A total of 8 patients had 1 grade 3 adverse event; 1 patient had 4 different grade 3 adverse events (alanine aminotransferase increased, aspartate aminotransferase increased, GGT increased, and immune-related hepatitis).

^d^ Maximum grade per patient (ie, each patient counted only once).

A total of 46 patients (96%) completed baseline and 29 (60%) completed end-of-treatment quality of life FACT-O questionnaires. There was no strong evidence of a change in the total score or in the physical, social, emotional, or functional well-being subscales over time (eTable 5 and eFigure 1 in Supplement 2).

### Exploratory Biomarker Analyses

Immunohistochemistry analysis of diagnostic FFPE tumor samples were performed for MMR protein (MLH1, PMS2, MSH2, MSH6), ARID1A, and p53 expression status in 46 patients and PD-1, PD-L1, and PD-1/PD-L1 combined in 45 patients. A consensus was reached from independent central histopathological review of the results. Only 1 patient (2%) demonstrated MMR deficiency with isolated loss of PMS2 expression. This patient achieved PR to pembrolizumab, progressed at 2.8 months, and died at 28.2 months. Six patient tumors (13%) had aberrant p53 expression, 10 (22%) had ARID1A loss, 4 (9%) were PD-1 positive, 10 (22%) were PD-L1 positive, and 4 (9%) were positive for both PD-1 and PD-L1. In these small subgroups, there was no evidence of a difference in PFS (eFigure 2 in Supplement 2) or OS (eFigure 3 in Supplement 2) for patients with tumors that harbored MMR deficiency, ARID1A loss, aberrant expression of p53, or expressed PD-1, PD-L1 or a combination of PD-1/PD-L1 in this study.

## Discussion

To our knowledge, the PEACOCC trial is the first evaluating the clinical benefit and safety of PD-1 inhibition exclusively in patients with CCGC (85% with CCOC). The study met its primary end point, demonstrating a 12-week PFS rate of 42% (95% CI, 28-57), with the 95% CI exceeding the prestated lower bound of 15%. Additionally, despite recruiting a heavily pretreated population (median of 3 prior courses of treatment), of whom 40% had received prior anti-angiogenic and 60% had relapsed within 12 months, clinically meaningful and durable benefit was observed, with an ORR of 25% and a median duration of response and median OS of 13.1 months and 14.8 months, respectively. Three patients (6%), including 2 with PR and 1 with SD, completed 2 years of pembrolizumab, demonstrating prolonged benefit achievable even with best response of SD: 1 was retreated, 1 subsequently progressed without retreatment, and 1 continues with PR. Overall, pembrolizumab was tolerated with no TRAEs above grade 3. These results compare favorably with reported clinical outcomes associated with standard chemotherapy in this clinical setting.^3,9,10^

CCGC is a distinct subtype of gynecological cancer, with unique histology, molecular profile, and clinical behavior irrespective of organ of origin. As diagnosis of rare subtypes of gynecological cancer is often difficult and open to misinterpretation, histology for each patient was reviewed and confirmed by a specialist gynecological histopathologist ensuring that 50% or more of the tumor was of clear cell type. Effective treatment options are lacking, and no specific guidance exists on optimal management or biomarker selection in advanced disease.

In addition to the PEACOCC trial, 3 other studies evaluating ICI treatment exclusively in CCGC have been conducted. MOCCA, a randomized phase 2 trial comparing single-agent durvalumab (PD-L1 inhibitor; n = 31) with physician’s choice chemotherapy (n = 16), did not demonstrate a difference in PFS, ORR, or clinical benefit rate after a median follow-up of 20 months.^28^ However, histological review of clear cell diagnosis was not carried out and the proportion of patients with platinum-resistant disease is not stated, factors that could have influenced the unexpectedly high response rate observed in the chemotherapy arm (19%). In contrast, another randomized study in CCOC showed an ORR of 0% in the chemotherapy arm.^29^ INOVA, a single-arm trial of sintilimab (PD-1 inhibitor) combined with bevacizumab, enrolled 41 patients with CCOC, 37 of whom were evaluable and observed an ORR of 41%, a median PFS of 6.3 months, and an immature OS at 6.3 months’ median follow-up.^30^ Preliminary data from BrUOG-354 of patients with ovarian and other extrarenal clear cell cancers has been presented: 14 patients were treated with nivolumab with an ORR of 14%, a median PFS of 2.2 months, and a median OS of 17.0 months: 36 patients were treated with nivolumab/ipilimumab, with an ORR of 33%, a median PFS of 5.6 months, and a median OS of 24.6 months.^31^ The Keynote-100 phase 2 trial evaluating single-agent pembrolizumab in ovarian cancer reported a 15% ORR in the CCOC subset.^24^ The phase 2 NRG GY003 trial, comparing ipilimumab and nivolumab with nivolumab in EOC, showed patients with CCOC were 5-fold more likely to respond than other subtypes^25^ and CCEC had the greatest reduction in risk of death (hazard ratio, 0.33) among other histological subtypes when treated with pembrolizumab/lenvatinib compared with standard of care chemotherapy in the Keynote-775 trial.^32^ Taken together, these results show that PD-1 inhibitors have consistently demonstrated clinical activity in advanced, pretreated CCGC, in keeping with our reported results from the PEACOCC trial. Final results from ongoing phase 2 studies involving PD-1/PDL-1 inhibitor combinations are awaited.

Molecular characterization of CCGC is predominantly derived from genomic and RNA analyses. A number of distinct prognostic mutational signatures and gene expression profiles have been identified, although none are currently used in clinical practice.^33,34^ More recently, an understanding of the importance of the composition of immune cell infiltrates and PD-1 pathway protein expression within the tumor microenvironment have been examined and have been shown to vary according to mutational status of prognostic markers, for example ARID1A and p53, in CCGC.^18,35,36^ MMR deficiency is an important predictor of ICI sensitivity and has been reported more frequently in CCGC than other common gynecological histological subtypes.^4,11,14,17,18,19,20,21^

We investigated MMR, ARID1A, p53, PD-1, PD-L1 (combined positive score ≥1 deemed PD-L1 positive; combined positive score <1 deemed PD-L1 negative) and combined PD-1/PD-L1 protein expression in archival tumor samples to explore their relationship with clinical outcome following ICI treatment. Only 1 of 46 patients with evaluable diagnostic tumor tissue had an MMR-deficient pattern, ruling out MMR deficiency as an explanation for the clinical activity observed in the PEACOCC trial. Small numbers limit interpretation of these exploratory biomarkers with ICI response and no definitive conclusions can be reached. Specifically, no clear association with PD-L1 positivity and pembrolizumab response as observed in Keynote-100 was seen.

We have demonstrated that a trial of a rare histological subtype of gynecological cancer is feasible even with mandated translational sample collection including tumor biopsies, with enrollment occurring over a 30-month span in large part during the COVID-19 pandemic. Our tissue collection including contemporaneous trial biopsies allows further translational study of the molecular and immune landscape.

### Limitations

The main limitations of our study are the single-arm design and moderate sample size of the trial. The available contemporary data from the 2 randomized CCOC trials with physician’s choice control arms^28,29^ provide a benchmark to understand the range of benefit of standard chemotherapy in this setting. Additionally, the consistent clinical benefit, although variable in magnitude, for a number of different PD-1 inhibitors in different clinical trial settings for CCGC supports the conclusions from our study.

## Conclusions

Pembrolizumab has robust, durable clinical benefit in patients with heavily pretreated CCGC and was tolerated in the overall population. The clinical activity was observed in a predominantly MMR-proficient population (45 of 46 patients). Clinical outcomes are superior to historical data for current standard of care chemotherapy, and translational research to identify those patients who benefit most is underway. Further evaluation of anti–PD-1 monotherapy or PD-1 inhibitor combinations in randomized clinical trials is justified for this poor-prognosis gynecological cancer population with a significant unmet clinical need.

## Notes

Supplement 1. Trial Protocol

Supplement 2. **eTable 1.** Antibodies Used for Immunohistochemistry Analysis **eTable 2.** Recruitment **eTable 3.** Treatment-Related Adverse Events (Grade 1-5 in Any Patient) **eTable 4.** Adverse Events of Any Relatedness (Grade 3-5 in Any Patient) **eTable 5.** Quality of Life (QoL) by Visit (A) and Change From Baseline (B) **eFigure 1.** Quality of Life by Visit (A), Change From Baseline (B) and Change From Baseline by Disease Status at 12 Weeks (C) **eFigure 2.** Progression-Free Survival by ARID1A (A), MMR (B), p53 (C), PD1 (D), PDL1 (E) and PD1/PDL1 Combined (F) **eFigure 3.** Overall Survival by ARID1A (A), MMR (B), p53 (C), PD1 (D), PDL1 (E) and PD1/PDL1 Combined (F)

Supplement 3. Data Sharing Statement

## References

[1] Hasegawa K, Nagao S, Yasuda M, . Gynecologic Cancer InterGroup (GCIG) consensus review for clear cell carcinoma of the uterine corpus and cervix. Int J Gynecol Cancer. 2014;24(9)(suppl 3):S90-S95. doi:10.1097/IGC.0000000000000297 25341588

[2] Shu CA, Zhou Q, Jotwani AR, . Ovarian clear cell carcinoma, outcomes by stage: the MSK experience. Gynecol Oncol. 2015;139(2):236-241. doi:10.1016/j.ygyno.2015.09.016 26404183 PMC4632203

[3] Glasspool RM, McNeish IA. Clear cell carcinoma of ovary and uterus. Curr Oncol Rep. 2013;15(6):566-572. doi:10.1007/s11912-013-0346-0 24114188

[4] Nigon E, Lefeuvre-Plesse C, Martinez A, . Clinical, pathological, and comprehensive molecular analysis of the uterine clear cell carcinoma: a retrospective national study from TMRG and GINECO network. J Transl Med. 2023;21(1):408. doi:10.1186/s12967-023-04264-7 37353806 PMC10288685

[5] Chan JK, Teoh D, Hu JM, Shin JY, Osann K, Kapp DS. Do clear cell ovarian carcinomas have poorer prognosis compared to other epithelial cell types? a study of 1411 clear cell ovarian cancers. Gynecol Oncol. 2008;109(3):370-376. doi:10.1016/j.ygyno.2008.02.006 18395777

[6] Anuradha S, Webb PM, Blomfield P, . Survival of Australian women with invasive epithelial ovarian cancer: a population-based study. Med J Aust. 2014;201(5):283-288. doi:10.5694/mja14.00132 25163381

[7] Mackay HJ, Brady MF, Oza AM, ; Gynecologic Cancer InterGroup. Prognostic relevance of uncommon ovarian histology in women with stage III/IV epithelial ovarian cancer. Int J Gynecol Cancer. 2010;20(6):945-952. doi:10.1111/IGC.0b013e3181dd0110 20683400

[8] Miyamoto M, Takano M, Goto T, . Clear cell histology as a poor prognostic factor for advanced epithelial ovarian cancer: a single institutional case series through central pathologic review. J Gynecol Oncol. 2013;24(1):37-43. doi:10.3802/jgo.2013.24.1.37 23346312 PMC3549506

[9] Pather S, Quinn MA. Clear-cell cancer of the ovary—is it chemosensitive? Int J Gynecol Cancer. 2005;15(3):432-437. 15882166 10.1111/j.1525-1438.2005.15305.x

[10] Takano M, Sugiyama T, Yaegashi N, . Low response rate of second-line chemotherapy for recurrent or refractory clear cell carcinoma of the ovary: a retrospective Japan Clear Cell Carcinoma Study. Int J Gynecol Cancer. 2008;18(5):937-942. doi:10.1111/j.1525-1438.2007.01158.x 18081792

[11] Anglesio MS, George J, Kulbe H, ; Australian Ovarian Cancer Study Group. IL6-STAT3-HIF signaling and therapeutic response to the angiogenesis inhibitor sunitinib in ovarian clear cell cancer. Clin Cancer Res. 2011;17(8):2538-2548. doi:10.1158/1078-0432.CCR-10-3314 21343371

[12] Ikeda Y, Oda K, Aburatani H, Kawana K, Osuga Y, Fujii T. Non-diethylstilbestrol exposed vaginal clear cell adenocarcinoma has a common molecular profile with ovarian clear cell adenocarcinoma: a case report. Gynecol Oncol Rep. 2014;10:49-52. doi:10.1016/j.gynor.2014.05.006 26082939 PMC4458747

[13] Wiegand KC, Lee AF, Al-Agha OM, . Loss of BAF250a (ARID1A) is frequent in high-grade endometrial carcinomas. J Pathol. 2011;224(3):328-333. doi:10.1002/path.2911 21590771

[14] Wiegand KC, Shah SP, Al-Agha OM, . ARID1A mutations in endometriosis-associated ovarian carcinomas. N Engl J Med. 2010;363(16):1532-1543. doi:10.1056/NEJMoa1008433 20942669 PMC2976679

[15] Zorn KK, Bonome T, Gangi L, . Gene expression profiles of serous, endometrioid, and clear cell subtypes of ovarian and endometrial cancer. Clin Cancer Res. 2005;11(18):6422-6430. doi:10.1158/1078-0432.CCR-05-0508 16166416

[16] Ghasemi D, Ameli F, Nili F, Edjtemaei R, Sheikhhasani S. Immunohistochemical expression of PD-L1 and its correlation with microsatellite status in endometrial and ovarian clear cell carcinomas: a cross-sectional study. BMC Cancer. 2022;22(1):1362. doi:10.1186/s12885-022-10478-7 36581850 PMC9801577

[17] Atwal A, Snowsill T, Dandy MC, . The prevalence of mismatch repair deficiency in ovarian cancer: a systematic review and meta-analysis. Int J Cancer. 2022;151(9):1626-1639. doi:10.1002/ijc.34165 35792468 PMC9539584

[18] Devlin MJ, Miller R, Laforets F, . The tumor microenvironment of clear-cell ovarian cancer. Cancer Immunol Res. 2022;10(11):1326-1339. doi:10.1158/2326-6066.CIR-22-0407 36095166 PMC9627265

[19] Li L, Li M, Jiang Z, Wang X. *ARID1A* mutations are associated with increased immune activity in gastrointestinal cancer. Cells. 2019;8(7):678. doi:10.3390/cells8070678 31277418 PMC6678467

[20] Oda K, Hamanishi J, Matsuo K, Hasegawa K. Genomics to immunotherapy of ovarian clear cell carcinoma: unique opportunities for management. Gynecol Oncol. 2018;151(2):381-389. doi:10.1016/j.ygyno.2018.09.001 30217369 PMC7526052

[21] Shen J, Ju Z, Zhao W, . ARID1A deficiency promotes mutability and potentiates therapeutic antitumor immunity unleashed by immune checkpoint blockade. Nat Med. 2018;24(5):556-562. doi:10.1038/s41591-018-0012-z 29736026 PMC6076433

[22] Disis ML, Taylor MH, Kelly K, . Efficacy and safety of avelumab for patients with recurrent or refractory ovarian cancer: phase 1b results from the JAVELIN solid tumor trial. JAMA Oncol. 2019;5(3):393-401. doi:10.1001/jamaoncol.2018.6258 30676622 PMC6439837

[23] Hamanishi J, Mandai M, Ikeda T, . Safety and antitumor activity of anti-PD-1 antibody, nivolumab, in patients with platinum-resistant ovarian cancer. J Clin Oncol. 2015;33(34):4015-4022. doi:10.1200/JCO.2015.62.3397 26351349

[24] Matulonis UA, Shapira-Frommer R, Santin AD, . Antitumor activity and safety of pembrolizumab in patients with advanced recurrent ovarian cancer: results from the phase II KEYNOTE-100 study. Ann Oncol. 2019;30(7):1080-1087. doi:10.1093/annonc/mdz135 31046082

[25] Zamarin D, Burger RA, Sill MW, . Randomized phase II Trial of nivolumab versus nivolumab and ipilimumab for recurrent or persistent ovarian cancer: an NRG Oncology study. J Clin Oncol. 2020;38(16):1814-1823. doi:10.1200/JCO.19.02059 32275468 PMC7255977

[26] Dale O, Salo M. The Helsinki Declaration, research guidelines and regulations: present and future editorial aspects. Acta Anaesthesiol Scand. 1996;40(7):771-772. doi:10.1111/j.1399-6576.1996.tb04530.x 8874560

[27] A’Hern RP. Sample size tables for exact single-stage phase II designs. Stat Med. 2001;20(6):859-866. doi:10.1002/sim.721 11252008

[28] Tan DSP, Choi CH, Ngoi N, . A multicenter phase II randomized trial of durvalumab (D) versus physician’s choice chemotherapy (PCC) in patients (pts) with recurrent ovarian clear cell adenocarcinoma (MOCCA/APGOT-OV2/GCGS-OV3). J Clin Oncol. 2022;40(16)(suppl):5565. doi:10.1200/JCO.2022.40.16_suppl.5565

[29] Glasspool RMI, Westermann A, Hinsley S, . 596 randomised phase II study of nintedanib (BIBF1120) compared to chemotherapy in patients with recurrent clear cell carcinoma of the ovary or endometrium. (NICCC/ENGOT-OV36). Int J Gynecol Cancer. 2020;30:A127-A128.

[30] Gao Q, Liu X, Xia B, . Efficacy of sintilimab plus bevacizumab in recurrent/persistent ovarian clear cell carcinoma (INOVA): a multicenter, single-arm, phase II trial. Gynecol Oncol. 2024;190(suppl 1):S37. doi:10.1016/j.ygyno.2024.07.059 39276785

[31] Dizon DS, Mathews CA, David SM, . Final results of BrUOG 354: a randomized phase II trial of nivolumab alone or in combination with ipilimumab for people with ovarian and other extra-renal clear cell carcinomas. J Clin Oncol. 2024;42(17)(suppl). doi:10.1200/JCO.2024.42.17_suppl.LBA5500

[32] Makker V, Colombo N, Casado Herráez A, ; Study 309–KEYNOTE-775 Investigators. Lenvatinib plus pembrolizumab for advanced endometrial cancer. N Engl J Med. 2022;386(5):437-448. doi:10.1056/NEJMoa2108330 35045221 PMC11651366

[33] Stružinská I, Hájková N, Hojný J, . A comprehensive molecular analysis of 113 primary ovarian clear cell carcinomas reveals common therapeutically significant aberrations. Diagn Pathol. 2023;18(1):72. doi:10.1186/s13000-023-01358-0 37303048 PMC10259037

[34] Heong V, Tan TZ, Miwa M, . A multi-ethnic analysis of immune-related gene expression signatures in patients with ovarian clear cell carcinoma. J Pathol. 2021;255(3):285-295. doi:10.1002/path.5769 34322886 PMC9539643

[35] Khalique S, Nash S, Mansfield D, . Quantitative assessment and prognostic associations of the immune landscape in ovarian clear cell carcinoma. Cancers (Basel). 2021;13(15):3854. doi:10.3390/cancers13153854 34359755 PMC8345766

[36] Pejovic TJS, Campbell S, Tailor D, . Study of tumor microenvironment of ovarian clear cell carcinoma. Cancer Res. 2023;83:79. doi:10.1158/1538-7445.AM2023-79

