# Supplementary material for: Pembrolizumab in Patients With Advanced Clear Cell Gynecological Cancer: A Phase 2 Nonrandomized Clinical Trial
Source: JAMA Oncol. 2025 Feb 6;11(4):377–85. doi: 10.1001/jamaoncol.2024.6797 (PMC11803509; doi:10.1001/jamaoncol.2024.6797)
Supplement: Supplement 1. — Trial Protocol [file jamaoncol-e246797-s001.pdf]

DocuSign Envelope ID: F89E9DEB-F629-4294-B1D8-B570A4FACE07

National Cancer Research Institute

PEACOCC

A phase II study of PEmbrolizumab in patients with Advanced gynaeCOlogical Clear Cell Cancer

Trial Sponsor:	University College London
Trial Sponsor reference:	UCL/17/0672
Trial funder:	Merck Sharp & Dohme (MSD) Limited
MSD reference:	MISP 55357
Clinicaltrials.gov no:	NCT03425565
EUDRACT no:	2017-004168-36
IRAS no:	236786
Protocol version no:	5.0
Protocol version date:	23rd August 2022

DocuSign Envelope ID: F89E9DEB-F629-4294-B1D8-B570A4FACE07

Protocol 5.0, 23rd August 2022 Authorisation signatures:

Name & Role:	Signature:	Date authorised:
Chief Investigator:<br>Dr. Rebecca Kristeleit<br>Consultant Medical Oncologist	DocuSigned by:<br>4DBD2B2A7BC9498...	08-Oct-2022
Laura Farrelly<br>Trials Group Lead, UCL CTC	DocuSigned by:<br>9B0271D91DB9499...	06-Oct-2022

Please note: This trial protocol must not be applied to patients treated outside the PEACOCC trial. The Cancer Research UK & UCL Cancer Trials Centre (UCL CTC) can only ensure that approved trial investigators are provided with amendments to the protocol.

DocuSign Envelope ID: F89E9DEB-F629-4294-B1D8-B570A4FACE07

COORDINATING CENTRE:

For general queries, supply of trial documentation and central data management please contact:

PEACOCC Trial Co-ordinator
Cancer Research UK & UCL Cancer Trials Centre
90 Tottenham Court Road
London
W1T 4TJ
United Kingdom

Tel: +44 (0) 20 7679 9284
09:00 to 17:00 Monday to Friday, excluding Bank Holidays
Email: ctc.peacocc@ucl.ac.uk

Other trial contacts:

Chief Investigator: Dr Rebecca Kristeleit
Address: Guy's and St Thomas' NHS Foundation Trust (GSTT)
Great Maze Pond
London
SE1 9RT

Chief Clinical investigator: Dr Rowan Miller
Address: University College London Hospital
250 Euston Rd
London
NW1 2BU

Trial Management Group (TMG):

Dr Rebecca Kristeleit	Consultant Medical Oncologist	GSTT, London
Dr Rowan Miller	Consultant Medical Oncologist	UCLH & St Bart's Hospital
Nicholas Counsell	Medical Statistician	CR UK & UCL Cancer Trial Centre
Laura Farrelly	Trials Group Lead	CR UK & UCL Cancer Trial Centre
Laura Hughes	Trial Co-ordinator	CR UK & UCL Cancer Trial Centre
Prof. Charlie Gourley	Consultant Medical Oncologist	The University of Edinburgh
Dr Andrew Clamp	Consultant Medical Oncologist	The Christie, Manchester
Dr Rene Roux	Consultant Medical Oncologist	Churchill Hospital, Oxford
Dr Marcia Hall	Consultant Medical Oncologist	Mount Vernon Cancer Centre
Lesley Sage	Patient Representative	-
Dr Rupali Arora	Consultant Gynaecological Pathologist	UCLH
Prof. Simon Herrington	Consultant Gynaecological Pathologist	Royal Infirmary of Edinburgh
Dr Michael John Devlin	Academic Clinical Lecturer	QMUL
Dr Sheeba Irshad	Consultant Medical Oncologist-Senior Clinical Lecturer	GSTT-Kings College London

DocuSign Envelope ID: F89E9DEB-F629-4294-B1D8-B570A4FACE07

TABLE OF CONTENTS

1. PROTOCOL SUMMARY **7**

1.1. SUMMARY OF TRIAL DESIGN 7
1.2 TRIAL SCHEMA 13

2. INTRODUCTION **14**

2.1. BACKGROUND 14
2.1.1. Clear cell gynaecological malignancies *14*
2.1.2. Pharmaceutical and Therapeutic Background *14*

3. RATIONALE **16**

3.1.1. Rationale for the Trial and Selected Patient Population *16*
3.1.2. Rationale for Dose Selection/Regimen/Modification *16*
3.1.3. Rationale for endpoints *18*
3.1.3.1. Efficacy Endpoints *18*
3.1.3.1.1. Primary *18*
3.1.3.1.2. Secondary *18*
3.1.3.1.3. Exploratory *18*

4. TRIAL DESIGN **20**

4.1. TRIAL OBJECTIVES 21
4.1.1. Primary *21*
4.1.2. Secondary *21*
4.1.3. Exploratory Objectives *21*
4.2. TRIAL ENDPOINTS 21
4.2.1. Primary *21*
4.2.2. Secondary *21*
4.2.3. Exploratory *22*
4.3. TRIAL ACTIVATION 23

5. SELECTION OF SITES/SITE INVESTIGATORS **24**

5.1. SITE SELECTION 24
5.1.1. Selection of Principal Investigator and other investigators at sites *24*
5.1.2. Training requirements for site staff *24*
5.2. SITE INITIATION AND ACTIVATION 25
5.2.1. Site initiation *25*
5.2.2. Required documentation *25*
5.2.3. Site activation letter *25*

6. INFORMED CONSENT **27**

7. SELECTION OF PATIENTS **29**

7.1. SCREENING LOG 29
7.2. PATIENT ELIGIBILITY 29
7.2.1. Inclusion criteria *29*
7.2.2. Exclusion criteria *31*
7.3. PREGNANCY AND BIRTH CONTROL 33
7.3.1. Pregnancy and birth control *33*

7.3.2. Risk of exposure to trial treatment during pregnancy 33
7.3.3. Pregnancy testing 34
7.3.4. Contraceptive Advice 34
7.3.5. Action to be taken in the event of a pregnancy 34
7.3.6. Long Term Infertility 35
7.3.7. Lactation 35

8. REGISTRATION PROCEDURES 36

8.1. REGISTRATION 36
8.1.1. Pre-registration Assessments (initial treatment) 36
8.1.2 RE-TREATMENT REGISTRATION 38
8.1.2.1 Re-treatment Pre-registration Assessments 38
8.2. INITIAL TRIAL DRUG SUPPLY 39

9. TRIAL TREATMENT 40

9.1. INVESTIGATIONAL MEDICINAL PRODUCTS 40
9.1.1. Packaging and Labelling Information 40
9.1.2. Clinical Supplies Disclosure 40
9.2. TREATMENT SUMMARY 41
9.3. TRIAL TREATMENT DETAILS 41
9.3.1. Timing of Dose Administration 41
9.3.2. Treatment Duration 42
9.3.3. Re-treatment Period 42
9.4. DOSE MANAGEMENT/ DELAYS NOT RELATED TO IMP ADVERSE EVENTS 44
9.5. MANAGEMENT OF ADVERSE EVENTS 44
9.6. MANAGEMENT OF OVERDOSES, TRIAL TREATMENT ERROR, OR OCCUPATIONAL EXPOSURE 51
9.7. SUPPORTIVE CARE 51
9.8. CONTRAINDICATIONS 52
9.8.1. Acceptable Concomitant Medications 52
9.8.2. Prohibited Concomitant Medications\Therapies 53
9.9. PHARMACY RESPONSIBILITIES 54
9.9.1. Storage and Handling Requirements 54
9.9.3. Study IMP Accountability 54
9.9.4. Returns and Reconciliation 54
9.10. 24 HOUR/OUT-OF-OFFICE HOURS EMERGENCY DRUG-SPECIFIC ADVICE 55
9.11. CLINICAL MANAGEMENT AFTER TREATMENT DISCONTINUATION 55
9.12. DRUG PROVISION DURING THE TRIAL 55

10. ASSESSMENTS/TRIAL PROCEDURES 56

10.1. PRE-REGISTRATION ASSESSMENTS/PROCEDURES 56
10.2. ASSESSMENTS DURING TREATMENT 56
10.2.1. Adverse Event (AE) Monitoring 57
10.2.2. Full Physical Exam 57
10.2.3. Vital Signs 57
10.2.4. ECOG Performance status 57
10.2.5. Laboratory Procedures/Assessments 57
10.3. TUMOUR IMAGING AND ASSESSMENT OF DISEASE 58
10.3.1. Evaluation of Efficacy 58
10.3.2. Tumour Assessment 58
10.3.2.1. CT scans with contrast to the chest, abdomen, pelvis, and brain 58
10.3.2.2. MRI Imaging scans 58
10.4. OTHER PROCEDURES 59

DocuSign Envelope ID: F89E9DEB-F629-4294-B1D8-B570A4FACE07

10.4.1. Withdrawal/ Discontinuation 59
10.4.2. Visit Requirements 59
10.4.2.1. Baseline 59
10.4.2.2. Treatment period 59
10.5. ASSESSMENTS ON COMPLETION OF TRIAL TREATMENT 60
10.6. ASSESSMENTS DURING FOLLOW UP (BEFORE PROGRESSION) 61
10.6.1. Survival Follow-up 61

11. EXPLORATORY BIOLOGICAL STUDIES 62

11.1. TUMOUR TISSUE COLLECTION AND EXPLORATORY RESEARCH BLOOD SAMPLING 62
11.1.1. Biomarker Sample Collection 62
11.1.2. Tumour Biopsies 62

12. DATA MANAGEMENT AND DATA HANDLING GUIDELINES 64

12.1. COMPLETING ELECTRONIC CASE REPORT FORMS (ECRFS) 64
12.2. MISSING DATA 64
12.3. TIMELINES FOR DATA COMPLETION 64
12.4. DATA QUERIES 65

13. PHARMACOVIGILANCE 66

13.1. DEFINITIONS 66
13.2. REPORTING PROCEDURES 67
13.2.1. Reporting of Adverse Events (AEs) 67
13.2.2. Reporting of Serious Adverse Events (SAEs) 68
13.3. SUSARS 71
13.4. ADVERSE EVENTS OF SPECIAL INTEREST 71
13.5. SAFETY MONITORING 72
13.6. PREGNANCY 72
13.7. DEVELOPMENT SAFETY UPDATE REPORTS (DSURS) 73

14. INCIDENT REPORTING AND SERIOUS BREACHES 74

14.1. INCIDENT REPORTING 74
14.2. SERIOUS BREACHES 74

15. TRIAL MONITORING AND OVERSIGHT 75

15.1. ON-SITE AND REMOTE MONITORING 75
15.2. CENTRALISED MONITORING 76
15.3. TRIGGERED' ON-SITE/REMOTE MONITORING 76
15.4. ESCALATION OF MONITORING ISSUES 77
15.5. OVERSIGHT COMMITTEES 77
15.5.1. Trial Management Group (TMG) 77
15.5.2. Trial Steering Committee (TSC) 77
15.5.3. Independent Data Monitoring Committee (IDMC) 77
15.5.4. Role of UCL CTC 77

16. WITHDRAWAL OF PATIENTS 79

16.1. PATIENT WITHDRAWAL/DISCONTINUATION CRITERIA 79
16.1.1. Discontinuation of Study Treatment after CR 79
16.1.2. Treatment beyond Equivocal Progression 80
16.2. FUTURE DATA COLLECTION 80

DocuSign Envelope ID: F89E9DEB-F629-4294-B1D8-B570A4FACE07

16.3. LOSSES TO FOLLOW-UP 80

17. TRIAL CLOSURE 82

17.1. END OF TRIAL 82
17.2. ARCHIVING OF TRIAL DOCUMENTATION 82
17.3. EARLY DISCONTINUATION OF TRIAL 82
17.4. WITHDRAWAL FROM TRIAL PARTICIPATION BY A SITE 82

18. STATISTICS 83

18.1. SAMPLE SIZE CALCULATION 83
18.2. STATISTICAL ANALYSIS 83
18.2.1. Analysis of main endpoint 83
18.2.2. Analysis of secondary endpoints and secondary analyses 83
18.2.2.1. Progression-free survival 83
18.2.2.2. Time to second disease progression (Re-treatment period) 83
18.2.2.3. Overall survival 84
18.2.2.4. Objective response at 12 weeks and best objective response 84
18.2.2.5. Duration of Response 84
18.2.2.6. Quality of Life 85
18.2.2.7. Safety Monitoring 85
18.3. INTERIM ANALYSES85

19. ETHICAL AND REGULATORY CONSIDERATIONS 86

19.1. ETHICAL APPROVAL 86
19.2. REGULATORY APPROVAL 86
19.3. SITE APPROVALS / CONFIRMATION OF CAPACITY & CAPABILITY 87
19.4. PROTOCOL AMENDMENTS 87
19.5. PATIENT CONFIDENTIALITY & DATA PROTECTION 87

20. SPONSORSHIP AND INDEMNITY 88

20.1. SPONSOR DETAILS 88
20.2. INDEMNITY 88

21. FUNDING 89

22. PUBLICATION POLICY 90

23. REFERENCES 91

APPENDIX 1: SCHEDULE OF PROCEDURES/ASSESSMENTS 94

APPENDIX 2: ABBREVIATIONS 97

APPENDIX 3: PROTOCOL VERSION HISTORY 99

1. PROTOCOL SUMMARY

1.1. Summary of Trial Design

**Title:**	A phase II study of **PE**mbrolizumab in patients with **A**dvanced gynae**CO**logical **C**lear **C**ell cancer
**Short Title/acronym:**	PEACOCC
**EUDRACT no:**	2017-004168-36
**Sponsor name & reference:**	University College London - UCL/17/0672
**Funder name & reference:**	Merck Sharp & Dohme (MSD) Limited<br>MISP 55357
**Clinicaltrials.gov no:**	NCT03425565
**Design:**	Multi-centre, single arm phase II trial.
**Overall aim:**	To determine whether treatment with pembrolizumab is effective in patients with advanced clear cell gynaecological cancers
**Primary endpoint:**	Progression-free survival rate at 12 weeks
**Secondary endpoints:**	Further assessment of antitumour activity including:<br>• progression-free survival<br>• time to second progression in patients within the re-treatment cohort<br>• overall survival<br>• objective response rate at 12 weeks<br>• best objective response rate<br>• duration of response<br>• safety (adverse events)<br>• quality of life.
**Exploratory Endpoints:**	To determine pharmacodynamics of mechanistic and predictive biomarkers in archival, pre, during

DocuSign Envelope ID: F89E9DEB-F629-4294-B1D8-B570A4FACE07

	and post-treatment tumour biopsies and circulating biomarkers (plasma, whole blood) according to emerging data, including:<br>• PD-1, PD-L1 expression and mutational load in tumour and association with response<br>• ARID1A, BRCA, MMR and PIK3CA tumour mutation status and association with response<br>• HRD (homologous recombination deficiency including somatic BRCA) tumour status and association with response<br>• Characterization of tumour infiltrating lymphocytes (TILs) as well as cytolytic activity and association with outcome<br>• Murine double minute 2/4 (MDM2/MDM4), EGFR in tumour and association with response.<br>• RNA Sequencing<br><br>Evaluate peripheral blood biomarkers and association with clinical outcomes, including:<br>• Immunophenotype circulating T cell subsets (naïve, effector, central memory and regulatory), proliferation, and activation markers<br>• cytokines and chemokines, with an emphasis on IL-6 pathway - indirectly via serum CRP and directly via phospho-STAT3 on IHC and IL6 transcription by qRTPCR,<br>• other immune-modulatory and checkpoint biomarkers (will include PD-1/PD-L1, 4-1BB, ICOS & LAG3)<br>• quantification and analysis of circulating free DNA<br>• germline BRCA status and mismatch repair (MMR) status<br>• Ca125 levels.
**Target accrual:**	48 patients

**Key inclusion & exclusion criteria**<br>**(N.B. see section 7.2 for full details):**	**Inclusion:**<br><br>1. Histological diagnosis of advanced gynaecological clear cell cancer including ovarian (including primary peritoneal and fallopian tube), endometrial, vaginal, vulval or cervical cancer.<br>2. Be willing and able to provide written informed consent/assent for the trial.<br>3. Age ≥18 years.<br>4. Patient must have at least one measurable lesion according to RECIST v1.1 in addition to a separate biopsiable lesion. Measurable lesions should be outside any prior radiation field unless progression has occurred at that site.<br>5. Evidence of radiological disease progression.<br>6. Patient is willing to provide tissue from a newly obtained core or excisional biopsy of a tumour lesion at baseline, 6-8 weeks after start of treatment and at the time of progression (patients are not eligible if the cancer cannot be safely biopsied at baseline).<br>7. Patient is willing to provide archival tissue.<br>8. ECOG Performance Status 0 or 1.<br>9. Patient has a life expectancy of at least 4 months from consent.<br>10. Received ≥ 1 line of prior chemotherapy.<br>11. Demonstrate adequate organ function.<br>12. For patients of childbearing potential, negative urine or serum pregnancy test prior to receiving the first dose of study medication.

DocuSign Envelope ID: F89E9DEB-F629-4294-B1D8-B570A4FACE07

	13. Patients of childbearing potential must be willing to use a highly effective method of contraception for the study duration required.<br><br>**Exclusion:**<br><br>1. Is currently participating in and receiving other study therapy or has participated in a study of an investigational agent and received study therapy or used an investigational device within 4 weeks of the first dose of treatment.<br><br>2. Prior anti-cancer monoclonal antibody (mAb), chemotherapy or targeted small molecule therapy within 4 weeks prior to study treatment Day 1 or who has not recovered (i.e., ≤ Grade 1 or at baseline) from adverse events due to agents administered more than 4 weeks earlier.<br><br>3. Radiation therapy within 2 weeks prior to starting study treatment.<br><br>4. Prior therapy with an anti-PD-1, anti-PD-L1 or anti-PD-L2 agent.<br><br>5. Known diagnosis of immunodeficiency or is receiving systemic steroid therapy at doses > 10mg prednisolone daily or equivalent or any other form of immunosuppressive therapy within 7 days prior to the first dose of trial treatment.<br><br>6. Has a known history of active bacillus tuberculosis (TB).<br><br>7. Has a known history of Hepatitis B (defined as Hepatitis B surface antigen [HBsAg] reactive) or known active Hepatitis C virus (defined as HCV RNA [qualitative] detected) infection.<br><br>8. Known history of Human Immunodeficiency Virus (HIV) (HIV type 1/2 antibodies).<br><br>9. Known hypersensitivity to pembrolizumab or any of its excipients.

DocuSign Envelope ID: F89E9DEB-F629-4294-B1D8-B570A4FACE07

	10. Known additional malignancy that is progressing or requires active treatment. If there is a history of a second malignancy and there is doubt regarding the aetiology of progressive disease, then a biopsy is required to determine the diagnosis.<br><br>11. Has known active central nervous system (CNS) metastases and/or carcinomatous meningitis.<br><br>12. Has active autoimmune disease that has required systemic treatment in the past 2 years *(i.e. with use of disease modifying agents, corticosteroids (at a dose >10mg predisolone daily or equivalent) or immunosuppressive drugs).*<br><br>13. Corrected serum calcium of > grade 1 hypercaelcemia (>2.9 mmol/L) despite maximal antihypercalcaemic therapy.<br><br>14. Has a known history of (non-infectious) pneumonitis that required steroids or has current pneumonitis.<br><br>15. Newly diagnosed venous thromboembolic event (eg pulmonary embolism, deep vein thrombosis DVT), unless patient has received at least 14 days of therapeutic dose anticoagulation for a new thromboembolic event and is suitable for continued therapeutic anticoagulation during trial participation.<br><br>16. History of arterial thrombosis (excluding the pulmonary artery), within 12 months prior to registration (if beyond 12 months prior to registration, patient may be included providing they have fully recovered clinically).<br><br>17. Active infection requiring systemic therapy (for example antibiotics, antivirals, antifungals).<br><br>18. Has a history or current evidence of any condition, therapy, or laboratory abnormality that might confound the results of the trial, interfere

DocuSign Envelope ID: F89E9DEB-F629-4294-B1D8-B570A4FACE07

	with the patient's participation for the full duration of the trial, or is not in the best interest of the patient to participate, in the opinion of the treating investigator.<br><br>19. Has known psychiatric or substance abuse disorders that would interfere with cooperation with the requirements of the trial.<br><br>20. Pregnant or breastfeeding.<br><br>21. Has received a live vaccine within 30 days prior to the planned start of trial treatment.<br><br>22. Has been hospitalised for bowel obstruction within 4 weeks prior to registration.<br><br>23. Current abdominal/pelvic fistulation.
**Number of sites:**	5 UK sites
**Treatment summary:**	Pembrolizumab 200mg i.v every 3 weeks (Q3W). Patients will receive treatment initially for a maximum of two years, until progression, unacceptable toxicity, clinical decision or patient's withdrawal of consent.
**Duration of recruitment:**	24 months
**Duration of patient follow up:**	Following the end of treatment safety visit (either initial or re-treatment stage, where applicable) data will be collected until the end of study
**Definition of end of trial:**	The end of the trial will be when the last patient alive who has completed initial and re-treatment (if applicable) has two follow-up/survival follow-up assessments (either prior to or after progression) or dies, whichever occurs first and once all protocol planned exploratory research has been completed.

1.2 Trial Schema

Advanced **clear cell gynaecological cancer** patients
(including primary peritoneal and fallopian tube, endometrial, vaginal, vulval or cervical cancer):

- Measurable disease (RECIST v1.1)
- Progressed following ≥ 1 line of chemotherapy
- ECOG 0 or 1
- Patient willing to provide tissue and archival **biopsies**

↓

Eligible and consented patients registered to trial
(N=48)

↓

Pembrolizumab max 2 years
*i.v, 3 weekly cycles, until:

- Disease progression (RECIST v1.1)
- Unacceptable adverse events
- Other clinical / patient decision to stop

Re-treatment *if* applicable
(see section 9.3.3):

- Patient re-consent
- Eligibility
- Registration
- Treatment * - 1 year max

Exploratory Research (initial and re-treatment):
Tumour & Peripheral Blood Biomarkers (response prediction)
Compare with archival specimen

- Baseline: Fresh tumour (biopsy), whole blood, PBMC and plasma
- On treatment: Fresh tumour (biopsy at 6-8 weeks), PBMC and plasma
- Progression: Fresh tumour (biopsy), PBMC and plasma

DocuSign Envelope ID: F89E9DEB-F629-4294-B1D8-B570A4FACE07

2. INTRODUCTION

2.1. Background

2.1.1. Clear cell gynaecological malignancies

Advanced clear cell gynaecological malignancies are associated with poor prognosis and limited response to standard platinum based chemotherapy [1-3]. Whilst clear cell ovarian cancers (including fallopian tube and primary peritoneal cancers, CCOC) are the most common, clear cell tumours of the endometrium (CCEC), vagina, vulva and cervix also occur [1, 2, 4]. There is now compelling evidence to suggest that the different histological subtypes of gynaecological malignancies represent distinct entities with different epidemiology, molecular characteristics, response to treatment, and ultimately outcome [1, 2, 4, 5]. For example, gene expression profiles from CCOC and CCEC show greater similarity with each other, than they do with other site-specific histological subtypes [6]. Furthermore, whilst primary vaginal and vulval clear cell adenocarcinomas are extremely rare and not well studied, anecdotal case reports demonstrate chromosomal and genetic aberrations in a similar pattern to that seen in CCOC [7]. Therefore, it is hypothesized that the distinct molecular profile of clear cell gynaecological cancers supports treating them as a distinct histological identity rather than in a traditional site-specific manner [1, 4, 8, 9].

CCOC represent up to 15% of all epithelial ovarian cancers (EOC) [10] although there is considerable geographic variation with higher incidence in the Far East, Thailand and Spain [11]. In the setting of advanced CCOC, studies have consistently demonstrated a significantly poorer prognosis than other subtypes [10, 12, 13]. CCOC are relatively resistant to conventional platinum-based chemotherapy when compared with other EOC subtypes, with response rates to first-line carboplatin-paclitaxel chemotherapy only 25% (compared to > 70% for high-grade serous carcinoma) [14] and response rates to second line chemotherapy as low as 0-8% [15, 16]. CCEC accounts for 1-6% of endometrial cancers, but represents a difficult to treat subgroup, which are relatively resistant to radiotherapy or chemotherapy and have poorer survival than more common subtypes [1]. As a consequence, clear cell gynaecological malignancies represent a disease subtype where there is significant unmet need and efforts to identify effective novel therapeutic approaches are urgently required.

2.1.2. Pharmaceutical and Therapeutic Background

The importance of intact immune surveillance in controlling outgrowth of neoplastic transformation has been known for decades. Accumulating evidence shows a correlation between tumour-infiltrating lymphocytes (TILs) in cancer tissue and favorable prognosis in various malignancies. In particular, the presence of CD8+ T-cells and the ratio of CD8+ effector T-cells / FoxP3+ regulatory T-cells seems to correlate with improved prognosis and long-term survival in many solid tumours.

The PD-1 receptor-ligand interaction is a major pathway hijacked by tumours to suppress immune control. The normal function of PD-1, expressed on the cell surface of activated

DocuSign Envelope ID: F89E9DEB-F629-4294-B1D8-B570A4FACE07

T-cells under healthy conditions, is to down-modulate unwanted or excessive immune responses, including autoimmune reactions. PD-1 (encoded by the gene Pdcd1) is an Ig superfamily member related to CD28 and CTLA-4 which has been shown to negatively regulate antigen receptor signaling upon engagement of its ligands (PD-L1 and/or PD-L2). The structure of murine PD-1 has been resolved. PD-1 and family members are type I transmembrane glycoproteins containing an Ig Variable-type (V-type) domain responsible for ligand binding and a cytoplasmic tail which is responsible for the binding of signaling molecules. The cytoplasmic tail of PD-1 contains 2 tyrosine-based signaling motifs, an immunoreceptor tyrosine-based inhibition motif (ITIM) and an immunoreceptor tyrosine-based switch motif (ITSM). Following T-cell stimulation, PD-1 recruits the tyrosine phosphatases SHP-1 and SHP-2 to the ITSM motif within its cytoplasmic tail, leading to the dephosphorylation of effector molecules such as CD3ζ, PKCθ and ZAP70 which are involved in the CD3 T-cell signaling cascade. The mechanism by which PD-1 down modulates T-cell responses is similar to, but distinct from that of CTLA-4 as both molecules regulate an overlapping set of signaling proteins. PD-1 was shown to be expressed on activated lymphocytes including peripheral CD4+ and CD8+ T-cells, B-cells, T regs and Natural Killer cells. Expression has also been shown during thymic development on CD4-CD8- (double negative) T-cells as well as subsets of macrophages and dendritic cells. The ligands for PD-1 (PD-L1 and PD-L2) are constitutively expressed or can be induced in a variety of cell types, including non-hematopoietic tissues as well as in various tumours. Both ligands are type I transmembrane receptors containing both IgV- and IgC-like domains in the extracellular region and contain short cytoplasmic regions with no known signaling motifs. Binding of either PD-1 ligand to PD-1 inhibits T-cell activation triggered through the T-cell receptor. PD-L1 is expressed at low levels on various non-hematopoietic tissues, most notably on vascular endothelium, whereas PD-L2 protein is only detectably expressed on antigen-presenting cells found in lymphoid tissue or chronic inflammatory environments. PD-L2 is thought to control immune T-cell activation in lymphoid organs, whereas PD-L1 serves to dampen unwarranted T-cell function in peripheral tissues. Although healthy organs express little (if any) PD-L1, a variety of cancers were demonstrated to express abundant levels of this T-cell inhibitor. PD-1 has been suggested to regulate tumour-specific T-cell expansion in patients with melanoma. This suggests that the PD-1/PD-L1 pathway plays a critical role in tumour immune evasion and should be considered as an attractive target for therapeutic intervention.

Pembrolizumab is a potent and highly selective humanized monoclonal antibody (mAb) of the IgG4/kappa isotype designed to directly block the interaction between PD-1 and its ligands, PD-L1 and PD-L2. Keytruda™ (pembrolizumab) has been approved in the United States and the European Union for the treatment of a number of types of solid tumour (eg PD-L1 positive cervical cancer, melanoma, lung, head and neck, Hodgkin's Lymphoma) including those known to be microsatellite instability high. Phase II data suggests that PDL1 tissue expression may be a predictive marker for response to pembrolizumab in ovarian cancer [30]

DocuSign Envelope ID: F89E9DEB-F629-4294-B1D8-B570A4FACE07

3. RATIONALE

3.1.1. Rationale for the Trial and Selected Patient Population

Many human cancers including ovarian cancer express PD-L1 and have the ability to escape the host immune system via PD-1/PD-L1 signaling and the expression of PD-L1 in ovarian tumours is associated with poor prognosis and inversely correlates with survival [17-19]. PD-L1 expression promotes the progression of ovarian cancer by inducing host immunosuppression of peripheral cytotoxic CD8 Tcell lymphocytes [20] and in vitro data suggests that treatment with anti-PD-1 or anti-PD-L1 antibodies results in tumour rejection in mice [21]. Ovarian and endometrial cancer cells, including CCOC and CCEC express PD-L1 [22, 23] with frequent associated PD-1 positive TILs [23, 24] supporting the hypothesis that targeting PD-1 is a therapeutic option in clear cell gynaecological cancers. The mutational load of tumours correlates with response to immunotherapy [25] and gynaecological malignancies have relatively high mutational loads [26], further supporting the use of immunotherapy in these tumour types. Increasingly, it is being recognized that ‘inflammatory’ cancers have an increased response to immune therapy [27] and upregulation of IL6 and pro-inflammatory cytokine signaling are prominent in CCOC and specific to this subtype of ovarian cancer [28, 29]. Furthermore as the gene expression profiles of CCOC and CCEC are similar to that of clear cell renal cancer [6], the significant improvements in overall survival and response rates with nivolumab noted in renal cancer [30] suggest that anti-PD-1/PD-L1 therapy should be explored in clear cell gynaecological malignancies. Finally, recent data has emerged demonstrating a partial clinical response in two (of two) platinum-resistant patients with CCOC treated the anti-PD-L1, avelumuab [31] and a complete response in one (of two) platinum-resistant CCOC patient treated with the anti-PD-1 nivolumab [32].

The significant unmet clinical need in patients with advanced clear cell gynaecological malignancies together with the pre-clinical data and early clinical responses observed with anti-PD1/PDL1 therapy suggests that pembrolizumab should be trialed in this patient sub-group. This study will assess the objective response rate of pembrolizumab in patients with advanced clear cell gynaecological cancers who have previously received at least one line of prior chemotherapy.

3.1.2. Rationale for Dose Selection/Regimen/Modification

An open-label Phase I trial (Protocol 001) was conducted to evaluate the safety and clinical activity of single agent MK-3475 (pembrolizumab). The dose escalation portion of this trial evaluated three dose levels, 1 mg/kg, 3 mg/kg, and 10 mg/kg, administered every 2 weeks (Q2W) in patients with advanced solid tumours. All three dose levels were well tolerated and no dose-limiting toxicities were observed. This first in human study of MK-3475 showed evidence of target engagement and objective evidence of tumour size reduction at all dose levels (1 mg/kg, 3 mg/kg and 10 mg/kg Q2W). No MTD has been identified to date. Recent data from other clinical studies within the MK-3475 program showed that a lower dose of MK-3475 and a less frequent schedule may be sufficient for target engagement and clinical activity.

DocuSign Envelope ID: F89E9DEB-F629-4294-B1D8-B570A4FACE07

PK data analysis of MK-3475 administered Q2W and Q3W showed slow systemic clearance, limited volume of distribution, and a long half-life (refer to IB). Pharmacodynamic data (IL-2 release assay) suggested that peripheral target engagement is durable (>21 days). This early PK and pharmacodynamic data provides scientific rationale for testing a Q2W and Q3W dosing schedule.

A population pharmacokinetic analysis has been performed using serum concentration time data from 476 patients. Within the resulting population PK model, clearance and volume parameters of MK-3475 were found to be dependent on body weight. The relationship between clearance and body weight, with an allometric exponent of 0.59, is within the range observed for other antibodies and would support both body weight normalized dosing or a fixed dose across all body weights. MK-3475 has been found to have a wide therapeutic range based on the melanoma indication. The differences in exposure for a 200 mg fixed dose regimen relative to a 2 mg/kg Q3W body weight based regimen are anticipated to remain well within the established exposure margins of 0.5 – 5.0 for MK-3475 in the melanoma indication. The exposure margins are based on the notion of similar efficacy and safety in melanoma at 10 mg/kg Q3W vs. the proposed dose regimen of 2 mg/kg Q3W (i.e. 5-fold higher dose and exposure). The population PK evaluation revealed that there was no significant impact of tumour burden on exposure. In addition, exposure was similar between the NSCLC and melanoma indications. Therefore, there are no anticipated changes in exposure between different indication settings.

The rationale for further exploration of 2 mg/kg and comparable doses of pembrolizumab in solid tumors is based on: 1) similar efficacy and safety of pembrolizumab when dosed at either 2 mg/kg or 10 mg/kg Q3W in melanoma patients, 2) the flat exposure-response relationships of pembrolizumab for both efficacy and safety in the dose ranges of 2 mg/kg Q3W to 10 mg/kg Q3W, 3) the lack of effect of tumor burden or indication on distribution behavior of pembrolizumab (as assessed by the population PK model) and 4) the assumption that the dynamics of pembrolizumab target engagement will not vary meaningfully with tumor type.

The choice of the 200 mg Q3W as an appropriate dose for the switch to fixed dosing is based on simulations performed using the population PK model of pembrolizumab showing that the fixed dose of 200 mg every 3 weeks will provide exposures that 1) are optimally consistent with those obtained with the 2 mg/kg dose every 3 weeks, 2) will maintain individual patient exposures in the exposure range established in melanoma as associated with maximal efficacy response and 3) will maintain individual patients exposure in the exposure range established in melanoma that are well tolerated and safe.

DocuSign Envelope ID: F89E9DEB-F629-4294-B1D8-B570A4FACE07

A fixed dose regimen will simplify the dosing regimen to be more convenient for physicians and to reduce potential for dosing errors. A fixed dosing scheme will also reduce complexity in the logistical chain at treatment facilities and reduce wastage.

3.1.3. Rationale for endpoints

3.1.3.1. Efficacy Endpoints

3.1.3.1.1. Primary

The primary endpoint is progression free survival (PFS) rate at 12 weeks from start of treatment according to RECIST v1.1. This population of patients has very poor PFS in the second line setting, therefore an improvement in this endpoint is meaningful. PFS rate also captures patients that have stable disease, which may be the earliest indication of benefit with an immunotherapeutic agent.

3.1.3.1.2. Secondary

The secondary endpoints for this study include objective response rate (ORR) at 12 weeks, best ORR, duration of response (DOR), PFS, time to second progression according to RECIST v1.1 in patients re-treated on trial, Overall Survival (OS), QoL and safety (adverse events according to NCI CTCAE v5). PFS and OS are considered the ‘gold standard’ for quantifying clinical benefit.

3.1.3.1.3. Exploratory

The exploratory endpoints include biomarker assessments.

The purpose of the exploratory biomarker assessments is to identify markers predictive of clinical response and to characterize the pharmacodynamic effects of treatment (i.e., determine if changes in immune status associated with treatment or clinical response) with pembrolizumab.

Collection of tumour biopsy specimens (archival and fresh), as well as whole blood, peripheral blood mononuclear cells (PBMC), and plasma specimens is needed to assess the requested pharmacodynamic and predictive markers. Tumour biopsy specimens provide critical information about the status of the antitumour immune response, at the site of disease. Since the antitumour immune response can evolve with time and in response to different therapies, fresh biopsies are required at screening and during treatment, when clinically feasible. The pre-treatment and post-treatment tissue will be compared to assess changes in the tumour microenvironment after approximately 6 -8 weeks on treatment, at a time when active tumour regression may be occurring in patients receiving pembrolizumab treatment. The fresh biopsy tissue will be compared to archival specimens to characterise molecular evolution over time in clear cell gynaecological cancer.

Archival and fresh tumour biopsy evaluation will include investigating the association between response and the following:

DocuSign Envelope ID: F89E9DEB-F629-4294-B1D8-B570A4FACE07

- PD-1, PD-L1 expression and mutational load in tumour
- Characterization of tumour infiltrating lymphocytes (TILs) and assess cytolytic activity
- ARID1A and PIK3CA tumour mutation status
- HRD status (including BRCA)
- MDM2/MDM4, EGFR tumour mutation status
- RNA sequencing

Biomarker endpoints from peripheral specimens (whole blood, PBMC, and plasma) will be measured at multiple time points (see Section 10 and Appendix 1), and evaluated as both predictive and pharmacodynamic markers in the context of the exploratory biomarker objectives. This will include:

- Immunophenotyping of circulating T cell subsets (naïve, effector, central memory and regulatory), proliferation and activation markers
- Cytokines and chemokines, with an emphasis on IL-6 pathway - indirectly via serum CRP and directly via phospho-STAT3 on IHC and IL6 transcription by qRTPCR
- Other immune-modulatory and checkpoint biomarkers (will include PD-1/PD-L1, 4-1BB, ICOS & LAG3)
- Quantification and analysis of circulating free DNA
- Germline BRCA status, mismatch repair (MMR) status
- CA 125 levels.

DocuSign Envelope ID: F89E9DEB-F629-4294-B1D8-B570A4FACE07

4. TRIAL DESIGN

- This is a multi-centre, single arm, phase II trial in patients with advanced clear cell gynaecological cancer who have received at least one line of chemotherapy.
- Patients will receive pembrolizumab 200 mg intravenously every 3 weeks (Q3W) with a re-treatment option for those who respond as described below*.
- Using a single stage A'Hern design, 48 patients will be enrolled.
- Patients will have a fresh tumour biopsy at baseline, 6-8 weeks after start of treatment and on disease progression (if consent obtained).
- Archival tumour will also be obtained

The primary objective of this study is to determine the PFS rate at 12 weeks with pembrolizumab in patients with advanced clear cell gynaecological malignancies who have received at least one prior line of chemotherapy. The trial will be considered positive (i.e. the null hypothesis will be rejected) if 12 or more patients are progression-free (i.e. have partial or complete response or are stable) at 12 weeks.

All imaging obtained on study will be assessed using RECIST v1.1 for determination of PFS and ORR endpoints.

Adverse events will be monitored throughout the trial and graded in severity according to the guidelines outlined in the NCI CTCAE version 5.

The initial phase of trial treatment with pembrolizumab will continue until any of the following have occurred:

- two years of initial phase of study treatment has been administered
- documented and confirmed disease progression by RECIST v1.1 criteria
- unacceptable adverse event(s)
- intercurrent illness that prevents further administration of treatment
- investigator's decision to withdraw the patient
- patient withdraws consent
- pregnancy of the patient
- non-compliance with trial treatment or procedure requirements

*In patients who attain a confirmed complete response (CR) (i.e. a second scan confirming the presence of a CR according to RECIST criteria v1.1) during the study, the treating clinician may consider stopping trial treatment. These patients will be eligible for re-treatment with pembrolizumab for up to one year after they have experienced radiographic disease progression at the discretion of the investigator as well as in accordance with the criteria in section 9.3.2. This re-treatment will be considered the Second Course Period (Re-treatment) .

Patients with Stable Disease (SD), Partial Response (PR) or CR who completed 24 months of pembrolizumab treatment are also eligible for re-treatment (Section 9.3.2) at the time of radiographic disease progression and at the discretion of the investigator.

DocuSign Envelope ID: F89E9DEB-F629-4294-B1D8-B570A4FACE07

Response or progression in the re-treatment period will not be considered part of the primary endpoint in this trial, time to second progression for these patients is included in the secondary endpoints.

4.1. Trial Objectives

4.1.1. Primary

To assess the efficacy of pembrolizumab in patients with advanced clear cell gynaecological cancer.

4.1.2. Secondary

- To assess whether pembrolizumab prolongs progression-free survival and/or overall survival when used in this setting.
- To assess the effect of pembrolizumab on the objective response rate at 12 weeks, and best objective response rate over the entire study and within the re-treatment phase.
- To assess what the duration of response is when patients with advanced clear cell gynaecological cancer are treated with pembrolizumab over the entire study and within the re-treatment phase.
- To assess safety.
- To assess quality of life during treatment with pembrolizumab.
- To assess the time to second progression in patients re-treated with pembrolizumab.

4.1.3. Exploratory Objectives

- To evaluate potential tumour and peripheral blood biomarkers.
- To assess whether clinical outcomes vary by immune response.

4.2. Trial Endpoints

4.2.1. Primary

PFS rate at 12 weeks according to RECIST v1.1 criteria.

4.2.2. Secondary

- Progression-free survival.
- Overall survival.

DocuSign Envelope ID: F89E9DEB-F629-4294-B1D8-B570A4FACE07

- Objective response rate at 12 weeks, and best objective response rate over the entire study and within the re-treatment phase.
- Duration of response over the entire study and within the re-treatment phase.
- Assessment of adverse events.
- QoL – FACT-O.
- Time to second progression in patients re-treated with pembrolizumab.

4.2.3. Exploratory

1. To determine pharmacodynamics of mechanistic and predictive biomarkers in archival, pre-treatment, during treatment and post-treatment progression tumour biopsies and circulating biomarkers according to emerging data, including:

    - PD-1, PD-L1 expression and mutational load and association with response
    - ARID1A, MMR, BRCA and PIK3CA mutation status and association with response
    - HRD status (including BRCA)
    - characterization of tumour infiltrating lymphocytes (TILs) as well as cytolytic activity and association with outcome
    - MDM2/MDM4, EGFR tumour mutation status.
    - RNA sequencing

2. Evaluate peripheral blood biomarkers and association with clinical outcomes, including:
    - immunophenotype circulating T cell subsets (naïve, effector, central memory and regulatory), proliferation, and activation markers
    - cytokines and chemokines, with an emphasis on IL-6 pathway - indirectly via serum CRP and directly via phospho-STAT3 on IHC and IL6 transcription by qRTPCR,
    - other immune-modulatory and checkpoint biomarkers (will include PD-1/PD-L1, 4-1BB, ICOS, LAG3, CA 125 and BRCA)
    - quantification and analysis of circulating free DNA.

4.3. Trial Activation

UCL CTC will ensure that all trial documentation has been reviewed and approved by all relevant bodies and that the following have been obtained prior to activating the trial:

- Health Research Authority (HRA) approval, including Research Ethics Committee approval
- Clinical Trial Authorisation from the Medicines and Healthcare products Regulatory Agency (MHRA)
- **‘**Adoption’ onto the NIHR portfolio
- Adequate funding for central coordination
- Confirmation of sponsorship
- Adequate insurance provision

DocuSign Envelope ID: F89E9DEB-F629-4294-B1D8-B570A4FACE07

5. SELECTION OF SITES/SITE INVESTIGATORS

5.1. Site Selection

In this protocol trial 'site' refers to a hospital where trial-related activities are conducted.

Sites must be able to comply with:

- Trial treatment, imaging, clinical care, follow up schedules and all requirements of the trial protocol.
- Requirements of the UK Policy Framework for Health and Social Care Research, issued by the Health Research Authority, and the Medicines for Human Use Clinical Trials) Regulation (SI 2004/1031), and all amendments.
- Data collection requirements, including adherence to eCRF completion timelines as per section 12.3 (Timelines for Data).
- Biological sample collection, processing and storage requirements.
- Monitoring requirements, as outlined in protocol section 15 (Trial Monitoring and Oversight) and trial monitoring plan.

5.1.1. Selection of Principal Investigator and other investigators at sites

Sites must appoint an appropriate Principal Investigator (PI), i.e. a healthcare professional authorised by the site to lead and coordinate the work of the trial on behalf of the site. Co-investigators must be trained and approved by the PI. All investigators must be medical doctors and have experience of treating gynaecological cancers. The PI is responsible for the conduct of the trial at their site and for ensuring that any amendments are implemented in a timely fashion. If a PI leaves/goes on a leave of absence, UCL CTC **must be informed promptly** and a new PI identified and appointed by the site.

5.1.2. Training requirements for site staff

All site staff must be appropriately qualified by education, training and experience to perform the trial related duties allocated to them, which must be recorded on the site delegation log.

CVs for all staff must be kept up-to-date, signed and dated and copies held in the Investigator Site File (ISF). A current, signed copy of the CV with evidence of GCP training (or copy of GCP certificate) for the PI must be forwarded to UCL CTC upon request.

GCP training is required for all staff responsible for trial activities. The frequency of repeat training may be dictated by the requirements of their employing institution, or 2 yearly where the institution has no policy, and more frequently when there have been updates to the legal or regulatory requirements for the conduct of clinical trials.

DocuSign Envelope ID: F89E9DEB-F629-4294-B1D8-B570A4FACE07

5.2. Site initiation and Activation

5.2.1. Site initiation

Before a site is activated, the UCL CTC trial team will arrange a site initiation with the site which the PI, the pharmacy lead and relevant members of site research team should attend. It is recommended that the site's specialist gynaecological oncology pathologist(s) should also attend / or has been made aware of the trial requirement for reviewing the histology samples for the trial. The site will be trained in the day-to-day management of the trial and essential documentation required for the trial will be checked.

Site initiation will be performed for each site by site visit. Re-initiating sites may be required where there has been a significant delay between initiation and enrolling the first patient, as per monitoring plan.

5.2.2. Required documentation

The following documentation must be submitted by the site to UCL CTC prior to a site being activated by the UCL CTC trial team:

- Trial specific Site Registration Form (identifying relevant local staff).
- Relevant institutional approvals / confirmation of capacity and capability.
- A completed site delegation log that is initialled and dated by the PI (with <u>all</u> tasks and responsibilities delegated appropriately).
- Completed site contacts form (with contact information for all members of local staff).
- A signed and dated copy of the PI's current CV (with documented up-to-date GCP training, or copy of GCP training certificate).
- Trial specific prescription.

In addition, the following agreements must be in place:

- A signed Site Agreement between the Sponsor and the relevant institution (usually an NHS Trust/Health Board).

5.2.3. Site activation letter

Once the UCL CTC trial team has received all required documentation and the site has been initiated, a site activation letter will be issued to the PI, research team and pharmacy, at which point the site may start to approach patients.

Following site activation, the PI is responsible for ensuring:

- Adherence to the most recent version of the protocol.
- All relevant site staff are trained in the protocol requirements.
- Appropriate recruitment and medical care of patients in the trial.

DocuSign Envelope ID: F89E9DEB-F629-4294-B1D8-B570A4FACE07

- Timely completion of eCRFs (including assessment of all adverse events) and response to data queries.
- Prompt notification and assessment of all serious adverse events and AEs of special interest.
- That the site has facilities to provide **24 hour medical advice** for trial patients.

DocuSign Envelope ID: F89E9DEB-F629-4294-B1D8-B570A4FACE07

6. INFORMED CONSENT

Sites are responsible for assessing a patient's capacity to give informed consent.

Sites must ensure that all patients have been given the current approved version of the patient information sheets, are fully informed about the trial and have confirmed their willingness to take part in the trial by signing the current approved consent form.

Sites must assess a patient's ability to understand verbal and written information in English and whether or not an interpreter would be required to ensure fully informed consent. If a patient requires an interpreter and none is available, the patient should not be considered for the trial.

The PI, or, where delegated by the PI, other appropriately trained site staff, are required to provide a full explanation of the trial and all relevant treatment options to each patient prior to trial entry. During these discussions, the current approved patient information sheets for the trial should be discussed with the patient.

A **minimum of twenty four (24) hours** must be allowed for the patient to consider and discuss participation in the trial.

Written informed consent on the current approved version of the consent forms for the trial must be obtained before any trial-specific procedures are conducted. The discussion and consent process must be documented in the patient notes.

Site staff are responsible for:

- checking that the current approved versions of the patient information sheets and consent forms are used
- checking that information on the consent forms are complete and legible
- checking that the patient has initialled <u>all</u> relevant sections and signed and dated the form
- checking that an appropriate member of staff has countersigned and dated the consent forms to confirm that they provided information to the patient
- checking that an appropriate member of staff has made dated entries in the patient's medical notes relating to the informed consent process (i.e. information given, consent signed etc.)
- following initial registration, adding the patient's trial number to the consent form, which should be filed in the patient's medical notes and investigator site file.
- following initial registration giving the patient a copy of their signed consent form, patient information sheet and patient contact card.

  if applicable, following registration for re-treatment with IMP on trial (see section 9.3.3 'Re-treatment Period'), giving a copy of the second signed consent form to the patient, which should also be filed in the patient's medical notes and investigator site file.

DocuSign Envelope ID: F89E9DEB-F629-4294-B1D8-B570A4FACE07

- The right of the patient to refuse to participate in the trial without giving reasons must be respected. All patients are free to withdraw at any time (refer to section 16) (Withdrawal of Patients).

Where re-consent is required, sites will need to document the re-consent details on the trial specific re-consent log, which can be found in the ISF. The below process should be followed for re-consenting patients in follow-up (where required) who have progressed and are no longer required to return to the hospital for the trial i.e.:

- Research teams to discuss the changes in the PIS with the patient over the phone.
- Post the PIS to the patient and ICF to sign and ask the patient to post back to the research team.
- Once the signed ICF is returned, the delegated research clinician can countersign.
- Site should then post back a fully signed copy to the patient.

Site record the above re-consenting process for these patients in their medical notes and complete the re-consent log annotating for which patients this process was applied.

7. SELECTION OF PATIENTS

7.1. Screening Log

A screening log must be maintained and appropriately filed at site. Sites should record each patient screened for the trial and the reasons why they were not registered in the trial if this is the case. The log must be sent to UCL CTC when requested.

Sites should include the following potential patients on the screening log, who have:

- histological confirmation of gynaecological cancer including ovarian (including primary peritoneal and fallopian tube), endometrial, vaginal, vulval or cervical cancer,
- relapsed after at least one line of chemotherapy,
- advanced disease measurable according to RECIST v1.1.

7.2. Patient Eligibility

There will be no exception to the eligibility requirements at the time of registration. Queries in relation to the eligibility criteria must be addressed prior to registration. Patients are eligible for the trial if all the inclusion criteria are met and none of the exclusion criteria applies.

Patients' eligibility must be confirmed by an investigator who is suitably qualified and who has been allocated this duty, as documented on the site staff delegation log, prior to registering the patient. Confirmation of eligibility **must be documented in the patients' notes** and on the registration eCRF.

Patients must give written informed consent before any trial specific screening investigations may be carried out. See section 8.1.1 for the list of assessments and procedures required to evaluate the suitability of patients prior to entry.

7.2.1. Inclusion criteria

In order to be eligible for participation in this trial, the patient must:

1. Have a histological diagnosis of advanced clear cell cancer from the gynaecological tract: ovarian (including primary peritoneal and fallopian tube), endometrial, vaginal, vulval or cervical cancer. Tumour specimens must be at least 50% clear cell histology as determined by the named specialist gynaecological histopathologist(s) reviewing this on behalf of the trial site.
2. Be willing and able to provide written informed consent for the trial.
3. Be ≥ 18 years of age on day of signing initial registration informed consent.

DocuSign Envelope ID: F89E9DEB-F629-4294-B1D8-B570A4FACE07

4. Patient must have at least one measurable lesion according to RECIST v1.1 in addition to a separate biopsiable lesion. Measurable lesions should be outside any prior radiation field unless progression has occurred at that site.

5. Evidence of radiological disease progression.

6. Patient is willing to provide tissue from a newly obtained core or excisional biopsy of a tumour lesion at baseline, 6-8 weeks after start of treatment and at the time of progression **(patients are not eligible if the cancer cannot be safely biopsied at baseline).**

7. Patient is willing to provide archival tissue.

8. Have an ECOG performance status of 0 or 1.

9. Have a life expectancy of at least 4 months from consent.

10. Received ≥ 1 line of prior chemotherapy (including the adjuvant setting) for clear cell gynaecological cancer.

11. Demonstrate adequate organ function as defined in Table 1. All screening blood tests should be performed within 10 days prior to registration, unless otherwise specified in the table.

Table 1: Adequate Organ Function Laboratory Values

System	Laboratory Value
**Haematological**	
Absolute neutrophil count (ANC)	≥1.5x 10^9/l
Platelets	≥100x 10^9/l
Haemoglobin	≥90 g/L or ≥5.6 mmol/L without transfusion within 14 days or EPO dependency. Patients cannot have had a blood transfusion within 14 days of planned day 1 of treatment
**Renal**	
Serum creatinine **OR** Measured or calculated[a] creatinine clearance (GFR can also be used in place of creatinine or CrCl)	≤1.5 X upper limit of normal (ULN) **OR**<br><br>≥60 mL/min for patient with creatinine levels > 1.5 X institutional ULN
**Hepatic**	
Serum total bilirubin	≤ 1.5 X ULN **OR**
	Direct bilirubin ≤ ULN for patients with total bilirubin levels > 1.5 X ULN
AST (SGOT) and ALT (SGPT)	≤1.5 X ULN **OR** ≤ 2.5 X ULN if liver metastases present
Albumin	>2.5 g/dL or >25 g/L
**Coagulation**	
International Normalized Ratio (INR)	≤1.5

**Bone Profile**	
Serum calcium	<2.9 mmol/L
[a]Creatinine clearance should be calculated per institutional standard.	

12. For women of childbearing potential, negative urine pregnancy test within 72 hours prior to receiving the first dose of study medication. If the urine test is positive or cannot be confirmed as negative, a serum pregnancy test will be required.

13. Women of childbearing potential as defined in Section 7.3.1 must be willing to use a highly effective method of contraception as outlined in Section 7.3.4 for the course of the study through 120 days after the last dose of study medication.

Note: Abstinence is acceptable if this is the usual lifestyle and preferred birth control method for the patient for the duration required on trial.

7.2.2. Exclusion criteria

The patient must be excluded from participating in the trial if the patient:

1. Is currently participating and receiving other study therapy or has participated in a study of an investigational agent and received study therapy or used an investigational device within 4 weeks of the first dose of treatment.

2. Has had a prior anti-cancer monoclonal antibody (mAb), chemotherapy or targeted small molecule therapy within 4 weeks prior to study Day 1 or who has not recovered (i.e. ≤ Grade 1 or at baseline) from side effects due to a previously administered agent.

   Note:
   - Patients with ≤ Grade 2 neuropathy and Grade 2 alopecia are an exception to this criterion and may qualify for the study.
   - If patient received any major surgery, they must have recovered adequately from the event and/or complications from the intervention prior to starting therapy.

3. Has had prior radiation therapy within 2 weeks prior to day 1 of planned study treatment.

4. Has received prior therapy with an anti-PD-1, anti-PD-L1, or anti-PD-L2 agent.

5. Has a diagnosis of immunodeficiency or is receiving systemic steroid therapy at doses > 10mg prednisolone daily or equivalent or any other form of immunosuppressive therapy within 7 days prior to the first dose of trial treatment.

DocuSign Envelope ID: F89E9DEB-F629-4294-B1D8-B570A4FACE07

6. Has a known history of active TB (Bacillus Tuberculosis).

7. Has a known history of Hepatitis B (defined as Hepatitis B surface antigen [HBsAg] reactive) or known active Hepatitis C virus (defined as HCV RNA [qualitative] is detected) infection.

8. Has a known history of Human Immunodeficiency Virus (HIV) (HIV type 1/2 antibodies).

9. Known hypersensitivity to pembrolizumab or any of its excipients.

10. Has a known additional malignancy that is progressing or requires active treatment. If there is a history of a second malignancy and there is doubt regarding the aetiology of progressive disease, then a biopsy is required to determine the diagnosis.

11. Has known active central nervous system (CNS) metastases and/or carcinomatous meningitis. Patients with previously treated brain metastases may participate provided they are stable (no evidence of progression by imaging for at least four weeks prior to the first dose of trial treatment and any neurologic symptoms have returned to baseline), have no evidence of new or enlarging brain metastases, and are not using steroids >10mg prednisolone daily or equivalent for at least 7 days prior to trial treatment. This exception does not include carcinomatous meningitis which is excluded regardless of clinical stability.

12. Has active autoimmune disease that has required systemic treatment in the past 2 years (i.e. with use of disease modifying agents, corticosteroids (at doses > than 10mg prednisolone daily or equivalent) or immunosuppressive drugs). Replacement therapy (eg., thyroxine, insulin, or physiologic corticosteroid replacement therapy for adrenal or pituitary insufficiency, etc.) is not considered a form of systemic treatment.

13. Has a corrected serum calcium of > grade 1 hypercaelcemia (>2.9 mmol/L) despite maximal antihypercalcaemic therapy.

14. Has known history of (non-infectious) pneumonitis that required steroids or has current pneumonitis.

15. Has a newly diagnosed venous thromboembolic event (e.g. Pulmonary Embolism, Deep Vein Thrombosis) unless patient has received at least 14 days of therapeutic anticoagulation for a new thromboembolic event and is suitable for continued therapeutic anticoagulation during trial participation.

16. Patients are excluded if they have a history of arterial thrombosis (excluding the pulmonary artery), within 12 months prior to registration (if beyond 12 months prior to registration, patient may be included providing they have fully recovered clinically).

17. Has an active infection requiring systemic therapy (for example antibiotics, antifungals, antivirals).

18. Has a history or current evidence of any condition , therapy, or laboratory abnormality that might confound the results of the trial, interfere with the patient's participation for the full duration of the trial, or is not in the best interest of the patient to participate, in the opinion of the treating investigator.

19. Has known psychiatric or substance abuse disorders that would interfere with cooperation with the requirements of the trial.

20. Is pregnant or breastfeeding, or expecting to conceive within the projected duration of the trial, starting with the pre-screening or screening visit through 120 days after the last dose of trial treatment.

21. Has received a live vaccine within 30 days of planned start of study therapy.

Note: Seasonal influenza vaccines for injection are generally inactivated flu vaccines and are allowed; however intranasal influenza vaccines (e.g., Flu-Mist®) are live attenuated vaccines, and are not allowed.

22. Has been hospitalised for bowel obstruction within 4 weeks prior to registration.

23. Current abdominal/pelvic fistulation.

7.3. Pregnancy and birth control

7.3.1. Pregnancy and birth control

Definition of women of childbearing potential (WOCBP)

A WOCBP is a sexually mature woman (i.e. any female who has experienced menstrual bleeding) who:

- Has not undergone a hysterectomy or bilateral oophorectomy/salpingectomy
- Is not postmenopausal (a post-menopausal woman is a female who has not had menses at any time in the preceding 12 consecutive months without an alternative medical cause)
- Has not had premature ovarian failure confirmed by a specialist gynaecologist

7.3.2. Risk of exposure to trial treatment during pregnancy

The risk of exposure to trial treatment has been evaluated using the safety information available in the IB for pembrolizumab. Pembrolizumab may have adverse effects on a foetus in utero.

DocuSign Envelope ID: F89E9DEB-F629-4294-B1D8-B570A4FACE07

7.3.3. Pregnancy testing

WOCBP must undergo pregnancy testing, on day of registration (or within 72 hours prior if registration day will be the same day as planned start of treatment), prior to every cycle of treatment and at the end of treatment assessment.

7.3.4. Contraceptive Advice

Female patients who are women of childbearing potential must use a highly effective method of birth control from consent, while receiving study drug and for 120 days after the last dose of study drug. Methods of highly effective contraception/birth control are:

- combined (estrogen and progestogen containing) hormonal contraception associated with inhibition of ovulation:
    - oral
    - intravaginal
    - transdermal
- progestogen-only hormonal contraception associated with inhibition of ovulation:
    - oral (e.g. desogestrel)
    - injectable
    - implantable[1]
- intrauterine device (IUD)[1]
- intrauterine hormone-releasing system (IUS)[2]
- bilateral tubal occlusion[1]
- vasectomised partner[1,2]
- sexual abstinence[3]

1. Contraception methods that are considered to have low user dependency.

2. Vasectomised partner is a highly effective birth control method provided that partner is the sole sexual partner of the WOCBP trial participant and that the vasectomised partner has received medical assessment of the surgical success.

3. Sexual abstinence is considered a highly effective method only if defined as refraining from heterosexual intercourse during the entire period of risk associated with the study treatments. The reliability of sexual abstinence needs to be evaluated in relation to the duration of the clinical trial and the preferred and usual lifestyle of the patient.

The method(s) of contraception/birth control used must be stated in the patient medical notes.

7.3.5. Action to be taken in the event of a pregnancy

If a patient becomes pregnant:

- prior to initiating treatment, the patient will not receive trial treatment unless they elect to have a termination (please note, in such instances, termination must be the patient’s own choice)
- while on treatment with pembrolizumab, the patient will immediately be removed from receiving further study treatment.

DocuSign Envelope ID: F89E9DEB-F629-4294-B1D8-B570A4FACE07

- after the end of the treatment, but during the pregnancy at-risk period (120 days after last treatment or 30 days following cessation of treatment if the patient initiates new anticancer therapy, whichever is earlier)

the site must report the pregnancy to UCL CTC and will contact the patient at least monthly and document the patient's status until six weeks after pregnancy outcome , if the patient consents to pregnancy monitoring. The initial notification of the pregnancy, significant updates and the final outcome of the pregnancy will be reported to the Sponsor and to Merck MSD without delay and within 24 hours to the Sponsor UCL CTC (see also section 13.6 for pregnancy reporting).

7.3.6. Long Term Infertility

In light of the identified potential risk related to pharmacologically-mediated inhibition of the PD-1 pathway, no reproductive or developmental toxicity studies were conducted with pembrolizumab. Therefore, inclusion of women of childbearing potential in clinical trials should be in accordance with this study protocol and applicable regulatory guidance.

No clinical data are available on the possible effects of pembrolizumab on fertility. Although reproductive and developmental toxicity studies have not been conducted with pembrolizumab, there were no notable effects in the male and female reproductive organs in monkeys based on 1-month and 6-month repeat dose toxicity studies.

7.3.7. Lactation

It is unknown whether pembrolizumab is excreted in human milk. Since many drugs are excreted in human milk, and because of the potential for serious adverse reactions in the nursing infant, patients who are breast-feeding are not eligible for enrollment.

8. REGISTRATION PROCEDURES

8.1. Registration

Patient registration will be undertaken by site and UCL CTC within 3 days prior to commencement of day 1, cycle 1 of the initial trial treatment.

8.1.1. Pre-registration Assessments (initial treatment)

Patients must give written informed consent **before** any trial specific screening investigations may be carried out and fulfil the eligibility criteria outlined in Section 7.2.

The following assessments or procedures are required to evaluate the suitability of patients for the trial (see also Appendix 1 'Schedule of Procedures/Assessments'). All assessments must be completed within 28 days prior to the patient being registered (unless otherwise indicated):

- **Histological confirmation of diagnosis** and presence of ≥50% clear cell component by named specialist gynaecological histopathologist(s) at trial site (if the named trial site histopathologist(s) are not available, review by one of the named histopathologists at one of the other trial sites may be provided instead).
- **Medical history.**
- **Full physical exam** including height and weight.
- **Vital signs:** heart rate, respiratory rate, blood pressure, temperature and oxygen saturation (O2 sats).
- **Concomitant medication review.**
- **ECOG performance status.**
- **CT scan of chest, assessable by RECIST v1.1.**
- **CT scan or MRI scan(s) assessable by RECIST v1.1** of the pelvis, abdomen **and brain.**
- **Bloods:** Full blood count and liver function tests, urea & electrolytes, International Normalized Ratio (INR), thyroid function tests, serum calcium and urinalysis **(within 10 days** prior to registration).
- **HIV type 1/2, Hepatitis B and C testing.**
- **Urine or serum pregnancy test** (if applicable) (on day of registration or within 72 hours prior to the registration day if this will be same day as planned start of treatment)
- **Archival tissue collection** for diagnosis*.
- **Newly obtained mandatory tissue (biopsy)** – for exploratory research.
- **Ca125 - within 14 days prior to registration.**
- **BRCA status and MMR status**, capture if known.
- **Exploratory research bloods:** whole blood, PBMCs and plasma (please also refer to the lab manual). Note: samples can be taken after registration and before cycle 1, day 1 if more suitable for the site.
- **QoL: FACT-O.**

DocuSign Envelope ID: F89E9DEB-F629-4294-B1D8-B570A4FACE07

*All patients should have available archival tissue samples and these have to be quality reviewed locally to confirm diagnosis prior to registration.

Once the presence of at least 50% clear cell histology is confirmed by the delegated gynaecological specialist pathologist on an archival diagnostic specimen, the patient will have to undergo **3 mandatory biopsies** during the course of the clinical trial. Please refer to the PEACOCC laboratory manual for further details. Note: archival tissues are not sent for central review in real time for eligibility confirmation. If mixed histology, the pathology report for the trial should record that the specimen is at least 50% clear cell histology.

Each block provided should demonstrate at least 50% clear cell histology. If one block demonstrates an alternative histology e all blocks are then required to be reviewed to confirm > 50%.

Following pre-treatment evaluations, confirmation of eligibility and consent of a patient at a site, the registration eligibility checklist and required baseline form(s) must be fully completed and the information entered onto the registration database (MACRO 4 online). UCL CTC will then be notified of the registration data entry and the PEACOCC Trial team will check the eligibility and baseline assessment data fulfil protocol requirements. If further information is required, UCL CTC will contact the site to discuss.

Once eligibility and baseline assessments have been checked, the PEACOCC trial team will confirm the patient's registration and patient's trial number with the site.

UCL CTC will send the e-mail confirmation of the patient's inclusion in the trial to the PI / treating clinician, main research team contact and pharmacy.

Registration queries telephone number:	+44 (0)20 7679 9284
Registration fax number (if required):	+44 (0)20 7679 9871
UCL CTC Registration hours:	09:00 to 17:00 Monday to Friday, excluding Bank Holidays

Once a patient has been registered onto the trial they must be provided with the following:

- A copy of their signed consent form and patient information sheet.
- A patient contact card. Site contact details for 24 hour medical care must be added to this card and patients advised to carry this with them at all times while participating in the trial.
- After registration into the trial, the patient's general practitioner (GP) should be informed of the patient's involvement in the trial by the site completing and sending the completed GP letter.

DocuSign Envelope ID: F89E9DEB-F629-4294-B1D8-B570A4FACE07

8.1.2 Re-treatment Registration

Patients who provide continued consent and meet the eligibility criteria for re-treatment will need to be registered for the trial re-treatment with UCL CTC. The registration process will be the same as described in the previous section i.e. following evaluations, confirmation of eligibility and consent of a patient at a site for re-treatment, the registration eligibility checklist and required re-treatment baseline form(s) must be fully completed and the information entered onto the registration database. A new trial number will not need to be assigned, the patient's trial number will remain the same.

These patients must have their re-treatment eligibility checked as per section 9.3.3.

The following must be carried out before registration for re-treatment:

- Re-treatment pre-registration assessments completed as detailed below in section 8.1.2.1.
- Confirmation of eligibility by the clinician for re-treatment **as per section 9.3.3.**
- Consent for re-treatment as per section 6.
- The re-treatment registration eligibility checklist and baseline forms must be fully completed

UCL CTC will e-mail confirmation of the patient's registration for re-treatment to the PI/treating clinician, main research team contact and pharmacy

8.1.2.1 Re-treatment Pre-registration Assessments

The following assessments or procedures are required to evaluate the suitability of patients for re-treatment (see also Appendix 1 'Schedule of Procedures/Assessments). All assessments must be completed within 28 days prior to the patient being re-registered (unless otherwise indicated).

- **Medical history.**
- **Full physical exam** including weight
- **Vital signs:** heart rate, respiratory rate, blood pressure, temperature and oxygen saturation (O2 sats).
- **Concomitant medication review.**
- **ECOG performance status.**
- **CT scan of chest, assessable by RECIST v1.1.**
- **CT scan or MRI scan(s) assessable by RECIST v1.1** of the pelvis, abdomen **<u>and brain.</u>**
- **Bloods:** Full blood count and liver function tests, urea & electrolytes, International Normalized Ratio (INR), thyroid function tests, serum calcium and urinalysis (within 10 days prior to registration).
- **HIV type 1/2, Hepatitis B and C testing.**

DocuSign Envelope ID: F89E9DEB-F629-4294-B1D8-B570A4FACE07

- **Urine or serum pregnancy test** (if applicable) (on day of registration or within 72 hours prior to the registration day if this will be same day as planned start of re-treatment)
- **BRCA & MMR status** (if known)
- **Tissue (biopsy)** – for translational research
- **Ca125** within **14 days** prior to re-registration
- **Exploratory research bloods:** whole blood, PBMCs and plasma (please refer to the lab manual). Note: samples can be taken after registration and before cycle 1, day 1 if more suitable for the site.
- **QoL: FACT-O**

8.2. Initial Trial Drug Supply

Upon activation an initial drug order will be placed by UCL CTC for study IMP to arrive on site in anticipation of first patient recruited. Please also refer to Summary of Drug Arrangements for full details of initial supply of pembrolizumab for the trial and placing subsequent drug orders.

DocuSign Envelope ID: F89E9DEB-F629-4294-B1D8-B570A4FACE07

9. TRIAL TREATMENT

Investigational Medicinal Products (IMPs)

For the purpose of this protocol, **the trial IMP is Pembrolizumab, also referred to as MK-3475.**

There are no non-investigational medicinal products (NIMPs).

9.1. Investigational Medicinal Products

Pembrolizumab is unlicensed for advanced clear cell gynaecological cancer patients. It is manufactured and supplied by Merck Sharp and Dohme (MSD) for the PEACOCC trial. Licensed indications are as per the pembrolizumab IB supplied to sites for PEACOCC.

Trial IMP, Pembrolizumab, must not be used outside the context of this trial. Under no circumstances should the site investigator or other site personnel supply trial drug to other investigators, patients or clinics, or allow supplies to be used other than directed by this protocol without prior authorisation from the Supplier and notification to the Sponsor. Please refer to the Summary of Drug Arrangements (SoDA) for full arrangements for the trial.

The PI shall take responsibility for and shall take all steps to maintain appropriate records and ensure appropriate supply, storage, handling, distribution and usage of investigational product in accordance with the protocol and any applicable laws and regulations. Record keeping of the IMP can be delegated to the Pharmacy Lead by the PI.

9.1.1. Packaging and Labelling Information

Clinical Supplies will be provided by MSD as summarised in Table 2.

Table 2: IMP description

IMP Name & Potency	Dosage Form
Pembrolizumab / MK-3475 100 mg/ 4mL	Solution for Infusion

Clinical supplies will be affixed with a clinical trial label in accordance with regulatory requirements. There is no automated drug kit/batch allocation, manual dispensing, will be in accordance with site local policy and as per the summary of drug arrangements.

9.1.2. Clinical Supplies Disclosure

This trial is open-label; therefore, the patient, the trial site personnel and the Sponsor are not blinded to treatment. Drug identity (name, strength) is included in the label text; random code/disclosure envelopes or lists are not provided.

DocuSign Envelope ID: F89E9DEB-F629-4294-B1D8-B570A4FACE07

9.2. Treatment Summary

The treatment to be used in this trial is outlined below in Table 3. The rationale for selection of doses to be used in this trial is provided in Section 3.1.2.

Details on preparation and administration of pembrolizumab are provided in the summary of drug arrangements (SoDA).

Table 3: Trial Treatment

Drug	Dose	Dose Frequency	Route of Administration	Treatment Period	Use
Pembrolizumab	200 mg	3 weekly	IV infusion	Day 1 of each 3 weekly cycle	Investigational

Trial treatment should begin as close as possible to the date on which treatment is assigned.

9.3. Trial Treatment Details

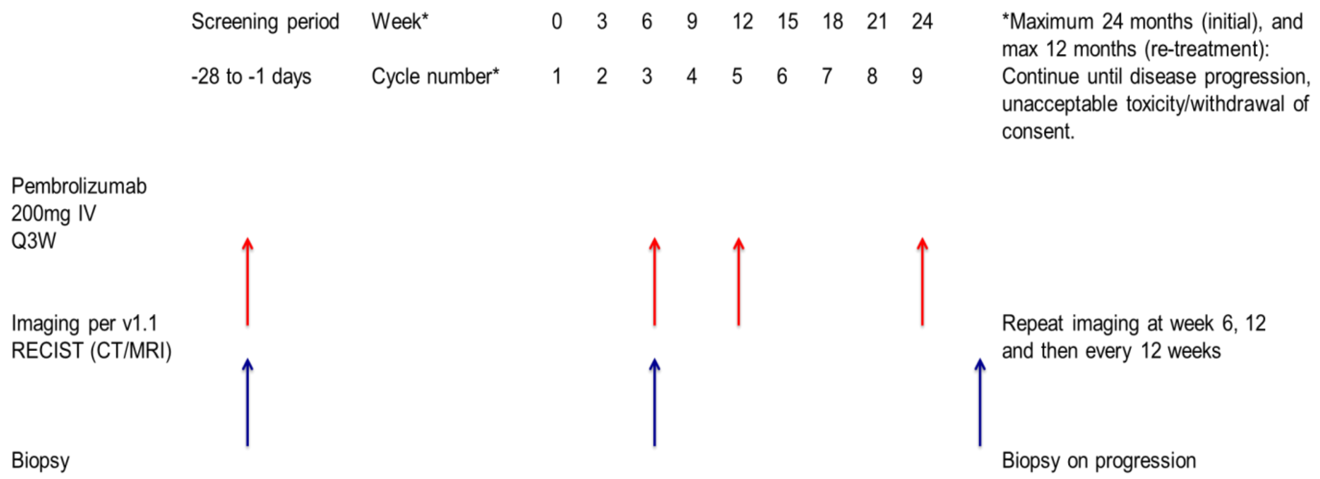

9.3.1. Timing of Dose Administration

Trial treatment should be administered on Day 1 of each 3 weekly cycle after all procedures/assessments have been completed as detailed on the Schedule of Assessments.

(Appendix 1). Trial treatment may be administered up to 3 days before or after the scheduled Day 1 of each cycle due to administrative reasons.

All trial treatments will be administered on an outpatient basis.

Pembrolizumab 200 mg will be administered as a 30 minute IV infusion every 3 weeks. Sites should make every effort to target infusion timing to be as close to 30 minutes as possible. However, given the variability of infusion pumps from site to site, a window of -5 minutes and +10 minutes is permitted (i.e., infusion time is 30 minutes: -5 min/+10 min).

The Summary of Drug Arrangements (SoDA) contains specific instructions for the preparation of the pembrolizumab infusion fluid and administration of infusion solution.

9.3.2. Treatment Duration

The treatment with pembrolizumab will continue for a maximum of two years initially unless any of the following occur:

- documented and confirmed disease progression according to RECIST v1.1
- unacceptable adverse event(s)
- intercurrent illness that prevents further administration of treatment
- investigator’s decision to withdraw the patient
- patient withdraws consent
- pregnancy of the patient
- non-compliance with trial treatment or procedure requirements

9.3.3. Re-treatment Period

Re-treatment as part of this study is only available for a maximum of one year if the patient meets the following conditions:

- Either, the patient
    - Stopped initial treatment with pembrolizumab after attaining an investigator-determined confirmed complete response (CR) according to RECIST v1.1, and
        - Was treated for at least 24 weeks with pembrolizumab before discontinuing therapy
        - Received at least two treatments with pembrolizumab beyond the date when the initial CR was declared

      OR

    - Had SD, PR or CR and stopped pembrolizumab treatment after 24 months of study therapy for reasons other than disease progression or intolerability

DocuSign Envelope ID: F89E9DEB-F629-4294-B1D8-B570A4FACE07

AND

- Experienced a confirmed radiographic disease progression according to RECIST v1.1.after stopping their initial treatment with pembrolizumab

- Did not receive any anti-cancer treatment since the last dose of pembrolizumab

- Has a performance status of 0 or 1 on the ECOG Performance Scale

- Demonstrates adequate organ function as detailed in Section 7.2.1.

- Female patient of childbearing potential should have a negative urine or serum pregnancy test within 72 hours prior to receiving retreatment with study medication.

- Female patient of childbearing potential should be willing to use a highly effective form of contraception as detailed in section 7.3.1.

- Does not have an ongoing history or current evidence of any condition, therapy, or laboratory abnormality that might interfere with the patient's participation for the re-treatment period and full duration of the trial or is not in the best interest of the patient to participate, in the opinion of the treating investigator.

Patients who restart treatment will be retreated at the same dose and dose interval as when they last received pembrolizumab.

Permanent discontinuation of re-treatment is required if any of the following occur:

- documented and confirmed disease progression by RECIST v1.1 criteria
- unacceptable adverse event(s)
- intercurrent illness that prevents further administration of treatment
- investigator's decision to withdraw the patient
- patient withdraws consent
- pregnancy of the patient
- non-compliance with trial treatment or procedure requirements.

Assessment during re-treatment
Assessments for patients who are re-treated with Pembrolizumab are as per section 10.2 and listed in Appendix 1 – Trial Schedule of Procedures/Assessments.

Patients will need to provide consent for the re-treatment as per section 6.

DocuSign Envelope ID: F89E9DEB-F629-4294-B1D8-B570A4FACE07

9.4. Dose Management/ Delays not related to IMP Adverse Events

Dosing interruptions are permitted in the case of medical / surgical events or logistical reasons not related to study therapy (e.g. elective surgery and/or patient's holidays). Patients should be placed back on study treatment within 3 weeks of the scheduled interruption, unless otherwise discussed with the Sponsor. The reason for interruption should be documented in the patient's study record.

Dose modifications are not permitted.

9.5. Management of Adverse Events

AEs associated with pembrolizumab exposure may represent an immunologic etiology. These immune-related AEs (irAEs) may occur shortly after the first dose or several months after the last dose of pembrolizumab treatment and may affect more than one body system simultaneously. Therefore, early recognition and initiation of treatment is critical to reduce complications. Based on existing clinical study data, most irAEs were reversible and could be managed with interruptions of pembrolizumab, administration of corticosteroids and/or other supportive care. For suspected irAEs, ensure adequate evaluation to confirm etiology or exclude other causes. Additional procedures or tests such as bronchoscopy, endoscopy, skin biopsy may be included as part of the evaluation. Based on the severity of irAEs, withhold or permanently discontinue pembrolizumab and administer corticosteroids. Dose management guidelines for irAEs associated with pembrolizumab are provided in Table 4.

Table 4: Management Guidelines for Drug-Related Adverse Events

General instructions:

- Corticosteroid taper should be initiated upon AE improving to Grade 1 or less and continue to taper over at least 4 weeks to less than or equal to 10mg (or equivalent) of prednisolone.

  NOTE:
  For some immune-related AEs (please see list in the table below) pembrolizumab should be permanently discontinued in case of a G3 or G4 immune-related AE.
    - For participants with Grade 3 or 4 immune-related endocrinopathy where pembrolizumab is withheld, pembrolizumab may be resumed when AE resolves to ≤ Grade 2 and is controlled with thyroid replacement therapy (hypothyroidism), non-selective beta-blockers (eg, propranolol) or thionamides (hyperthyroidism) or in the case of hyperglycaemia, metabolic control is achieved (in case of T1DM).

- For situations where pembrolizumab has been withheld, pembrolizumab can be resumed after AE has been reduced to Grade 1 or 0 and corticosteroid has been tapered (unless it is specified to permanently discontinue pembrolizumab in case of G3 or G4 immune-related AE in the table below). **Pembrolizumab should be permanently discontinued if AE does not resolve within 12 weeks of last dose or corticosteroids cannot be reduced to ≤10 mg prednisolone or equivalent per day within 12 weeks.**

- For severe and life-threatening irAEs, IV corticosteroid should be initiated first followed by oral steroid. Other immunosuppressive treatment should be initiated if irAEs cannot be controlled by corticosteroids.

**Immune-related AEs**	**Toxicity grade or conditions (CTCAEv5.0)**	**Action taken to pembrolizumab**	**irAE management with corticosteroid and/or other therapies**	**Monitor and follow-up**
Pneumonitis	Grade 2	Withhold	• Administer corticosteroids (initial dose of 1-2 mg/kg prednisolone or equivalent) followed by taper<br>• Add prophylactic antibiotics for opportunistic infections	• Monitor participants for signs and symptoms of pneumonitis<br>• Evaluate participants with suspected pneumonitis with radiographic imaging and initiate corticosteroid treatment<br>•
	Grade 3 or 4, or recurrent Grade 2	Permanently discontinue		
Diarrhoea / Colitis	Grade 2 or 3 (also see below with respect to *recurrent* grade 3)	Withhold	• Administer corticosteroids (initial dose of 1-2 mg/kg prednisolone or equivalent) followed by taper	• Monitor participants for signs and symptoms of enterocolitis (ie, diarrhea, abdominal pain, blood or mucus in stool with or without fever) and of bowel perforation (ie, peritoneal signs and ileus).<br>• Participants with ≥ Grade 2 diarrhea suspecting colitis should consider GI consultation and performing endoscopy to rule out colitis.<br>• Participants with diarrhea/colitis should be advised to drink
	Recurrent Grade 3 or Grade 4	Permanently discontinue		

				liberal quantities of clear fluids. If sufficient oral fluid intake is not feasible, fluid and electrolytes should be substituted via IV infusion.
AST / ALT elevation or Increased bilirubin	Grade 2	Withhold	• Administer corticosteroids (initial dose of 0.5- 1 mg/kg prednisolone or equivalent) followed by taper	• Monitor with liver function tests (consider weekly or more frequently until liver enzyme value returned to baseline or is stable
	Grade 3 or 4	Permanently discontinue	• Administer corticosteroids (initial dose of 1-2 mg/kg prednisolone or equivalent) iv for 24-48 hours followed by oral taper	
Type 1 diabetes mellitus (T1DM) or Hyperglycaemia	New onset T1DM or Grade 3 or 4 hyperglycaemia associated with evidence of β-cell failure measured by insulin in serum	Withhold	• Initiate insulin replacement therapy for participants with T1DM<br>• Administer anti-hyperglycaemic in participants with hyperglycaemia	• Monitor participants for hyperglycaemia or other signs and symptoms of diabetes.
Hypophysitis	Grade 2	Withhold	• Administer corticosteroids and initiate hormonal replacements as clinically indicated.	• Monitor for signs and symptoms of hypophysitis (including hypopituitarism and adrenal insufficiency)
	Grade 3 or 4	Withhold or permanently discontinue*		
Hyperthyroidism	Grade 2	Continue	• Treat with non-selective beta-blockers (eg, propranolol) or thionamides as appropriate	• Monitor for signs and symptoms of thyroid disorders.
	Grade 3 or 4	Withhold or permanently discontinue *		
Hypothyroidism	Grade 2-4	Continue	• Initiate thyroid replacement hormones (eg, levothyroxine or liothyronine) per standard of care	• Monitor for signs and symptoms of thyroid disorders.
Myocarditis	Grade 1	Withhold	• Based on severity of AE administer corticosteroids	• Ensure adequate evaluation to confirm etiology and/or exclude other causes
	Grade 2, 3 or 4	Permanently discontinue		
Nephritis grading according to increased creatinine or acute kidney injury	Grade 2	Withhold	• Administer corticosteroids (prednisolone 1-2 mg/kg or equivalent) followed by taper.	• Monitor changes of renal function
	Grade 3 or 4	Permanently discontinue		

DocuSign Envelope ID: F89E9DEB-F629-4294-B1D8-B570A4FACE07

Neurological Toxicities	Grade 2	Withhold	• Based on severity of AE administer corticosteroids	• Ensure adequate evaluation to confirm etiology and/or exclude other causes
	Grade 3 or 4	Permanently discontinue		
Exfoliative Dermatologic Conditions	Suspected SJS, TEN, or DRESS	Withhold	• Based on severity of AE administer corticosteroids	• Ensure adequate evaluation to confirm etiology or exclude other causes
	Confirmed SJS, TEN, or DRESS	Permanently discontinue		
All other immune-related AEs	Intolerable/ persistent Grade 2	Withhold	• Based on type and severity of AE administer corticosteroids	• Ensure adequate evaluation to confirm etiology and/or exclude other causes
	Grade 3	Withhold or discontinue based on the type of event. Events that require discontinuation include and not limited to: encephalitis and other clinically important irAEs.		
	Grade 4 or recurrent Grade 3	Permanently discontinue		
* For Hypophysitis or Hyperthyroidism withholding or permanently discontinue pembrolizumab is at the discretion of the investigator or treating physician. Note: Non-irAE will be managed as appropriate, following clinical practice recommendations.				

Sites must always check the current Pembrolizumab IB in conjunction with the protocol. The following should be observed:

- **Pneumonitis (Interstitial lung disease)**:

  Pneumonitis (including fatal cases) has been reported in patients receiving Pembrolizumab. Monitor patients for signs and symptoms of pneumonitis. If pneumonitis is suspected, evaluate with radiographic imaging and exclude other causes.

  - For **Grade 2 events**, treat with systemic corticosteroids. When symptoms improve to Grade 1 or less, steroid taper should be started and continued over no less than 4 weeks. For recurrent moderate grade 2 pneumonitis, permanently discontinue pembrolizumab.
  - For **Grade 3-4 events**, immediately treat with intravenous steroids. Administer additional anti-inflammatory measures, as needed and permanently discontinue pembrolizumab for severe (Grade 3), life threatening (Grade 4).
  - Add prophylactic antibiotics for opportunistic infections in the case of prolonged steroid administration.

- **Diarrhoea/Colitis**:

DocuSign Envelope ID: F89E9DEB-F629-4294-B1D8-B570A4FACE07

Patients should be carefully monitored for signs and symptoms of enterocolitis (such as diarrhoea, abdominal pain, blood or mucus in stool, with or without fever) and of bowel perforation (such as peritoneal signs and ileus).

- All patients who experience diarrhoea/colitis should be advised to drink liberal quantities of clear fluids. If sufficient oral fluid intake is not feasible, fluid and electrolytes should be substituted via IV infusion. For Grade 2 or higher diarrhoea, consider GI consultation and endoscopy with biopsy (to exclude microscopic colitis) to confirm or rule out colitis.
- For **Grade 2 diarrhoea/colitis,** administer oral corticosteroids.
- For **Grade 3 or 4 diarrhoea/colitis,** treat with intravenous steroids followed by high dose oral steroids.
- When symptoms improve to Grade 1 or less, steroid taper should be started and continued over no less than 4 weeks.

- **Type 1 diabetes mellitus (if new onset, including diabetic ketoacidosis [DKA]) or ≥ Grade 3 Hyperglycaemia, if associated with ketosis (ketonuria) or metabolic acidosis (DKA):**
  - For **T1DM** or **Grade 3-4** Hyperglycaemia
    - Insulin replacement therapy is recommended for Type I diabetes mellitus and for Grade 3-4 hyperglycaemia associated with metabolic acidosis or ketonuria.
    - Evaluate patients with serum glucose and a metabolic panel, urine ketones, glycosylated haemoglobin, and C-peptide.
- **Hypophysitis**:
  - For **Grade 2** events, treat with corticosteroids. When symptoms improve to Grade 1 or less, steroid taper should be started and continued over no less than 4 weeks. Replacement of appropriate hormones may be required as the steroid dose is tapered.
  - For **Grade 3-4** events, treat with an initial dose of IV corticosteroids followed by oral corticosteroids. When symptoms improve to Grade 1 or less, steroid taper should be started and continued over no less than 4 weeks. Replacement of appropriate hormones may be required as the steroid dose is tapered.
- **Hyperthyroidism or Hypothyroidism**:

  Thyroid disorders can occur at any time during treatment. Monitor patients for changes in thyroid function (at the start of treatment, periodically during treatment, and as indicated based on clinical evaluation) and for clinical signs and symptoms of thyroid disorders.
  - **Grade 2** hyperthyroidism events (and **Grade 2-4** hypothyroidism):
    - In hyperthyroidism, non-selective beta-blockers (e.g. propranolol) are suggested as initial therapy.
    - In hypothyroidism, thyroid hormone replacement therapy, with levothyroxine or liothyroinine, is indicated per standard of care.

DocuSign Envelope ID: F89E9DEB-F629-4294-B1D8-B570A4FACE07

- **Grade 3-4** hyperthyroidism:
  - Treat with an initial dose of IV corticosteroid followed by oral corticosteroids. When symptoms improve to Grade 1 or less, steroid taper should be started and continued over no less than 4 weeks. Replacement of appropriate hormones may be required as the steroid dose is tapered.

- **Hepatic**:
  - For **Grade 2** events, monitor liver function tests more frequently until returned to baseline values (consider weekly).
    - Treat with IV or oral corticosteroids
  - For **Grade 3-4** events, treat with intravenous corticosteroids for 24 to 48 hours.
  - When symptoms improve to Grade 1 or less, a steroid taper should be started and continued over no less than 4 weeks.
- **Renal Failure or Nephritis**:
  - For **Grade 2** events, treat with corticosteroids.
  - For **Grade 3-4** events, treat with systemic corticosteroids.
  - When symptoms improve to Grade 1 or less, steroid taper should be started and continued over no less than 4 weeks.
- **Stevens-Johnson syndrome (SJS) and toxic epidermal necrolysis (TEN):**

  Cases of SJS and TEN, some with fatal outcome, have been reported in patients treated with pembrolizumab. For signs or symptoms of SJS or TEN, withhold pembrolizumab and refer the patient for specialised care for assessment and treatment. If SJS or TEN is confirmed, permanently discontinue pembrolizumab.

- **Other important potential risks include:** Based upon additional information received from the clinical study and postmarketing environments immune-mediated ADRs may be fatal and may occur after discontinuation of pembrolizumab.
- Arthritis adverse reactions have been identified during post-approval use of pembrolizumab. As these reactions are reported voluntarily from a population of uncertain size, it is not always possible to reliably estimate their frequency or establish a causal relationship to drug exposure.
- Immune-mediated irARs such as myocarditis, adrenal insufficiency, thyroiditis, uveitis, cholangitis, myositis, encephalitis and lung disease may occur. If a patient experiences any of these events, consult relevant medical specialists for management as per local policies.

Management of Infusion Reactions:

DocuSign Envelope ID: F89E9DEB-F629-4294-B1D8-B570A4FACE07

Signs and symptoms usually develop during or shortly after drug infusion and generally resolve completely within 24 hours of completion of infusion. Infusion-related reactions may present as allergic reaction (hypersensitivity), serum sickness, infusion and infusion-like reactions, cytokine release syndrome, or anaphylaxis. In the case of severe or life-threatening reactions, subsequent doses of pembrolizumab should not be administered.

Table 5 shows management guidelines for patients who experience an infusion reaction associated with administration of pembrolizumab (MK-3475).

Table 5: Infusion Reaction Management Guidelines

NCI CTCAE v5 Grade	Treatment	Premedication at subsequent dosing
Grade 1<br>Mild reaction; infusion interruption not indicated; intervention not indicated	Increase monitoring of vital signs as medically indicated until the patient is deemed medically stable in the opinion of the investigator.	None
Grade 2<br>Requires infusion interruption but responds promptly to symptomatic treatment (e.g., antihistamines, NSAIDS, narcotics, IV fluids); prophylactic medications indicated for < =24 hrs	**Stop Infusion and monitor symptoms.**<br>Additional appropriate medical therapy may include but is not limited to:<br>IV fluids<br>Antihistamines<br>NSAIDS<br>Paracetamol<br>Narcotics<br>Increase monitoring of vital signs as medically indicated until the patient is deemed medically stable in the opinion of the investigator.<br>If symptoms resolve within one hour of stopping drug infusion, the infusion may be restarted at 50% of the original infusion rate (e.g., from 100 mL/hr to 50 mL/hr). Otherwise dosing will be held until symptoms resolve and the patient should be premedicated for the next scheduled dose.<br>**Patients who develop Grade 2 toxicity despite adequate premedication should be permanently discontinued from further trial treatment administration.**	Patient may be premedicated 1.5h (± 30 minutes) prior to infusion of pembrolizumab (MK-3475) with:<br>Antihistamines 50 mg po (or equivalent dose of antihistamine).<br><br>Paracetamol 500-1000 mg po (or equivalent dose of antipyretic).
Grades 3 or 4<br>Grade 3:<br>Prolonged (i.e., not rapidly responsive to symptomatic medication and/or brief interruption of infusion); recurrence of symptoms following initial improvement; hospitalisation indicated for other clinical sequelae (e.g., renal impairment, pulmonary infiltrates)<br>Grade 4:<br>Life-threatening; pressor or ventilatory support indicated	**Stop Infusion.**<br>Additional appropriate medical therapy may include but is not limited to:<br>IV fluids<br>Antihistamines<br>NSAIDS<br>Paracetamol<br>Narcotics<br>Oxygen<br>Pressors<br>Corticosteroids<br>Epinephrine<br><br>Increase monitoring of vital signs as medically indicated until the patient is deemed medically stable in the opinion of the investigator.<br>Hospitalisation may be indicated.	No subsequent dosing

DocuSign Envelope ID: F89E9DEB-F629-4294-B1D8-B570A4FACE07

NCI CTCAE v5 Grade	Treatment	Premedication at subsequent dosing
	**Patient is permanently discontinued from further trial treatment administration.**	
Appropriate resuscitation equipment should be available in the room and a physician readily available during the period of drug administration.		

9.6. Management of Overdoses, Trial treatment error, or Occupational Exposure

Overdose

For purposes of this trial, an overdose of pembrolizumab will be defined as any dose of greater than 200mg. No specific information is available on the treatment of overdose of pembrolizumab. Appropriate supportive treatment should be provided if clinically indicated. In the event of overdose, the patient should be observed closely for signs of toxicity. Appropriate supportive treatment should be provided if clinically indicated.

Overdoses should be reported on an incident report (see section 14.1). Any adverse events resulting from an overdose should be reported as an SAE (see section 13.2.2 for reporting procedures). Overdoses of pembrolizumab that are in excess of 5 x the protocol specified dose (i.e. >1000mg), with or without adverse events resulting from an overdose, should be reported as an AESI (see section 13.4, Adverse event of special interest, for reporting procedures)***.***

Trial treatment error

Any unintentional error in prescribing, dispensing, or administration of a trial treatment while in the control of a healthcare professional or consumer. The error can be identified either by the trial team at site or by the Sponsor upon review.

Trial Treatment errors should be reported on an incident report (see section 14.1). Any adverse events resulting from a medication error should be reported as an SAE (see section 13.2.2 for reporting procedures).

Occupational exposure

Exposure to a trial treatment as a result of one's professional or non-professional occupation. Occupational exposure should be reported on an incident report form (see section 14.1).

9.7. Supportive Care

Patients should receive appropriate supportive care measures as deemed necessary by the treating investigator. Suggested supportive care measures for the management of adverse events with potential immunologic aetiology are outlined below. Where appropriate, these guidelines include the use of oral or intravenous treatment with

DocuSign Envelope ID: F89E9DEB-F629-4294-B1D8-B570A4FACE07

corticosteroids as well as additional anti-inflammatory agents if symptoms do not improve with administration of corticosteroids. Note that several courses of steroid tapering may be necessary as symptoms may worsen when the steroid dose is decreased. For each disorder, attempts should be made to rule out other causes such as metastatic disease or bacterial or viral infection, which might require additional supportive care. The management guidelines are intended to be applied when the investigator determines the events to be related to pembrolizumab.

Note: if after the evaluation the event is determined not to be related, the investigator does not need to follow the treatment guidance (as outlined in section 9.5). Refer to Section 9.4 for dose delay.

It may be necessary to perform additional procedures such as bronchoscopy, endoscopy, or skin photography as part of evaluation of the event.

9.8. Contraindications

Medications or vaccinations specifically prohibited in the exclusion criteria in section 7.2.2 are not allowed during the ongoing trial. If there is a clinical indication for one of these or other medications or vaccinations specifically prohibited during the trial, discontinuation from trial therapy may be required. The investigator should discuss any questions regarding this with the Sponsor. The final decision on any supportive therapy or vaccination rests with the investigator and/or the patient's primary physician.

9.8.1. Acceptable Concomitant Medications

All treatments that the investigator considers necessary for a patient's welfare may be administered at the discretion of the investigator in keeping with the community standards of medical care. All concomitant medication will be recorded on the eCRF including all prescription, over-the-counter (OTC), herbal supplements, and IV medications and fluids. If changes occur during the trial period, documentation of drug dosage, frequency, route, and date may also be included on the eCRF. The latest version of the IB should be checked to avoid giving the patient prohibited concomitant medications.

All concomitant medications received within 28 days before the first dose of trial treatment and 30 days after the last dose of trial treatment should be recorded. Concomitant medications administered after 30 days after the last dose of trial treatment should be recorded for SAEs and AESIs as defined in Section 13.

COVID-19 Vaccinations:

Even though no interaction studies have been performed, the PEACOCC Trial Management Group approve of the use of COVID-19 vaccines in existing PEACOCC participants in the absence of any other contraindications without treatment interruption, and would not exclude future potential participants if they have received a COVID-19 vaccination.

DocuSign Envelope ID: F89E9DEB-F629-4294-B1D8-B570A4FACE07

The timing between COVID-19 vaccine administration and trial IMP (pembrolizumab) should be determined by the Principal Investigator or a Co-investigator at site in the best interests of the patient. However, the PEACOCC TMG recommend that there should be a 24-hour gap between vaccine administration and patients receiving pembrolizumab. If you have any questions or would like advice from the PEACOCC TMG please email the trial team at ctc.peacocc@ucl.ac.uk. Details of vaccine administration should be added to the concomitant medication eCRF (if received with the timeframe for reporting concomitant medication) and recorded in the patient's medical notes.

9.8.2. Prohibited Concomitant Medications\Therapies

Patients are prohibited from receiving the following therapies during the screening and treatment period (including retreatment for post-complete response relapse) of this trial:

- Antineoplastic systemic chemotherapy, endocrine therapy or biological therapy except for treatment to manage hypercalcaemia of malignancy (for example bisphosphonates, denosumab).
- Immunotherapy not specified in this protocol.
- Chemotherapy not specified in this protocol.
- Investigational agents other than pembrolizumab.

  Radiation therapy

  Note: Radiation therapy to a symptomatic solitary lesion or to the brain may be allowed at the investigator's discretion. If the radiation is delivered to a RECIST v1.1 target lesion which is already used to assess response within the trial, this must first be discussed with the sponsor.

  - Live vaccines within 30 days prior to the first dose of trial treatment and while participating in the trial (which includes follow-up). Examples of live vaccines include, but are not limited to, the following: measles, mumps, rubella, varicella/zoster, yellow fever, rabies, BCG, and typhoid vaccine.

- Systemic glucocorticoids for any purpose other than to modulate symptoms from an event of clinical interest of suspected immunologic etiology. The use of physiologic doses of corticosteroids at daily doses equivalent to or less than 10mg prednisolone is allowed.

Patients who, in the assessment by the investigator, require the use of any of the aforementioned treatments for clinical management should be removed from receiving trial treatment and must let UCL CTC know if this occurs. Patients may receive other medications that the investigator deems to be medically necessary.

The Exclusion Criteria describes other medications which are prohibited in this trial.

9.9. Pharmacy Responsibilities

Oversight of all pharmacy aspects of the trial at participating sites is the responsibility of the PI, who may delegate responsibility of specific pharmacy related tasks to the local pharmacist or other appropriately qualified personnel, who will be the Pharmacy Lead. The delegation of duties must be recorded on the site staff delegation log.

Pembrolizumab supplied for the PEACOCC trial are for PEACOCC trial patients only and must not be used outside the context of this protocol.

9.9.1. Storage and Handling Requirements

Clinical supplies must be stored in a secure, limited-access location under the storage conditions specified on the label. Receipt and dispensing of trial medication must be recorded by an authorised person at the trial site. Clinical trial supplies may not be used for any purpose other than that stated in the protocol.

9.9.2. Temperature Excursions

Pembrolizumab drug product must be stored under refrigerated conditions (2°C to 8°C). All temperature excursions outside the storage conditions specified in the IB, SoDA and label must be reported to UCL CTC as per the ‘Pharmacy Procedure for Reporting Temperature Excursions’ (see Pharmacy Site File).

Upon identifying an excursion:

- all affected trial stock must be quarantined IMMEDIATELY.
- the ‘Notification of Temperature Excursion’ form must be completed and e-mailed to ctc.excursions@ucl.ac.uk

Please note that UCL CTC must be informed immediately if any patient has been administered drug affected by a temperature excursion.

9.9.3. Study IMP Accountability

The Pharmacy Lead must ensure that appropriate records are maintained.

These records must include accountability for the study IMP including: receipt, dispensing, reconciliation and destruction of unused medication (on sponsor authorisation). Accountability forms will be supplied, and must be used, unless there is prior agreement from UCL CTC to use alternative in-house records.

Copies of completed drug accountability logs must be submitted to UCL CTC for all trial patients upon request. Also refer to section 15.2 (Centralised Monitoring).

9.9.4. Returns and Reconciliation

The investigator and lead pharmacist are responsible for keeping accurate records of the clinical trial supplies received from MSD or designee, the amount dispensed to the patients and the amount remaining in stock prior to the conclusion of the trial.

Upon completion or termination of the study, all unused and/or partially used investigational product will be destroyed at the site per institutional policy. It is the Investigator's responsibility to arrange for disposal of all empty containers, provided that procedures for proper disposal have been established according to applicable federal, state, local and institutional guidelines and procedures, and provided that appropriate records of disposal are kept.

9.10. 24 Hour/Out-of-Office Hours Emergency Drug-Specific Advice

**Pembrolizumab**	**Office hours**<br>09:00 to 17:00 Monday to Friday, excluding Bank Holidays<br><br>Contact UCL CTC<br>+44 207 679 9284	**All other times**<br><br>Contact MSD<br>+44 208 154 8000

9.11. Clinical Management after Treatment Discontinuation

If a patient discontinues trial treatment early, they will remain on trial for follow up purposes unless they explicitly withdraw consent. Also refer to sections 10 (Assessments) and 16 (Withdrawal of Patients) for further details regarding treatment discontinuation, patient withdrawal from trial treatment and withdrawal of consent to data collection.

9.12. Drug Provision during the trial

During the trial, study IMP, Pembrolizumab will be provided to patients for a maximum of two years in the initial treatment stage.. Patients who stop pembrolizumab with stable disease or better may be eligible for up to one year of re-treatment as per section 9.3.3.

DocuSign Envelope ID: F89E9DEB-F629-4294-B1D8-B570A4FACE07

10. ASSESSMENTS/TRIAL PROCEDURES

The Trial Schedule of Procedures/Assessments table (Appendix 1) summarises the trial procedures to be performed prior or at each visit. Individual trial procedures are described in detail below. It may be necessary to perform these procedures at unscheduled time points if deemed clinically necessary by the investigator.

10.1. Pre-registration Assessments/Procedures

Please refer to section 8.1.1 Pre-registration Assessments

10.2. Assessments During Treatment

During treatment (initial and re-treatment) the patient should be seen every 3 weeks from the start of treatment and the following assessments/investigations/procedures. These assessments need to be performed within 3 days prior to day 1 of each cycle, after cycle 1 (Please also see Appendix 1), unless otherwise indicated below:

- **Full physical exam**
- **Vital signs and weight**
- **ECOG PS**
- **Adverse events** review
- **Concomitant medication** review
- **CT scan of chest, assessable by RECIST v1.1**
- **CT scan or MRI scan** assessable by RECIST v1.1 (abdomen, pelvis, and brain, if patient shown to have brain metastases at baseline) (at week 6, week 12 and then every 12 weeks). If a patient's treatment is delayed, the scans should still be performed at these given timepoints where feasible.
- **Full blood count**
- **Liver function tests, urea & electrolytes**
- **Thyroid function tests** (Total T3/Free T4 and TSH)
- **Serum calcium**
- **Urinalysis**
- **Urine or serum pregnancy test** (if applicable)
- **CA 125**
- **Biopsy** (required at 6-8 weeks following treatment initiation)*.
- **Exploratory research bloods:** PBMCs and plasma – please refer to the lab manual.
- **QoL: FACT-O** (at week 6, week 12 and then every 12 weeks).

*In the event of any local covid-19 restrictions impacting resource at site to perform the biopsy as scheduled, the biopsy may be performed at a subsequent cycle, if it becomes possible and safe to do so later on. Site must contact the CTC prior to this been re-arranged for approval from the CI/TMG.

10.2.1. Adverse Event (AE) Monitoring

The investigator or qualified designee will assess each patient to evaluate for potential new or worsening AEs and more frequently if clinically indicated. AEs will be graded and recorded throughout the study and during the follow-up period according to NCI CTCAE Version 5. AEs will be characterised in terms regarding seriousness, causality, grading, and action taken with regard to trial treatment.

Please refer to Section 13.2 for detailed information regarding the assessment and recording of AEs.

10.2.2. Full Physical Exam

The investigator or qualified designee will perform a complete physical exam at each scheduled trial visit.

10.2.3. Vital Signs

The investigator or qualified designee will take vital signs at screening, prior to the administration of each dose of trial treatment and at treatment discontinuation as specified in (Appendix 1). Vital signs should include temperature, heart rate, respiratory rate, weight, blood pressure and O2 saturation. Height will be measured at baseline only.

10.2.4. ECOG Performance status

The investigator or qualified designee will assess ECOG status at baseline, prior to the administration of each dose of trial treatment and discontinuation of trial treatment as specified in the schedule of events (Appendix 1).

10.2.5. Laboratory Procedures/Assessments

Details regarding specific laboratory procedures/assessments to be performed in this trial are provided below laboratory safety evaluations (Haematology, Chemistry and Urinalysis). Laboratory tests for haematology, chemistry, urinalysis, and others are specified in Table 6.

Table 6 Laboratory Tests

Haematology	Chemistry	Urinalysis	Other
	Albumin	Blood	Serum β-human chorionic gonadotropin†
Haemoglobin	Alkaline phosphatase	Glucose	(β-hCG)†
Platelet count	Alanine aminotransferase (ALT)	Protein	PT (INR)
WBC (total and differential)	Aspartate aminotransferase (AST)		
	Lactate dehydrogenase (LDH)		Total triiodothyronine (T3)
Absolute Neutrophil Count		results are noted	Free thyroxine (T4)
		Urine pregnancy test †	Thyroid stimulating hormone (TSH)
	Calcium		
	Chloride		Blood for exploratory research studies
	Glucose		
	Phosphorus		
	Potassium		

Haematology	Chemistry	Urinalysis	Other
	Sodium		
	Magnesium		
	Total Bilirubin		
	Direct Bilirubin *(If total bilirubin is elevated above the upper limit of normal)*		
	Creatinine		
† Perform on women of childbearing potential only. If urine pregnancy results cannot be confirmed as negative, a serum pregnancy test will be required.<br>‡ If considered standard of care in your region.			

Laboratory tests for baseline entry for initial treatment and entry into the re-treatment period should be performed within the 10 days prior to registration. After Cycle 1, pre-dose laboratory procedures can be conducted up to 72 hours prior to dosing. Results must be reviewed by the investigator or qualified designee and found to be acceptable prior to each dose of trial treatment.

10.3. Tumour Imaging and assessment of disease

10.3.1. Evaluation of Efficacy

Tumour assessments will be based on RECIST v1.1 and will be performed according to the schedule presented in Appendix 1. Tumour assessments will be performed at baseline, after cycle 2 (6 weeks on treatment) and after cycle 4 (12 weeks on treatment) and thereafter after every 4 cycles (12 weekly) until progression.

10.3.2. Tumour Assessment

Tumour assessments can include either cross-sectional imaging using CT or magnetic resonance imaging (MRI) scan of the chest, abdomen, and pelvis; CT or MRI scan of the brain will be performed at baseline. Post- baseline brain imaging will be performed for all patients with brain metastases at baseline or if the patient becomes neurologically symptomatic. The preferred method of disease assessment is CT with contrast. If CT with contrast is contraindicated, CT without contrast or MRI with contrast may be used. The same method is required for all subsequent tumour assessments.

10.3.2.1. CT scans with contrast to the chest, abdomen, pelvis, and brain

CT scans should be performed with contiguous cuts in slice thickness of 5 mm or less. Spiral CT should be performed using a 5 mm contiguous reconstruction algorithm.

10.3.2.2. MRI Imaging scans

MRI of the chest, abdomen and pelvis, and brain is acceptable for measurement of lesions provided that the same anatomical plane is used for serial assessments. If possible, the same imaging device should be used for serial evaluations. In case of MRI, measurements will be preferably performed in the axial (transverse) plane on contrast-enhanced T1-weighted images. However, there are no specific sequence recommendations.

DocuSign Envelope ID: F89E9DEB-F629-4294-B1D8-B570A4FACE07

10.4. Other Procedures

10.4.1. Withdrawal/ Discontinuation

When a patient discontinues/withdraws prior to trial completion, all applicable activities scheduled for the final trial visit should be performed. Any adverse events should be reported in accordance with the safety requirements outlined in Section 13.2. Patients who a) attain a CR or b) complete 24 months of treatment with pembrolizumab may discontinue treatment with the option of restarting treatment if they meet the criteria specified in Section 9.3.3 and the trial remains open. After discontinuing treatment, patients should return to the site for the end of treatment visit as per section 10.5 and then proceed to the Follow-Up period of the study (as per section 10. 6).

10.4.2. Visit Requirements

Please also refer to tables of assessments outlined in Appendix 1 'Schedule of Procedures/Assessments'.

10.4.2.1. Baseline

Section 10.1 shows all procedures to be conducted at the baseline visit. All baseline investigations must be completed within the 28 days prior to registration unless otherwise indicated.

10.4.2.2. Treatment period

Procedures to be conducted during the initial and re-treatment period are presented in Appendix 1 'Schedule of Procedures/Assessments'. At study visits, when patients do not receive investigational product, all of the pre-treatment cycle assessments will be performed. All samples are collected pre-dose unless otherwise indicated.

10.4.2.3 Post-Treatment Visits

Appendix 1 'Schedule of Procedures/Assessments' shows all procedures to be conducted during the follow-up period.

All patients are required to attend the end of treatment visit, all follow-up visits and to be contacted by the research team for survival status in accordance with the Schedule of Procedures/Assessments in Appendix 1. However, if a patient discontinues from treatment, has progressed and moves onto alternative anticancer treatment, the follow-up visits will no longer be required; however, survival follow-up assessments and collection of subsequent anticancer therapy information would be required as indicated in the Schedule of Assessments/Procedures, Appendix 1 unless the patient withdraws consent for further survival follow-up. Survival follow-up will continue until the end of study.

DocuSign Envelope ID: F89E9DEB-F629-4294-B1D8-B570A4FACE07

10.5. Assessments on Completion of Trial Treatment

After the end of initial and re-treatment periods, each patient will have an end of treatment visit which will be conducted after 30 ± 7 days from last treatment administration / decision to discontinue, before entering the follow-up period. If previous imaging was obtained within 4 weeks prior to the date of discontinuation, then imaging at treatment discontinuation is not mandatory. In participants who discontinue study treatment due to documented disease progression, this is the final required trial tumour imaging.

All AEs that occur during this period should be recorded.

Patients who are eligible for re-treatment with pembrolizumab (as described in Section 9.3.3) may have up to two end of treatment follow-up visits, one after the initial treatment period and one after the re-treatment period.

Assessments to be completed at the end of treatment are:

- **Concomitant Medication review**
- **Adverse Events review**
- **Additional anti-cancer treatment review**
- **Survival status**
- **Full physical exam**
- **Vital signs and weight**
- **ECOG PS**
- **CT scan assessable by RECIST v1.1**
- **CT scan or MRI scan** assessable by RECIST v1.1) (abdomen, pelvis, and brain (if patient assessed with brain metastases at baseline), if the patient has not stopped treatment due to progression)
- **Full blood count**
- **Liver function tests, urea & electrolytes**
- **Thyroid function tests (Total T3/Free T4 and TSH)**
- **Serum calcium**
- **Urinalysis**
- **Urine or serum pregnancy test** (if applicable)
- **CA 125**
- **Biopsy** (if patient stopped due to confirmation of progressive disease) – should be performed within 6 weeks of patient discontinuing treatment*.
- **Exploratory research bloods** (if patient stopped due to progression): PBMCs and plasma, please refer ot the lab manual for details.
- **QoL: FACT-O**

*In the event of any local covid-19 restrictions impacting resource at site to perform the biopsy as scheduled, the biopsy may be performed at a later timepoint, if it becomes possible and safe to do so later on, and as long as the patient has not proceeded with further cancer treatment Site must contact the CTC prior to this been re-arranged for approval from the CI/TMG.

DocuSign Envelope ID: F89E9DEB-F629-4294-B1D8-B570A4FACE07

10.6. Assessments During Follow Up (before progression)

Patients will have post-treatment follow-up for disease status, until disease progression, death, withdrawing consent, or becoming lost to follow-up.

These patients who discontinue trial treatment for a reason other than disease progression should be assessed **every 12 weeks** (84 ± 7 days), following the end of treatment, by radiologic imaging to monitor disease status. Every effort should be made to collect information regarding disease status until disease progression, death, end of the study or if the patient begins re-treatment with pembrolizumab as detailed in Section 9.3.3. Information regarding post-study anti-cancer treatment will be collected if new treatment is initiated.

Patients who are eligible and have consented to receive re-treatment with pembrolizumab according to the criteria in Section 9.3.3 will move from the follow-up phase to the re-treatment period when they experience disease progression.

Assessments to be completed during follow-up are:

- **Adverse Events review** as per requirements detailed in the pharmacovigilance sections 13.2.1 (AE), 13.2.2 (SAE) and 13.4 (AESI)
- **CT scan of chest, assessable by RECIST v1.1**
- **CT scan or MRI scan** assessable by RECIST v1.1) (abdomen, pelvis, and brain (if patient assessed with brain metastases at baseline)
- **Additional anti-cancer treatment review**
- **Survival status**
- **QoL: FACT-O**
- **Biopsy** (if first disease progression on the trial during the follow-up period has been confirmed) – should be performed within 6 weeks*.

*In the event of any local covid-19 restrictions impacting resource at site to perform the biopsy as scheduled, the biopsy may be performed at a later timepoint, if it becomes possible and safe to do so later on, and as long as the patient has not proceeded with further cancer treatment Site must contact the CTC prior to this been re-arranged for approval from the CI/TMG.

10.6.1. Survival Follow-up

Once a patient experiences confirmed disease progression or starts a new anti-cancer therapy, the patient moves into the survival follow-up phase and should be contacted by telephone every 12 weeks to assess for survival status and any AEs that require reporting as per section 13.2, until death, withdrawal of consent, or the end of the study, whichever occurs first.

DocuSign Envelope ID: F89E9DEB-F629-4294-B1D8-B570A4FACE07

11. EXPLORATORY BIOLOGICAL STUDIES

11.1. Tumour Tissue Collection and Exploratory Research Blood Sampling

One of the exploratory aspects of this study is to evaluate potential tumour and peripheral blood biomarkers including exome and RNA sequencing. with the objective of identifying biomarkers that predict response to pembrolizumab in patients with advanced clear cell gynaecological cancers Patient consent to blood and tumour sample collection is included in the main trial consent form.

Detailed information on sample handling, storage and shipping are included in the Laboratory Manual.

11.1.1. Biomarker Sample Collection

For each patient, the following samples will be collected (for both the initial and re-treatment periods) - please see the laboratory manual for specific guidance:

- Whole blood EDTA at baseline
- Circulating PBMCs at baseline, with cycles 2, 4, 6 then at 12 months and at the time of disease progression
- Circulating plasma at baseline, with cycles 2, 4, 6 then at 12 months and at the time of disease progression
- Archival tissue that was used to confirm diagnosis (from initial treatment)
- Fresh tumour tissue biopsy samples at baseline, 6-8 weeks during treatment and at the time if disease progression.

11.1.2. Tumour Biopsies

At baseline, the fresh tumour biopsy will be obtained prior to administration of the first dose of pembrolizumab for all patients. The next fresh tumour biopsy will be obtained between Day 42 and 56 (± 7 days) from first day of initial treatment and re-treatment if applicable and within 8 weeks of diagnosis of progressive disease (initial and re-treatment phases).

N.B. The requirement for sites to request a biopsy from the patient is mandatory at baseline, 6-8 weeks and upon progression during the initial treatment period, but optional during the re-treatment period.

The reason biopsies are taken for the trial must be clearly and fully explained to the patient before they enter the trial, and consent re-checked with the patient at the relevant time prior to the biopsy to be taken.

If a patient refuses mandatory biopsy collection whilst on trial, the CTC should be contacted immediately for CI & TMG review.

Any measurable lesion should be **SEPARATE** from the biopsied lesion. The biopsied lesion should **NOT** be used for measuring response. If the original biopsiable lesion is no

DocuSign Envelope ID: F89E9DEB-F629-4294-B1D8-B570A4FACE07

longer present or no longer suitable for biopsy at 6 weeks then another site should be chosen. If no other biopsiable site then a 6 week biopsy is not possible and this should be documented.

Additional non-trial biopsies may also be performed if clinically indicated (eg, for mixed responses).

For patients requiring serial image-guided core needle tumour biopsy, those biopsies will be performed according to institutional practice.

If clinically practical, at each fresh biopsy collection time point, patients should undergo 4 core biopsies as per local hospital procedures. The first and third core biopsies will be placed in formalin and processed for formalin-fixed, paraffin-embedded blocks, while the second and fourth core biopsies will be immediately frozen in liquid nitrogen or equivalent method and then stored at between -70°C to -80°C. In exceptional cases, excisional or punch biopsies are permitted and may be substituted if sufficiently large (4 mm or greater in diameter).

Please also refer to the PEACOCC laboratory manual for further details.

DocuSign Envelope ID: F89E9DEB-F629-4294-B1D8-B570A4FACE07

12. DATA MANAGEMENT AND DATA HANDLING GUIDELINES

Data will be collected from sites on electronic case report forms (eCRFs) designed for the trial and supplied by UCL CTC. SAEs & AESIs will be reported on paper reports. Data must be accurately entered / transcribed onto trial forms / reports and must be verifiable from source data at site. Examples of source documents are hospital records which include patient's notes, laboratory and other clinical reports etc.

Where copies of supporting source documentation (e.g. autopsy reports, pathology reports, CT scan images etc.) are being submitted to UCL CTC, the patient's trial number must be clearly indicated on all material and any patient identifiers removed/blacked out prior to sending to maintain confidentiality.

12.1. Completing Electronic Case Report Forms (eCRFs)

All eCRFs must be entered by staff who are listed on the site staff delegation log and authorised by the PI to perform this duty. The PI is responsible for the accuracy of all data reported in the eCRF and paper SAE/AESI reports.

All entries must be clear, legible and written in ball point pen. Any corrections made to a paper SAE/AESI report at site must be made by drawing a single line through the incorrect item ensuring that the previous entry is not obscured. Each correction must be dated and initialed. Correction fluid must not be used.

The use of abbreviations and acronyms should be avoided.

Originals of the paper SAE/AESI reports must be sent to UCL and a copy kept at site.

12.2. Missing Data

To avoid the need for unnecessary data queries, eCRFs must be checked at site to ensure there are no blank mandatory fields that require completion (unless it is specifically stated that a field may be left blank). For paper SAE/AESI reports, when data are unavailable because a measure has not been taken or test not performed, enter "ND" for not done. If an item was not required at the particular time the form relates to, enter "NA" for not applicable. When data are unknown enter the value "NK" (only use if every effort has been made to obtain the data). For eCRF data entry guidance, please refer to the PEACOCC eCRF manual for sites which can be found in the ISF.

12.3. Timelines for Data Completion

Electronic CRFs must be completed at site as soon as possible after the relevant visit and within one month of the patient being seen.

Sites that persistently do not complete data items within the required timelines may be suspended from recruiting further patients into the trial by UCL CTC and subject to a 'Triggered' monitoring visit. See section 15.3 (Triggered' On-Site/Remote **Monitoring**)

DocuSign Envelope ID: F89E9DEB-F629-4294-B1D8-B570A4FACE07

for details. Please refer to section 13, for the reporting timeframes required for SAEs/AESI and AEs.

12.4. Data Queries

Data arriving at UCL CTC and entered onto the online eCRF database will be checked for completeness, accuracy, legibility and consistency, including checks for missing or unusual values. Data Clarification Requests will be sent / raised for the attention of the data / lead research contact at site. Further guidance on how data contacts should respond to data queries can be found on the Data Clarification Request forms. UCL CTC will send regular reports to sites, listing any overdue forms that require completion. Repeated failure to complete data queries / complete eCRFs within the required timeframes, will result in an escalation procedure that may involve suspension to recruitment at the trial site. Sites should inform UCL CTC ASAP of any issues they may experience that affects the completion of the data required.

DocuSign Envelope ID: F89E9DEB-F629-4294-B1D8-B570A4FACE07

13. PHARMACOVIGILANCE

13.1. Definitions

The following definitions have been adapted from the Medicines for Human Use (Clinical Trials) Regulations 2004 (SI 2004/1031) and subsequent amendments and ICH E2A "Clinical Safety Data Management: Definitions and Standards for Expedited Reporting" and ICH GCP E6.

Adverse Event (AE)

Any untoward medical occurrence in a patient treated on a trial protocol, which does not necessarily have a causal relationship with a trial treatment. An AE can therefore be any unfavourable and unintended sign (including an abnormal laboratory finding), symptom or disease temporally associated with the use of a trial treatment, whether or not related to that trial treatment. See section 13.2.1 for AE reporting procedures.

Adverse Reaction (AR)

All untoward and unintended responses to a trial treatment related to any dose administered. A causal relationship between a trial treatment and an adverse event is at least a reasonable possibility, i.e. the relationship cannot be ruled out.

Serious Adverse Event (SAE) or Serious Adverse Reaction (SAR)

An adverse event or adverse reaction that at any dose:

- Results in death.
- Is life threatening (the term "life-threatening" refers to an event in which the patient was at risk of death at the time of the event. It does not refer to an event that hypothetically might have caused death if it were more severe).
- Requires in-patient hospitalisation or prolongs existing hospitalisation.
- Results in persistent or significant disability/incapacity.
- Is a congenital anomaly or birth defect.
- Is otherwise medically significant (e.g. important medical events that may not be immediately life-threatening or result in death or hospitalisation but may jeopardise the patient or may require intervention to prevent one of the other outcomes listed above).

See section 13.2.2 for SAE reporting procedures.

Suspected Unexpected Serious Adverse Reaction (SUSAR)

An adverse event meeting the following criteria:

- Serious – meets one or more of the serious criteria above.
- Related – assessed by the local investigator or sponsor as causally related to one or more elements of the trial treatment.

DocuSign Envelope ID: F89E9DEB-F629-4294-B1D8-B570A4FACE07

- Unexpected – the event is not consistent with the applicable reference safety information (RSI).

See section 13.3 for reporting procedures for these events.

Adverse event of special interest (AESI)

An AE that is of special interest to the Trial Management Group MSD, even if does not meet the standard criteria for seriousness, or it occurs outside the standard AE reporting timeframes for the trial.

See section 13.4 for reporting procedures for these events.

13.2. Reporting Procedures

Adverse Event Term

An adverse event term must be provided for each adverse event. Wherever possible a valid term listed in the Common Terminology Criteria for Adverse Events (CTCAE) v5 should be used. This is available online at:

https://ctep.cancer.gov/protocoldevelopment/electronic_applications/ctc.htm#ctc_50

Severity grade

Severity grade of each adverse event must be determined by using CTCAE v5**.**

Causality

The relationship between the treatment and an adverse event will be assessed.

For AEs, the local PI or designee will assess whether the event is causally related to trial treatment.

For SAEs, a review will also be carried out by the Sponsor's delegate.

Causal relationship to trial treatment must be determined as follows:

- Related (reasonable possibility) to a trial treatment
- Not related (no reasonable possibility) to a trial treatment

UCL CTC will consider events evaluated as related to be adverse reactions.

13.2.1. Reporting of Adverse Events (AEs)

For registered patients who have received treatment, all adverse events that occur between informed consent and 30 days (or 110 for AESIs) post last trial treatment administration (see section 17.1 for end of trial definition) must be recorded in the patient notes and the trial eCRFs. Those meeting the definition of a Serious Adverse Event

DocuSign Envelope ID: F89E9DEB-F629-4294-B1D8-B570A4FACE07

(SAE) must also be reported to UCL CTC using the trial specific SAE Report. Also refer to section 13.2.2 (Reporting of Serious Adverse Events (SAEs)).

Pre-existing conditions (i.e. conditions present at informed consent) do not qualify as adverse events unless they worsen or recur (i.e. improves/resolves and then worsens/reappears again). For example, an AE could be an exacerbation of a chronic or intermittent pre-existing condition including either an increase in frequency and/or intensity of the condition (worsening of the event). Another example of an AE is when a pre-existing condition improves during the trial (e.g. from grade 3 to grade 1) and then it worsens again (e.g. from grade 1 to grade 2), even if the event is of severity equal or lower than the original condition (improvement and recurrence of the event).

N.B. the cancer under study and its anticipated day-to-day fluctuations would not be an AE.

13.2.2. Reporting of Serious Adverse Events (SAEs)

For registered patients who have received treatment, all SAEs that occur between the signing of informed consent and 110 days post last trial treatment administration **(or after this date if the site investigator feels the event is related to a trial treatment)** must be submitted to UCL CTC by fax / email within **24 hours** of observing or learning of the event, using the trial specific SAE Report. All sections on the SAE Report must be completed. If the event is **not being reported within 24 hours** to UCL CTC, the circumstances that led to this must be detailed in the SAE Report to avoid unnecessary queries.

For consented patients in screening and not registered to the trial or have been registered but not yet started trial treatment, those SAEs that occur between the signing of informed consent and 14 days post the last trial screening test must be submitted to UCL CTC by fax / email within 24 hours of observing or learning of the event, using the trial specific SAE Report.

Exemptions from SAE Report submission

For this trial, the following events are exempt from requiring submission on an SAE Report, but must be recorded in the relevant section(s) of the trial eCRFs:

- events that occur more than 110 days post last trial treatment administration unless:
  - considered to be a late effect of the trial treatment
  - it is a pregnancy related event (see section13.6)
- disease progression (including disease progression and disease related deaths) unless:
  - considered related to the IMP.

Please note that hospitalisation for elective treatment, palliative care or logistic/social reasons does not qualify as an SAE.

Completed SAE Reports must be faxed or emailed to UCL CTC within 24 hours of becoming aware of the event

Fax: +44 (0)20 7679 9871
Email: ctc.peacocc@ucl.ac.uk

SAE Follow-Up Reports

All SAEs must be followed-up until resolution and until there are no further queries. Sites must ensure any new and relevant information is provided promptly. If the event term changes or a new event is added, the causality must be re-assessed by an Investigator. If the event is not being reported **within 24 hours** to UCL CTC, the circumstances that led to this must be detailed in the SAE/SAR Report to avoid unnecessary queries.

Adverse Event Reporting Flowchart on the following page.

DocuSign Envelope ID: F89E9DEB-F629-4294-B1D8-B570A4FACE07

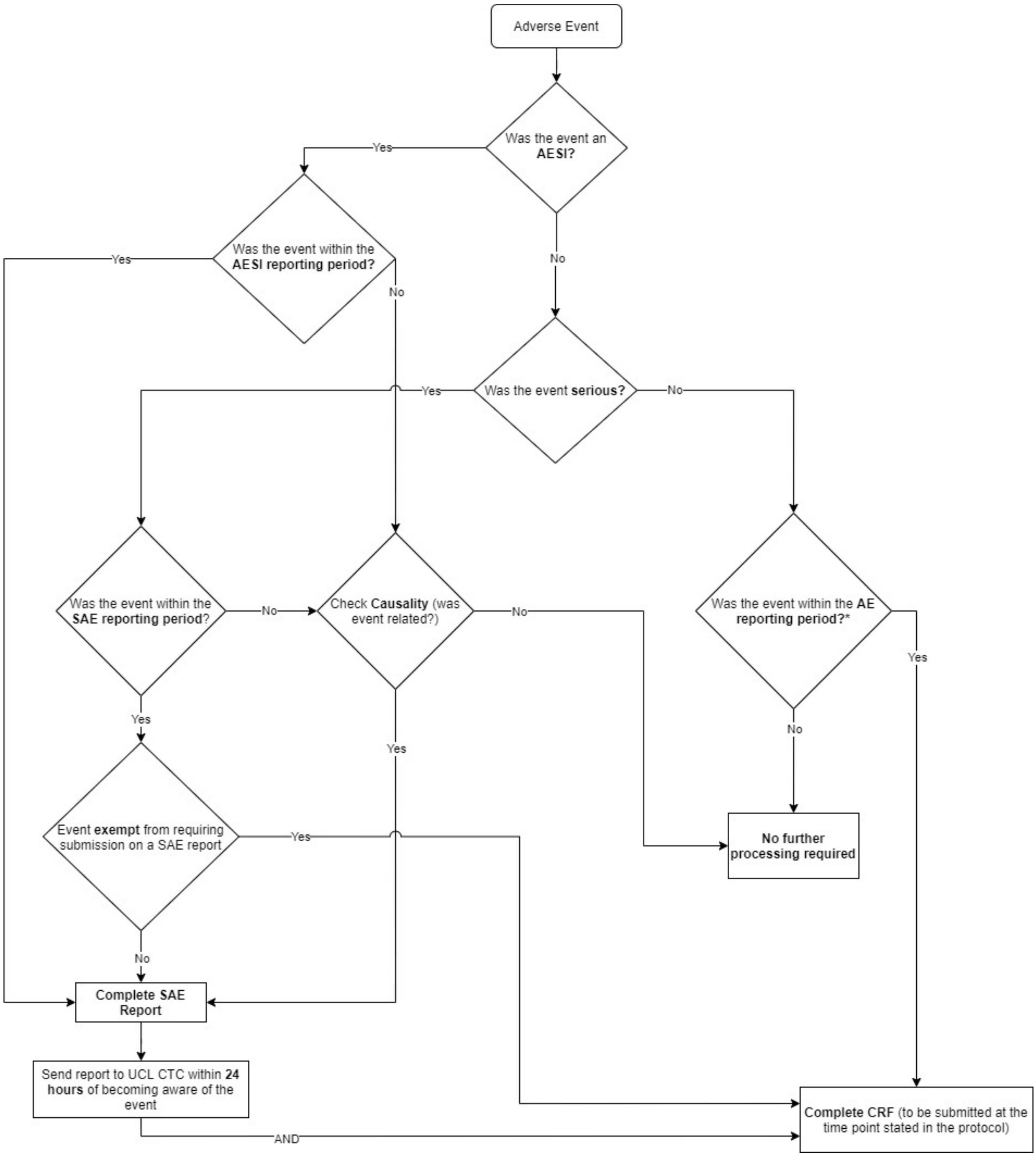

** This applies if AE, SAE and AESI reporting period differs*

SAE Processing at UCL CTC

On receipt of the SAE Report, UCL CTC will check for legibility, completeness, accuracy and consistency. UCL CTC will evaluate expectedness, to determine whether or not the case qualifies for expedited reporting, using the RSI (the list of expected adverse events in the approved IB for pembrolizumab).

The CI, or their delegate (e.g. a clinical member of the TMG), will be contacted to review the SAE and to perform an evaluation of causality on behalf of UCL CTC. If UCL CTC has considered expectedness difficult to determine, the CI, or their delegate, will be consulted for their opinion at this time.

UCL CTC will submit all SAE Reports concerning patients who have received pembrolizumab to MSD according to the timelines outlined in the agreement between UCL and MSD.

13.3. SUSARs

If the event is evaluated as a SUSAR, i.e. an unexpected event that is related (reasonable possibility) to the investigational drug, UCL CTC will submit a report to the MHRA and the REC within 7 calendar days for initial reports of fatal/life threatening events (with a follow-up report within a further 8 calendar days) and 15 calendar days for all other events.Wherever possible, evaluations of causal relationship by both the site and the Sponsor's clinical reviewer will be reported.

UCL CTC will submit all SUSAR reports relating to pembrolizumab to MSD according to the timelines outlined in the agreement between UCL and MSD.

Informing Sites of SUSARs

UCL CTC will inform all PIs of any SUSARs that occur on the trial. PIs will receive a quarterly line listing which must be processed according to local requirements.

UCL CTC will forward any reports received from MSD regarding SUSARs that have occurred on other trials using pembrolizumab to all PIs. These must be processed according to local requirements and filed with the applicable IB.

13.4. Adverse events of special interest

The following adverse events of special interest for pembrolizumab must be reported on an SAE report within **24 hours of becoming aware of the event** between the signing of informed consent and 110 days after the date of last IMP administration**:**

- An overdose of Pembrolizumab, as defined in Section 9.6
- Abnormal liver function tests meeting ALL the following criteria:
    - an elevated AST or ALT lab value that is greater than or equal to 3X the upper limit of normal **AND**
    - an elevated total bilirubin lab value that is greater than or equal to 2X the upper limit of normal **AND**,
    - an alkaline phosphatase lab value that is less than 2 x the upper limit of normal, as determined by way of protocol-specified laboratory testing or unscheduled laboratory testing.*

*Note: These criteria are based upon available regulatory guidance documents. The purpose of the criteria is to specify a threshold of abnormal hepatic tests that may require an additional evaluation for an underlying aetiology.

All AEs of special interest must be reported by faxing a completed SAE report to UCL CTC within 24 hours of becoming aware of the event
Fax: +44 (0)20 7679 9871
Email: ctc.peacocc@ucl.ac.uk

13.5. Safety Monitoring

UCL CTC will provide safety information to the Trial Management Group (TMG) and the Independent Data Monitoring Committee (IDMC) on a periodic basis for review.

Trial safety data will be monitored to identify:

- new adverse reactions to the trial treatment regimen or individual trial treatments
- a higher incidence in rare adverse events than is stated in the IB for a trial treatment
- trial related events that are not considered related to the trial treatment regimen

If UCL CTC identifies or suspects any issues concerning patient safety at any point during the trial, the CI or TMG will be consulted for their opinion, and if necessary the issue will be referred to the IDMC.

13.6. Pregnancy

Reporting Period

If a female patient becomes pregnant between the start of trial treatment and 120 days after last trial treatment administration (or 30 days following cessation of treatment if the patient initiates a new anticancer therapy, whichever is earlier) the site must submit a trial specific Pregnancy Report to UCL CTC by fax or email **within 24 hours** of learning of its occurrence.

The site must request consent from the pregnant trial patient to report information regarding a pregnancy using:

- The trial-specific Pregnancy Monitoring Information Sheet and Informed Consent Form for trial patients

If consent is not given, the notification that a pregnancy has occurred will be retained by UCL CTC, however no further action will be taken on the information detailed in the report.

All pregnancies must be reported by faxing or emailing a completed Pregnancy Report to UCL CTC within 24 hours of becoming aware of the pregnancy
Fax: +44 (0)20 7679 9871
Email: ctc.peacocc@ucl.ac.uk

Pregnancy Follow-Up Reports

Pregnant patients who consent to pregnancy follow-up, must have their pregnancies followed -up at least monthly for up to 6 weeks after the end of the pregnancy (or later if there are ongoing issues) to collect information on any ante- and post-natal problems for both mother and child. If significant new information is received, follow-up Pregnancy Reports must be submitted to UCL CTC by fax or email within 24 hours of learning of the information. In the case of an adverse outcome to the pregnancy (e.g. miscarriage), reports must include an evaluation of the possible relationship between the trial treatment and the pregnancy outcome. SAEs during pregnancy

Any SAE occurring in a pregnant patient must be reported using the trial specific SAE Report, according to SAE reporting procedures. Refer to section 13.2.2 (Reporting of Serious Adverse Events (SAEs)) for details.

Pregnancy Report processing at UCL CTC

UCL CTC will submit a report to the MHRA and the REC if the pregnancy outcome meets the definition of a SUSAR. Refer to section 13.3 (SUSARs) for details.

UCL CTC will submit all Pregnancy Reports concerning exposure to pembrolizumab to MSD according to the timelines outlined in the agreement between UCL and MSD.

13.7. Development Safety Update Reports (DSURs)

Safety data obtained from the trial will be included in DSURs that UCL CTC will submit to the MHRA and the REC.

UCL CTC will provide MSD with DSURs that include information regarding pembrolizumab.

DocuSign Envelope ID: F89E9DEB-F629-4294-B1D8-B570A4FACE07

14. INCIDENT REPORTING AND SERIOUS BREACHES

14.1. Incident Reporting

Organisations must notify UCL CTC of all deviations from the protocol or GCP immediately. An incident report may be requested and will be provided, but an equivalent document (e.g. Trust Incident form) is acceptable where already completed. Where an equivalent document is being submitted to UCL CTC, the patient's trial number must be clearly indicated on all material and any patient identifiers redacted prior to sending, to maintain confidentiality.

If site staff are unsure whether a certain occurrence constitutes a deviation from the protocol or GCP, the UCL CTC trial team can be contacted immediately to discuss.

UCL CTC will use an organisation's history of non-compliance to make decisions on future collaborations.

UCL CTC will assess all incidents to see if they meet the definition of a serious breach.

14.2. Serious Breaches

A "serious breach" is defined as a breach of the protocol or of the conditions or principles of Good Clinical Practice which is likely to affect to a significant degree the safety or physical or mental integrity of the trials, or the scientific value of the research.

Systematic or persistent non-compliance by a site with GCP and/or the protocol, including failure to report SAEs occurring on trial within the specified timeframe, may be deemed a serious breach.

In cases where a serious breach has been identified, UCL CTC will inform the MHRA and REC within 7 calendar days of becoming aware of the breach.

Sites must have written procedures for notifying the sponsor of serious breaches (MHRA Guidance on the Notification of Serious Breaches).

DocuSign Envelope ID: F89E9DEB-F629-4294-B1D8-B570A4FACE07

15. TRIAL MONITORING AND OVERSIGHT

Participating sites and PIs must agree to allow trial-related on-site monitoring, Sponsor audits and regulatory inspections by providing direct access to source data/documents as required. Where permitted by site policy, remote access to source data/documents may also be provided by participating sites for remote monitoring by UCL CTC or its representatives.

Patients are informed of this in the patient information sheet and are asked to consent to their medical notes being reviewed by appropriate individuals on the consent form. UCL CTC or its representatives will conduct all monitoring in compliance with the participant consent, site policy and data protection requirements.

UCL CTC will determine the appropriate level and nature of monitoring required based on the objective, purpose, phase, design, size, complexity, endpoints and risks associated with for the trial. Risk will be assessed on an ongoing basis and adjustments made accordingly.

Details of monitoring activities will be included in the trial monitoring plan and conveyed to sites during initiation. The Monitoring Plan will be kept under review during the trial and updated information provided to sites as necessary.

15.1. On-Site and Remote Monitoring

On-site Monitoring

Sites will be sent an email in advance of any on-site monitoring visits, confirming when a visit is scheduled to take place. The email will include a list of the documents to be reviewed, interviews that will be conducted, planned inspections of the facilities and who will be performing the visit.

Remote Monitoring

UCL CTC defines remote monitoring as activities conducted at a location remote from the research site which replicate some on-site activities e.g. source data review. Remote monitoring may be conducted in response to exceptional circumstances preventing access to participating sites (e.g. global pandemic) or conducted routinely. Details of remote monitoring will be agreed with participating sites, conducted in accordance with site policy and documented in the monitoring plan.

Sites will be sent an email in advance, confirming when remote monitoring is scheduled to take place and how the source documents will be remotely accessed. The email will include a list of the documents to be reviewed, interviews that will be conducted via telephone/videoconference and who will be performing the remote monitoring.

Remote monitoring will be conducted by UCL CTC or its representatives via a device with adequate security. Patient confidentiality will be maintained at all times, and monitoring activities will be conducted in an appropriate environment where no unauthorised viewing or overhearing of conversations is possible by third parties. Refer to section 12 Data Management and Data Handling Guidelines for details of how source documentation should be submitted to UCL CTC.

DocuSign Envelope ID: F89E9DEB-F629-4294-B1D8-B570A4FACE07

Monitoring Follow Up

Following on-site remote monitoring, the Trial Monitor/Trial Coordinator will provide a follow up email to the site, which will summarise the documents reviewed and a statement of findings, incidents, deficiencies, conclusions, actions taken and/or actions required. The PI at each site will be responsible for ensuring that monitoring findings are addressed in a timely manner, and by the deadline specified.

15.2. Centralised Monitoring

UCL CTC performs centralised monitoring, which requires the submission of the following documents by sites to UCL CTC for review: screening logs, staff delegation logs and accountability logs. Expectations for document submission will be explained during site initiation and UCL CTC or its representatives will send emails to sites requesting the documents when required.

Sites will be requested to conduct quality control checks of documentation held within the Investigator Site File and Pharmacy Site File at the frequency determined for the trial. Checklists detailing the current version/date of version controlled documents will be provided by UCL CTC for this purpose.

15.3. Triggered' On-Site/Remote Monitoring

Additional on-site or remote monitoring visits may be scheduled following UCL CTC review and/or where there is evidence or suspicion of non-compliance at a site with important aspect(s) of the trial protocol/GCP requirements.

On-site Monitoring

Sites will be sent an email in advance outlining the reason(s) for the visit and confirming when it will take place. The email will include a list of the documents that are to be reviewed, interviews that will be conducted, planned inspections of the facilities and who will be performing the visit.

Remote Monitoring

Sites will be sent an email in advance, confirming when remote monitoring is scheduled to take place and how the source documents will be remotely accessed. The email will include a list of the documents to be reviewed, interviews that will be conducted via telephone/videoconference and who will be performing remote monitoring.

15.4. Escalation of monitoring issues

Where monitoring indicates that a patient may have been placed at risk (e.g. evidence of an overdose having been administered, indication that treatment discontinuation rules were not observed following an adverse reaction, etc), the matter will be raised urgently with site staff and escalated as appropriate.

UCL CTC will assess whether it is appropriate for the site to continue participation in the trial and whether the incident(s) constitute a serious breach. Refer to section for details.

15.5. Oversight Committees

15.5.1. Trial Management Group (TMG)

The TMG will include the Chief Investigator, clinicians and experts from relevant specialties, PEACOCC trial staff from UCL CTC and a patient representative (see page 2). The TMG will be responsible for overseeing the trial. The group will meet regularly and will send updates to PIs (via newsletters or at Investigator meetings) and to the NCRI Gynaecological Clinical Studies Group.

The TMG will review substantial amendments to the protocol prior to submission to the REC and MHRA. All PIs will be kept informed of substantial amendments through their nominated responsible individual and are responsible for their prompt implementation.

All TMG members will be required to sign a UCL CTC TMG Charter.

15.5.2. Trial Steering Committee (TSC)

The role of the TSC is to provide overall supervision of the trial. The TSC will review the recommendations of the Independent Data Monitoring Committee and, on consideration of this information, recommend any appropriate amendments/actions for the trial as necessary. The TSC acts on behalf of the funder and the Sponsor.

All TSC members will be required to sign a UCL CTC TSC Charter.

15.5.3. Independent Data Monitoring Committee (IDMC)

The role of the IDMC is to provide independent advice on data and safety aspects of the trial. Meetings of the Committee will be held at least annually to review interim analyses or as necessary to address any issues. The IDMC is advisory to the TSC and can recommend premature closure of the trial to the TSC.

All IDMC members will be required to sign a UCL CTC Charter.

15.5.4. Role of UCL CTC

UCL CTC, on behalf of the Sponosr (UCL) will be responsible for the day to day coordination and management of the trial and the UCL CTC Director will act as custodian of the data generated in the trial (on behalf of UCL). UCL CTC is responsible for all duties

DocuSign Envelope ID: F89E9DEB-F629-4294-B1D8-B570A4FACE07

relating to pharmacovigilance which are conducted in accordance with section 13 (Pharmacovigilance).

DocuSign Envelope ID: F89E9DEB-F629-4294-B1D8-B570A4FACE07

16. WITHDRAWAL OF PATIENTS

In consenting to the trial, patients are consenting to trial treatment, assessments, collection of biological samples, follow-up and data collection.

16.1. Patient Withdrawal/Discontinuation Criteria

A patient may be withdrawn from the trial or trial treatment whenever such treatment is no longer in the patient's best interests. The reasons for withdrawal from treatment must be recorded in the patient's notes and on the relevant Case Report Form(s).

Reasons for discontinuing treatment may include:

- Patient's decision to discontinue treatment.
- Confirmed radiographic disease progression
  Note: For unconfirmed radiographic disease progression, please see Section 16.1.2.
- Unacceptable adverse events as described in Section 5.
- Intercurrent illness that prevents further administration of treatment.
- Investigator's decision to withdraw the patient from treatment.
- The patient has a confirmed positive urine or serum pregnancy test or fails to use adequate contraceptive methods.
- Non-compliance with trial treatment or procedure requirements.
- The patient is lost to follow-up.
- Completed 24 months of uninterrupted treatment with pembrolizumab or 35 administrations of study medication, whichever is later.
  Note: 24 months of study medication is calculated from the date of first dose. Patients who stop pembrolizumab after 24 months may be eligible for up to one year of additional study treatment if they progress after stopping study treatment provided they meet the requirements detailed in Section 9.3.2.

After the end of treatment, each patient will be followed up for safety monitoring as per Section 13.2.2. The end of treatment and follow-up visit procedures are listed in Appendix 1 (Trial Schedule of Procedures/Assessments) and Section 10.4.2 (Visit Requirements).

If a patient expresses their wish to withdraw from the trial either during treatment or while in follow-up, sites should explain the importance of remaining on trial follow-up, or failing this of allowing routine follow-up data to be used for trial purposes and for allowing existing collected data to be used. If the patient gives a reason for their withdrawal, this should be recorded on the relevant eCRF(Change of Status).

16.1.1. Discontinuation of Study Treatment after CR

Discontinuation of treatment may be considered for patients who have attained a confirmed CR that have been treated for at least 24 weeks with pembrolizumab and had

DocuSign Envelope ID: F89E9DEB-F629-4294-B1D8-B570A4FACE07

at least two treatments with pembrolizumab beyond the date when the initial CR was declared. Patients who then experience radiographic disease progression may be eligible for up to one year of additional treatment with pembrolizumab via the re-treatment period at the discretion of the investigator if no cancer treatment was administered since the last dose of pembrolizumab, the patient meets the safety parameters listed in the Inclusion/Exclusion criteria, and the trial is open. Patients will resume therapy at the same dose and schedule at the time of initial discontinuation. Additional details are provided in Section 9.3.3.

16.1.2. Treatment beyond Equivocal Progression

If unconfirmed progressive disease (PD; based on RECIST v1.1) occurs before completion of the 24-month treatment period, the patient may continue to be treated until one of the following criteria is met:

- Confirmation of PD: The assessment of unconfirmed PD by RECIST v1.1 (baseline PD assessment) will be confirmed by a repeat evaluation at the next tumour assessment time point, but no sooner than 4 weeks later. If this confirms unequivocal progressive disease the patient should discontinue trial treatment.
- Meets any of the investigational product discontinuation criteria (Section 16.1)
- Clinical symptoms or signs indicating clinically significant PD such as the benefit-risk ratio of continuing therapy is no longer justified.
- Decline in Eastern Cooperative Oncology Group (ECOG) performance status compared to baseline.
- Rapid PD or threat to vital organs/critical anatomical sites (eg, spinal cord compression) requiring urgent alternative medical intervention, and/or continuation of study therapy would prevent institution of such intervention.

16.2. Future Data Collection

If a patient explicitly states they do not wish to contribute further data to the trial their decision must be respected, with the exception of essential safety data, and recorded on the relevant eCRF (Change of Status). In this event data due up to the date of withdrawal must be submitted but no further data, other than essential safety data, sent to UCL CTC.

16.3. Losses to Follow-Up

If a patient moves from the area, every effort should be made for the patient to be followed up at another participating trial site and for this new site to take over the responsibility for the patient, or for follow-up via GP. Details of participating trial sites can be obtained from the UCL CTC trial team, who must be informed of the transfer of care and follow up arrangements. If it is not possible to transfer to a participating site, the registering site remains responsible for submission of forms.

DocuSign Envelope ID: F89E9DEB-F629-4294-B1D8-B570A4FACE07

If a patient is lost to follow-up at a site every effort should be made to contact the patient's GP to obtain information on the patient's status.

DocuSign Envelope ID: F89E9DEB-F629-4294-B1D8-B570A4FACE07

17. TRIAL CLOSURE

17.1. End of Trial

For regulatory purposes the end of the trial will be when the last patient alive who has completed initial and re-treatment (if applicable) has two follow-up/survival follow-up assessments (either prior to or after progression) or dies, whichever occurs first and once all protocol planned exploratory research has been completed. At which point the 'declaration of end of trial' form will be submitted to the MHRA and Ethics Committee, as required.

Following this, UCL CTC will advise sites on the procedure for closing the trial at the site.

Once the end of trial has been declared, no more prospective patient data will be collected but sites must co-operate with any data queries regarding existing data to allow for analysis and publication of results.

17.2. Archiving of Trial Documentation

At the end of the trial, UCL CTC will archive securely all centrally held trial related documentation for a minimum of 25 years. Arrangements for confidential destruction will then be made. It is the responsibility of PIs to ensure data and all essential documents relating to the trial held at site are retained securely for a minimum of 25 years after the end of the trial, and in accordance with national legislation.

Essential documents are those which enable both the conduct of the trial and the quality of the data produced to be evaluated and show whether the site complied with the principles of GCP and all applicable regulatory requirements.

UCL CTC will notify sites when trial documentation held at sites may be archived. All archived documents must continue to be available for inspection by appropriate authorities upon request.

17.3. Early Discontinuation of Trial

The trial may be stopped before completion as an Urgent Safety Measure on the recommendation of the TSC or IDMC (see section 15.5.2 Trial Steering Committee (TSC) and 15.5.3 Independent Data Monitoring Committee (IDMC)). Sites will be informed in writing by UCL CTC of reasons for early closure and the actions to be taken with regards the treatment and follow up of patients.

17.4. Withdrawal from Trial Participation by a Site

Should a site choose to close to recruitment the PI must inform UCL CTC in writing. Follow up as per protocol must continue for any patients recruited into the trial at that site and other responsibilities continue as per the Site Agreement.

DocuSign Envelope ID: F89E9DEB-F629-4294-B1D8-B570A4FACE07

18. STATISTICS

18.1. Sample Size Calculation

Assuming that the proportion of progression-free patients at 12 weeks will be 33%, a proportion <15% would be of no further interest [16]. Using A'hern's single-stage phase II design with 90% power and one-sided 5% significance level, a sample size of 48 patients is required with ≥12 patients alive and progression-free at 12 weeks to warrant further investigation. The sample size was calculated using "Sample size tables for clinical studies" software.

18.2. Statistical analysis

18.2.1. Analysis of main endpoint

The number and percentage of progression-free patients at 12 weeks will be presented, with 90% two-sided confidence intervals for the percentage.

The proportion of progression-free patients is defined as the number of patients alive with complete or partial response or stable disease maintained at 12 weeks (as assessed by the site radiologist and/or investigator, using RECIST v1.1) divided by the number of patients in the analysis population. Only patients with tumour assessment at or beyond 12 weeks will be considered progression-free.

All eligible patients who received at least one cycle of pembrolizumab will be included in the analysis population (i.e. safety population).

Any patient found to be ineligible will be excluded and replaced.

18.2.2. Analysis of secondary endpoints and secondary analyses

18.2.2.1. Progression-free survival

Progression-free survival is defined as the time from start of treatment to progression or death from any cause (whichever comes first). All deaths will be included, whether they occur on study or following treatment discontinuation. For patients who have not died or progressed, progression-free survival will be censored at the date of last contact.

Kaplan-Meier survival methods will be used.

18.2.2.2. Time to second disease progression (Re-treatment period)

Time to second disease progression refers to progression in those patients re-treated on trial after their first progression. It is defined as the time from diagnosis of first progression during or after their initial pembrolizumab therapy to second progression or death from any cause (whichever comes first), during or after their retreatment pembrolizumab. All deaths will be included, whether they occur during the re-treatment period or following

DocuSign Envelope ID: F89E9DEB-F629-4294-B1D8-B570A4FACE07

treatment discontinuation. For patients who have not died or progressed, second progression-free survival will be censored at the date of last contact.

Kaplan-Meier survival methods will be used.

18.2.2.3. Overall survival

Overall survival is defined as the time from start of treatment to death from any cause. All deaths will be included, whether they occur on study or following treatment discontinuation. For patients who have not died, overall survival will be censored at the date of last contact.

Kaplan-Meier survival methods will be used.

18.2.2.4. Objective response at 12 weeks and best objective response

Objective response is defined as a complete or partial response as determined by RECIST v1.1. Patients without a tumour assessment at 12 weeks will be considered to be non-responders. The proportion of patients with objective response at 12 weeks will be presented as a proportion with 90% two-sided confidence interval.

The best objective response consists of the best among all objective responses assessed. Best objective response will be reported as a proportion with 90% two-sided confidence interval. The analysis of best objective response will be performed with all patients over the whole study period (including Re-treatment phase). A subset analysis will be performed for patients in the Re-treatment phase, once over the whole study period and once over the Re-treatment phase only.

18.2.2.5. Duration of Response

For patients with an objective response, duration of objective response (DoR) is defined as the time from initial complete or partial response to disease progression or death on study from any cause (defined as death within 110 days of the last study treatment), whichever occurs first.

For patients with disease control (objective response or stable disease maintained ≥12 weeks), duration of disease control (DoC) is defined as the time from start of treatment to disease progression or death on study from any cause (defined as death within 30 days of the last study treatment), whichever occurs first.

A subset analysis of DoR and DoC will be performed only in patients re-treated. This analysis will be performed once over the whole study period and once over the Re-treatment phase only.

Kaplan-Meier survival methods will be used, and patients will be censored at the date of last contact.

18.2.2.6. Quality of Life

The questionnaire used will be FACT-O (version 4). QoL data will be analysed using methods for repeated measures. Descriptive statistics will be presented at each time point, as well as changes from baseline, for each subscale and overall. The algorithm for core construction will be based on that provided by the FACT-O manual.

18.2.2.7. Safety Monitoring

All patients who received at least one cycle of pembrolizumab will be included in the safety analysis. Adverse events (AEs) will be monitored on an ongoing basis and their frequencies reported. AEs will be categorized using the NCI Common Terminology Criteria for Adverse Events (CTCAE), version 5. The worst event for each patient will be described. Both events related and unrelated to treatment will be captured.

Clinical and laboratory data will be tabulated and compared to normal ranges for the institution.

18.3. Interim analyses

The study will be regularly monitored by UCL CTC, with input from members of the Trial Management Group. A report will be provided to the Independent Data Monitoring Committee (IDMC), who will review accrual, compliance, safety and efficacy. The first review by the IDMC will be triggered after 15 patients have completed the first CT scan assessment following the first cycle of pembrolizumab, and at least once each year thereafter. The IDMC will make recommendations on whether the trial should continue or stop recruitment, or the protocol modified. Any recommendation to stop the trial will be communicated to Trial Steering Committee (TSC).

19. ETHICAL AND REGULATORY CONSIDERATIONS

In conducting the trial, the Sponsor, UCL CTC and sites shall comply with all relevant guidance, laws and statutes, as amended from time to time, applicable to the performance of clinical trials including, but not limited to:

- The principles of ICH Harmonised Tripartite Guideline for Good Clinical Practice as set out in Schedule 1 (Conditions and Principles of Good Clinical Practice and for the Protection of Clinical Trial Patients) of the Medicines for Human Use (Clinical Trials) Regulations 2004 and the GCP Directive 2005/28/EC, as set out in SI 2006/1928.
- Human Rights Act 1998.
- Data Protection Act 2018 and General Data Protection Regulation (EU)2016/679 (GDPR).
- Freedom of Information Act 2000.
- Human Tissue Act 2004.
- Medicines Act 1968.
- Medicines for Human Use (Clinical Trials) UK Regulations SI 2004/1031, and subsequent amendments.
- Good Manufacturing Practice.
- UK Policy Framework for Health and Social Care Research, issued by the Health Research Authority
- The Human Medicines (Amendment etc.) (EU Exit) Regulations 2019 (SI 2019/775)

19.1. Ethical Approval

The trial will be conducted in accordance with the World Medical Association Declaration of Helsinki entitled ‘Ethical Principles for Medical Research Involving Human Patients’ (1996 version) and in accordance with the terms and conditions of the ethical approval given to the trial.

The trial has received a favourable opinion from the Yorkshire & The Humber - Sheffield Research Ethics Committee (REC) and Health Research Authority (HRA) approval for conduct in the UK.

UCL CTC will submit Annual Progress Reports to the REC, commencing one year from the date of ethical approval for the trial.

19.2. Regulatory Approval

A Clinical Trial Authorisation (CTA) has been granted for the trial.

The trial will be conducted at approved trial sites in accordance with the trial protocol and the terms of the CTA granted by the MHRA.

DocuSign Envelope ID: F89E9DEB-F629-4294-B1D8-B570A4FACE07

19.3. Site Approvals / Confirmation of Capacity & Capability

Evidence of assessment of capability and capacity or local approvals by the Trust/Health Board R&D for a trial site must be provided to UCL CTC. Sites will only be activated when all necessary local approvals for the trial have been obtained.

19.4. Protocol Amendments

UCL CTC will be responsible for gaining ethical and regulatory approval(s), as appropriate, for amendments made to the protocol and other trial-related documents. Once approved, UCL CTC will ensure that all amended documents are distributed to sites as appropriate.

Site staff will be responsible for acknowledging receipt of documents and for implementing all amendments promptly.

19.5. Patient Confidentiality & Data Protection

Patient identifiable data, including initials and date of birth, will be collected for the trial by UCL CTC. UCL CTC will preserve patient confidentiality and will not disclose or reproduce any information by which patients could be identified in public reports or results. Data will be stored in a secure manner and UCL CTC trials are registered in accordance with the Data Protection Act 2018 and GDPR with the Data Protection Officer at UCL.

DocuSign Envelope ID: F89E9DEB-F629-4294-B1D8-B570A4FACE07

20. SPONSORSHIP AND INDEMNITY

20.1. Sponsor Details

Sponsor Name:	University College London
Address:	Joint Research Office<br>4th Floor - West<br>250 Euston Road<br>London<br>NW1 2PG
Contact:	Managing Director, UCLH/UCL Research
Tel:<br>Fax:	020 3447 9995/2178 (unit admin)<br>020 3447 9937

20.2. Indemnity

University College London holds insurance to cover participants for injury caused by their participation in the clinical trial. Participants may be able to claim compensation if they can prove that UCL has been negligent. However, as this clinical trial is being carried out in a hospital, the hospital continues to have a duty of care to the participant of the clinical trial. University College London does not accept liability for any breach in the hospital's duty of care, or any negligence on the part of hospital employees. This applies whether the hospital is an NHS Trust or otherwise.

Participants may also be able to claim compensation for injury caused by participation in this clinical trial without the need to prove negligence on the part of University College London or another party. Participants who sustain injury and wish to make a claim for compensation should do so in writing in the first instance to the Chief Investigator, who will pass the claim to the Sponsor's Insurers, via the Sponsor's office.

Hospitals selected to participate in this clinical trial shall provide clinical negligence insurance cover for harm caused by their employees and a copy of the relevant insurance policy or summary shall be provided to University College London, upon request.

DocuSign Envelope ID: F89E9DEB-F629-4294-B1D8-B570A4FACE07

21. FUNDING

MSD are supporting the central coordination of the trial in the UK through UCL CTC and providing the study IMP, pembrolizumab.

There is a contribution for Research A and B costs which will be paid to sites as per the finance section of the Site Agreement.

DocuSign Envelope ID: F89E9DEB-F629-4294-B1D8-B570A4FACE07

22. PUBLICATION POLICY

All publications and presentations relating to the trial will be authorised by the Trial Management Group. The first publication of the trial results will be in the name of the Trial Management Group. Members of the TMG and other investigators that the TMG deem appropriate will be included as named authors. Data from all sites will be analysed together and published as soon as possible. Authorship will be agreed in advance between the investigators, based on the number of patients recruited at each centre and contribution to the initiation and conduct of the trial and analysis of data. Participating sites may not publish trial results prior to the first publication by the TMG or without prior written consent from the TMG. The trial data is owned by UCL as Sponsor. The ClinicalTrials.gov number NCT03425565 and funder reference will be quoted in any publications resulting from this trial

Abstracts and papers will be reviewed by MSD prior to submission in accordance with the requirements of the Trial Drug Supply Agreement.

DocuSign Envelope ID: F89E9DEB-F629-4294-B1D8-B570A4FACE07

23. REFERENCES

1. Hasegawa K, Nagao S, Yasuda M et al. Gynecologic Cancer InterGroup (GCIG) consensus review for clear cell carcinoma of the uterine corpus and cervix. Int J Gynecol Cancer 2014; 24: S90-95.
2. Shu CA, Zhou Q, Jotwani AR et al. Ovarian clear cell carcinoma, outcomes by stage: The MSK experience. Gynecol Oncol 2015; 139: 236-241.
3. Creasman WT, Phillips JL, Menck HR. The National Cancer Data Base report on cancer of the vagina. Cancer 1998; 83: 1033-1040.
4. Glasspool RM, McNeish IA. Clear cell carcinoma of ovary and uterus. Curr Oncol Rep 2013; 15: 566-572.
5. Kurman RJ, Shih Ie M. Molecular pathogenesis and extraovarian origin of epithelial ovarian cancer--shifting the paradigm. Hum Pathol 2011; 42: 918-931.
6. Zorn KK, Bonome T, Gangi L et al. Gene expression profiles of serous, endometrioid, and clear cell subtypes of ovarian and endometrial cancer. Clin Cancer Res 2005; 11: 6422-6430.
7. Ikeda Y, Oda K, Aburatani H et al. Non-diethylstilbestrol exposed vaginal clear cell adenocarcinoma has a common molecular profile with ovarian clear cell adenocarcinoma: A case report. Gynecol Oncol Rep 2014; 10: 49-52.
8. Despierre E, Yesilyurt BT, Lambrechts S et al. Epithelial ovarian cancer: rationale for changing the one-fits-all standard treatment regimen to subtype-specific treatment. Int J Gynecol Cancer 2014; 24: 468-477.
9. Fata CR, Seeley EH, Desouki MM et al. Are clear cell carcinomas of the ovary and endometrium phenotypically identical? A proteomic analysis. Hum Pathol 2015; 46: 1427-1436.
10. Chan JK, Teoh D, Hu JM et al. Do clear cell ovarian carcinomas have poorer prognosis compared to other epithelial cell types? A study of 1411 clear cell ovarian cancers. Gynecol Oncol 2008; 109: 370-376.
11. Sung PL, Chang YH, Chao KC et al. Global distribution pattern of histological subtypes of epithelial ovarian cancer: a database analysis and systematic review. Gynecol Oncol 2014; 133: 147-154.
12. Anuradha S, Webb PM, Blomfield P et al. Survival of Australian women with invasive epithelial ovarian cancer: a population-based study. Med J Aust 2014; 201: 283-288.
13. Mackay HJ, Brady MF, Oza AM et al. Prognostic relevance of uncommon ovarian histology in women with stage III/IV epithelial ovarian cancer. Int J Gynecol Cancer 2010; 20: 945-952.
14. Miyamoto M, Takano M, Goto T et al. Clear cell histology as a poor prognostic factor for advanced epithelial ovarian cancer: a single institutional case series through central pathologic review. J Gynecol Oncol 2013; 24: 37-43.
15. Pather S, Quinn MA. Clear-cell cancer of the ovary-is it chemosensitive? Int J Gynecol Cancer 2005; 15: 432-437.
16. Takano M, Sugiyama T, Yaegashi N et al. Low response rate of second-line chemotherapy for recurrent or refractory clear cell carcinoma of the ovary: a retrospective Japan Clear Cell Carcinoma Study. Int J Gynecol Cancer 2008; 18: 937-942.

DocuSign Envelope ID: F89E9DEB-F629-4294-B1D8-B570A4FACE07

17. Hamanishi J, Mandai M, Iwasaki M et al. Programmed cell death 1 ligand 1 and tumor-infiltrating CD8+ T lymphocytes are prognostic factors of human ovarian cancer. Proc Natl Acad Sci U S A 2007; 104: 3360-3365.
18. Matsuzaki J, Gnjatic S, Mhawech-Fauceglia P et al. Tumor-infiltrating NY-ESO-1-specific CD8+ T cells are negatively regulated by LAG-3 and PD-1 in human ovarian cancer. Proc Natl Acad Sci U S A 2010; 107: 7875-7880.
19. Zhang L, Conejo-Garcia JR, Katsaros D et al. Intratumoral T cells, recurrence, and survival in epithelial ovarian cancer. N Engl J Med 2003; 348: 203-213.
20. Abiko K, Mandai M, Hamanishi J et al. PD-L1 on tumor cells is induced in ascites and promotes peritoneal dissemination of ovarian cancer through CTL dysfunction. Clin Cancer Res 2013; 19: 1363-1374.
21. Duraiswamy J, Freeman GJ, Coukos G. Therapeutic PD-1 pathway blockade augments with other modalities of immunotherapy T-cell function to prevent immune decline in ovarian cancer. Cancer Res 2013; 73: 6900-6912.
22. Debernardo R MH, Russell K, Xiu J, Millis SZ, Reddy SK, Friedlander M. Molecular profile comparison of endometrial, renal and ovarian clear cell carcinoma: Is it the same disease at different sites? J Clin Oncol 2015; 33 suppl: abstr 5595.
23. Gatalica Z, Snyder C, Maney T et al. Programmed cell death 1 (PD-1) and its ligand (PD-L1) in common cancers and their correlation with molecular cancer type. Cancer Epidemiol Biomarkers Prev 2014; 23: 2965-2970.
24. Friedlander ML, Russell K, Millis S et al. Molecular Profiling of Clear Cell Ovarian Cancers: Identifying Potential Treatment Targets for Clinical Trials. Int J Gynecol Cancer 2016; 26: 648-654.
25. Champiat S, Ferte C, Lebel-Binay S et al. Exomics and immunogenics: Bridging mutational load and immune checkpoints efficacy. Oncoimmunology 2014; 3: e27817.
26. Alexandrov LB, Nik-Zainal S, Wedge DC et al. Signatures of mutational processes in human cancer. Nature 2013; 500: 415-421.
27. Kim JM, Chen DS. Immune escape to PD-L1/PD-1 blockade: seven steps to success (or failure). Ann Oncol 2016.
28. Anglesio MS, George J, Kulbe H et al. IL6-STAT3-HIF signaling and therapeutic response to the angiogenesis inhibitor sunitinib in ovarian clear cell cancer. Clin Cancer Res 2011; 17: 2538-2548.
29. Chandler RL, Damrauer JS, Raab JR et al. Coexistent ARID1A-PIK3CA mutations promote ovarian clear-cell tumorigenesis through pro-tumorigenic inflammatory cytokine signalling. Nat Commun 2015; 6: 6118.
30. Motzer RJ, Escudier B, McDermott DF et al. Nivolumab versus Everolimus in Advanced Renal-Cell Carcinoma. N Engl J Med 2015; 373: 1803-1813.
31. Disis ML PM, Pant S, Infante JR, Lockhart AC, Kelly K, Beck JT, Gordon MS, Weiss GJ, Ejadi S, Taylor MH, von Heydebreck A, Chin KM, Cuillerot JM, Gulley JL. Avelumab (MSB0010718C), an anti-PD-L1 antibody, in patients with previously treated, recurrent or refractory ovarian cancer: A phase Ib, open-label expansion trial. J Clin Oncol 2015; 33: abstract number 5509.
32. Hamanishi J, Mandai M, Ikeda T et al. Safety and Antitumor Activity of Anti-PD-1 Antibody, Nivolumab, in Patients With Platinum-Resistant Ovarian Cancer. J Clin Oncol 2015; 33: 4015-4022.
33. Stover E, Matulonis U, Konstantinopoulos P et al. Targeted Next-Generation Sequencing Reveals Clinically Actionable BRAF and ESR1 Mutations in Low-Grade

DocuSign Envelope ID: F89E9DEB-F629-4294-B1D8-B570A4FACE07

Serous Ovarian Carcinoma. JCO Precis Oncol. 2018;2018. doi: 10.1200/PO.18.00135. Epub 2018 Nov 8.

DocuSign Envelope ID: F89E9DEB-F629-4294-B1D8-B570A4FACE07

APPENDIX 1: SCHEDULE OF PROCEDURES/ASSESSMENTS

**Trial Period:**	**Screening Phase (initial & re-treatment)**		**Treatment Cycles - every 3 weeks (initial & re-treatment)**									**End of Treatment (initial & re-treatment)**	**Post-Treatment (initial & re-treatment)**	
Assessment:	Pre-trial screening	Pre-treatment (baseline)	On treatment cycle: (*repeat until treatment discontinuation / progression) 1	2	3	4	5	6	7	8	9	Discontinuation	Follow Up Visits	Survival Follow-Up[b]
Scheduling Window (Days):		Within 28 days prior to registration unless otherwise indicated	Assessments / procedures to be performed within 3 days prior to planned cycle, unless otherwise indicated									30 days post discont ± 7 days	Every 12 weeks post discon ± 7 days	Every 12 weeks post discon ± 7 days
**Administrative Procedures**														
Informed Consent	x													
Inclusion & Exclusion Criteria		x												
Demographics & Medical History Review		x[a]												
Concomitant Medication Review		x	x	x	x	x	x	x	x	x	x	x		
Anticancer therapy post study treatment												x	x	x
Survival Status												x	x	x
BRCA & MMR status (if known)		x												
**Clinical Procedures/Assessments**														
Review Adverse Events[a]			x	x	x	x	x	x	x	x	x	x	x[h]	x[h]
Full Physical Examination		x	x	x	x	x	x	x	x	x	x	x		
Vital Signs and Weight		x	x	x	x	x	x	x	x	x	x	x		
ECOG Performance Status		x	x	x	x	x	x	x	x	x	x	x		
**Laboratory Procedures/Assessments: analysis performed by LOCAL laboratory**														
Pregnancy Test – Urine or Serum βHCG		x[b]	x	x	x	x	x	x	x	x	x	x		
INR		x[c]												
FBC with Differential		x[c]		x	x	x	x	x	x	x	x	x		

Trial Period:	Screening Phase (initial & re-treatment)		Treatment Cycles - every 3 weeks (initial & re-treatment)									End of Treatment (initial & re-treatment)	Post-Treatment (initial & re-treatment)	
			On treatment cycle: (*repeat until treatment discontinuation / progression)											
Assessment:	Pre-trial screening	Pre-treatment (baseline)	1	2	3	4	5	6	7	8	9	Discontinuation	Follow Up Visits	Survival Follow-Up[b]
Scheduling Window (Days):		Within 28 days prior to registration unless otherwise indicated	Assessments / procedures to be performed within 3 days prior to planned cycle, unless otherwise indicated									30 days post discont ± 7 days	Every 12 weeks post discon ± 7 days	Every 12 weeks post discon ± 7 days
Comprehensive Serum Chemistry Panel		x[c]		x	x	x	x	x	x	x	x	x		
Urinalysis		x	x	x	x	x	x	x	x	x	x	x		
Total T3/ Free T4 and TSH		x[c]		x	x	x	x	x	x	x	x	x		
CA 125		x[d]		x	x	x	x	x	x	x	x	x	x[j]	
HIV type 1/2 and Hepatitis B & C testing		x												
**Efficacy Measurements**														
Tumour Imaging		x			x		x				X[l]	x[j]	x[j]	
QoL (FACT-O)		x			x		x				X[l]	x	x[j]	
**Tumour Biopsies/Archival Tissue Collection/Correlative Studies Blood**														
Archival Tissue Collection		x[i]												
Re-biopsy Fresh Tissue Collection		x		x[e]								x[g]		
Exploratory Research Blood Collection		x[k]		x		x		x[f]				x[g]		

Trial Period:	Screening Phase (initial & re-treatment)		Treatment Cycles - every 3 weeks (initial & re-treatment)									End of Treatment (initial & re-treatment)	Post-Treatment (initial & re-treatment)	
Assessment:	Pre-trial screening	Pre-treatment (baseline)	On treatment cycle: (*repeat until treatment discontinuation / progression)									Discontinuation	Follow Up Visits	Survival Follow-Up[b]
			1	2	3	4	5	6	7	8	9			
Scheduling Window (Days):		Within 28 days prior to registration unless otherwise indicated	Assessments / procedures to be performed within 3 days prior to planned cycle, unless otherwise indicated									30 days post discont ± 7 days	Every 12 weeks post discon ± 7 days	Every 12 weeks post discon ± 7 days

a = Pre-existing conditions prior to start of treatment are added as medical history data, any conditions that worsen after treatment then require reporting as an AE.

b = Pregnancy testing required within 3 days prior to registration.

c = Pre-cycle 1, day 1 treatment bloods to be performed within 10 days prior to registration i.e. they do not need to be repeated following baseline for day 1 cycle 1.

d = Ca125 to be performed within 14 days prior to registration.

e = Re-biopsy 6-8 weeks during treatment and upon progression.

f = exploratory bloods at cycles 2, 4 & 6, then at 12 months only.

g = At the time of disease progression.

h = AEs 30 days post last treatment, SAEs up until 110 days post last treatment and longer if a late term SAR, and AESIs up until 110 days post treatment.

i = Archival tissue collected.

j = If patient has not progressed, imaging, CA125 and QoL continues until progression.

K = Baseline exploratory bloods can also be taken after registration and pre cycle 1 day 1, if logistically more suitable to take at that time.

L= Imaging & QoL every 12 weeks until end of treatment (also see code J).

DocuSign Envelope ID: F89E9DEB-F629-4294-B1D8-B570A4FACE07

APPENDIX 2: ABBREVIATIONS

**AE**	Adverse Event
**AESI**	Adverse Event of Special Interest
**ALP**	Alkaline Phosphatase
**ALT**	Alanine Transaminase
**ANC**	Absolute Neutrophil Count
**aPTT**	Activated partial thromboplastin time
**AR**	Adverse Reaction
**ARID1A**	AT-Rich Interaction Domain 1A
**AST**	Aspartate Aminotransferase
**AUC**	Area Under the Curve
**BP**	Blood Pressure
**BRCA**	BReast CAncer Susceptibility Genes 1 and 2
**CCEC**	Clear Cell Endometrial Cancer
**CCOC**	Clear Cell Ovarian Cancer
**CI**	Chief Investigator
**CR**	Complete Response
**CT**	Computerised Tomography
**CTA**	Clinical Trial Authorisation
**CTCAE**	Common Terminology Criteria for Adverse Events
**CXR**	Chest X-Ray
**DOR**	Duration of Response
**DSUR**	Development Safety Update Report
**eCRF**	Electronic Case Report Form
**EGFR**	Epidermal Growth Factor Receptor
**ECG**	Electrocardiogram
**ECOG**	Eastern Cooperative Oncology Group
**EudraCT**	European Clinical Trials Database
**FBC**	Full Blood Count
**GFR**	Glomerular Filtration Rate
**GCP**	Good Clinical Practice
**Hb**	Haemoglobin
**hCG**	Human Chorionic Gonadotropin
**HIV**	Human Immunodeficiency Virus
**HR**	Hazard Ratio
**HRA**	Health Research Authority
**HRD**	Homologous Recombination Deficiency
**IB**	Investigator's Brochure
**ICH GCP**	International Conference of Harmonisation-Good Clinical Practice
**IDMC**	Independent Data Monitoring Committee
**IMP**	Investigational Medicinal Product
**INR**	International Normalised Ratio
**IV**	Intravenous

**LDH**	Lactate Dehydrogenase
**LFT**	Liver Function Tests
**LLN**	Lower Limit of Normal
**mAB**	Monoclonal Antibody
**MDM 2/4**	Murine double minute 2/4 (oncogenes)
**MHRA**	Medicines and Healthcare products Regulatory Agency
**MMR**	Mismatch Repair
**MRI**	Magnetic Resonance Image
**MSD**	Merck Sharp & Dohme Limited
**NCRI**	National Cancer Research Institute
**NIHR**	National Institute for Health Research
**ORR**	Objective Response Rate
**OS**	Overall Survival
**OTC**	Over the Counter
**PD**	Progressive Disease
**PBMC**	Peripheral blood mononuclear cells
**PD1**	Programmed cell death 1
**PDL1**	Programmed Death-ligand 1
**PFS**	Progression Free Survival
**PI**	Principal Investigator
**PR**	Partial Response
**PT**	Prothrombin Time
**Q3W**	Every 3 weeks
**REC**	Research Ethics Committee
**RECIST**	Response Evaluation Criteria in Solid Tumours
**SAE**	Serious Adverse Event
**SAR**	Serious Adverse Reaction
**SD**	Stable Disease
**SGOT**	Serum Glutamic-oxaloacetic Transaminase
**SGPT**	Serum Glutamic-pyruvic Transaminase
**SUSAR**	Suspected Unexpected Serious Adverse Reaction
**T1DM**	Type 1 Diabetes Mellitus
**T3**	Triiodothyronine
**T4**	Thyroxine
**TB**	Tuberculosis
**TILs**	Tumour Infiltrating Lymphocytes
**TSH**	Thyroid Stimulating Hormone
**TMF**	Trial Master File
**TMG**	Trial Management Group
**TSC**	Trial Steering Committee
**UCL CTC**	CR UK and UCL Cancer Trials Centre
**U&E**	Urea and Electrolytes
**ULN**	Upper Limit of Normal
**WBC**	White Blood Cells

APPENDIX 3: PROTOCOL VERSION HISTORY

Protocol Version no.	Date	Amendment no.	Protocol Section (no./title)	Summary of main changes from previous version.
1	11/07/2019	N/A	N/A	N/A
2	11/11/2019	04	TMG Table	Updated Trial Management Group (TMG).
			1.2 & 7.2	Adjusted eligibility:<br>- Change in serum calcium value.<br>- Clarification on measurable disease and biopsiable lesion.<br>- Addition of archival tissue collection.
			8.1.1, 10, 10.3 & Appendix 1	Clarifications on tumour assessment timings throughout.
			13.2.2	Updated wording for the collection of SAEs for patients in the screening period: Screening SAEs are collected until 14 days post last screening assessment
			11.1.1	Addition of the collection of archival tissue.
			11.1.2	Clarification added for the biopsy collection process; Any measurable lesion should be separate from the biopsied lesion.
			12	Updated wording to reflect the inclusion of electronic case report forms.
			9.5	Management of treatment related adverse events updated, following TMG review and to reflect current IB v16.<br>- Based on postmarketing environments immune-mediated ADRs may be fatal and may occur after discontinuation of pembrolizumab.<br>- Arthritis ARs have been identified during post-approval use of pembrolizumab.
			9.8.2	Prohibited therapies updated with the inclusion of radiotherapy.
			N/A	Minor wording clarifications applied throughout.
3.0	09/12/2020	11	TMG Table	Updated Trial Management Group (TMG) and contacts.
			8.1.1, 10.2, 10.5 & Appendix 1	Updated timings for the collection of exploratory blood and tissue samples.
			Section 6	Re-consenting instructions for sites added.
			Table 4	Table 4: Management Guidelines for Drug-Related Adverse Events has been updated for IMP management (discontinuation of IMP) of grade 3 or 4 AEs.

**Protocol Version no.**	**Date**	**Amendment no.**	**Protocol Section (no./title)**	**Summary of main changes from previous version.**
			Section 15.2	Central monitoring updated to include remote monitoring.
4	25/08/2021	13	Protocol authorisation	UCL CTC Director removed – the TGL only will authorise on behalf of the Sponosr
			Trial contacts	Fax number removed.
			9.5 Management of AEs	Table 4 updated in line with IMP manufacturer's guidance.
			Section 9.8.1 Acceptable Concomitant Medications	Guidance added as requested in line with MHRA advice for Covid-19 vaccinations and IMP administration.
			Section 7.3.5 Action to be taken in the event of a pregnancy	Wording adjusted in line with updated UCL CTC SOPs.
			9.10 24 Hour/Out-of-Office Hours Emergency Drug-Specific Advice	MSD contact number updated.
			Section 13 Pharmacovigilance	Minor wording clarifications added in line with update UCL CTC SOPs.
			Section 15 Trial monitoring and oversight	Wording adjusted in line with updated UCL CTC SOPs.
5	23/08/2022	15	TMG	New members added (Exploratory analysis researchers)
			Section 3.1.3.1.3 'Exploratory Endpoint' and section 4.2.3 'Exploratory Outcomes'	RNA Sequencing included
			Section 8 'Registration Procedures'	Instructions for GP letter dissemination added
			Section 9.5 'Management of AES'	Management of further Immune-mediated irARs added following receipt of an updated Pembro IB (version21)
			Section 13 'Pharmacovigilance'	Regulation reference updated
			Section 14.1 'Incident reporting'	Additional guidance added for sites when sending reports to UCL CTC
			Section 15.5.4 'Role of UCL CTC'	Data custodian updated

Protocol Version no.	Date	Amendment no.	Protocol Section (no./title)	Summary of main changes from previous version.
			Section 17.1 'End of trial'	Timeframe of follow up for last patient updated and inclusion of completion of all exploratory endpoint research.
			Section 19 'Ethical and regulatory considerations'	Regulation reference updated
			20.1 'Sponsor Details'	Address updated
			Appendix 1	Clarifications/edits applied
