# Supplementary material for: Pembrolizumab in Patients With Advanced Clear Cell Gynecological Cancer: A Phase 2 Nonrandomized Clinical Trial
Source: JAMA Oncol. 2025 Feb 6;11(4):377–85. doi: 10.1001/jamaoncol.2024.6797 (PMC11803509; doi:10.1001/jamaoncol.2024.6797)
Supplement: Supplement 2. — eTable 1. Antibodies Used for Immunohistochemistry Analysis eTable 2. Recruitment eTable 3. Treatment-Related Adverse Events (Grade 1-5 in Any Patient) eTable 4. Adverse Events of Any Relatedness (Grade 3-5 in Any Patient) eTable 5. Quality of Life (QoL) by Visit (A) and Change From Baseline (B) eFigure 1. Quality of Life by Visit (A), Change From Baseline (B) and Change From Baseline by Disease Status at 12 Weeks (C) eFigure 2. Progression-Free Survival by ARID1A (A), MMR (B), p53 (C), PD1 (D), PDL1 (E) and PD1/PDL1 Combined (F) eFigure 3. Overall Survival by ARID1A (A), MMR (B), p53 (C), PD1 (D), PDL1 (E) and PD1/PDL1 Combined (F) [file jamaoncol-e246797-s002.pdf]

Supplemental Online Content

Kristeleit R, Devlin MJ, Clamp A, et al. Pembrolizumab in patients with advanced clear cell gynecological cancer: a phase 2 nonrandomized clinical trial. *JAMA Oncol*. Published online February 6, 2025. doi:10.1001/jamaoncol.2024.6797

eTable 1. Antibodies Used for Immunohistochemistry Analysis

eTable 2. Recruitment

eTable 3. Treatment-Related Adverse Events (Grade 1-5 in Any Patient)

eTable 4. Adverse Events of Any Relatedness (Grade 3-5 in Any Patient)

eTable 5. Quality of Life (QoL) by Visit (A) and Change From Baseline (B)

eFigure 1. Quality of Life by Visit (A), Change From Baseline (B) and Change From Baseline by Disease Status at 12 Weeks (C)

eFigure 2. Progression-Free Survival by ARID1A (A), MMR (B), p53 (C), PD1 (D), PDL1 (E) and PD1/PDL1 Combined (F)

eFigure 3. Overall Survival by ARID1A (A), MMR (B), p53 (C), PD1 (D), PDL1 (E) and PD1/PDL1 Combined (F)

This supplementary material has been provided by the authors to give readers additional information about their work.

© 2025 Kristeleit R et al. *JAMA Oncology*.

eTable 1. Antibodies Used for Immunohistochemistry Analysis

Target	Company	Catalog Number	Concentration
ARID1A	Abcam	ab182561	1:1000
p53	Leica	PA0057	RTU
MHL1	Agilent	ES05	1:50
MSH2	Leica	NCLMSH2	1:50
PMS2	BD Bioscience	556415	1:300
MSH6	Agilent	M364601	1:100
PD1	Cell Marque	315-M	1:50
PDL1	Agilent	SK00621-2	RTU

Abbreviations: ready to use (RTU)

© 2025 Kristeleit R et al. *JAMA Oncology*.

eTable 2. Recruitment

Participating UK Site	Number of Patients
University College London Hospitals, London	24
The Christie NHS Foundation Trust, Manchester	10
Western General Hospital, Edinburgh	8*
Churchill Hospital, Oxford	5
Mount Vernon Hospital, Middlesex	2
**Total**	**49**

* One patient became ineligible prior to receiving trial treatment and therefore were withdrawn from trial analysis.

© 2025 Kristeleit R et al. *JAMA Oncology*.

eTable 3. Treatment-Related Adverse Events (Grade 1-5 in Any Patient)

Adverse Event Term*	Grade 1 (N=48)	Grade 2 (N=48)	Grade 3 (N=48)	Grade 4 (N=48)	Grade 5 (N=48)
Abdominal distension	0	1 (2%)	0	0	0
Abdominal pain	2 (4%)	0	0	0	0
Acute kidney injury	0	0	1 (2%)	0	0
Adrenal insufficiency	0	1 (2%)	0	0	0
Alanine aminotransferase increased	1 (2%)	2 (4%)	2 (4%)†	0	0
Alkaline phosphatase increased	2 (4%)	2 (4%)	1 (2%)	0	0
Anaemia	0	0	1 (2%)	0	0
Anorexia	4 (8%)	3 (6%)	0	0	0
Arthralgia	1 (2%)	0	0	0	0
Aspartate aminotransferase increased	1 (2%)	2 (4%)	1 (2%)†	0	0
Back pain	1 (2%)	0	0	0	0
Bone pain	1 (2%)	0	0	0	0
Chills	1 (2%)	0	0	0	0
Cough	3 (6%)	0	0	0	0
Creatinine increased	0	1 (2%)	0	0	0
Cystitis noninfective	1 (2%)	0	0	0	0
Diarrhea	8 (17%)	1 (2%)	0	0	0
Dry eye	1 (2%)	0	0	0	0
Dry mouth	4 (8%)	1 (2%)	0	0	0
Dry skin	4 (8%)	0	0	0	0
Dysgeusia	3 (6%)	0	0	0	0
Dyspepsia	1 (2%)	0	0	0	0
Dysphagia	1 (2%)	0	0	0	0
Eczema	1 (2%)	0	0	0	0
Edema limbs	1 (2%)	0	0	0	0
Erythema	1 (2%)	0	0	0	0
Eye disorders - other: periorbital erythema	1 (2%)	0	0	0	0
Fatigue	8 (17%)	6 (13%)	0	0	0
Fever	1 (2%)	0	0	0	0
Gastroesophageal reflux disease	1 (2%)	0	0	0	0
General disorders and administration site conditions - other: peripheral oedema (legs)	1 (2%)	0	0	0	0
GGT increased	0	0	1 (2%)†	0	0
Headache	2 (4%)	0	0	0	0
Hypertension	1 (2%)	0	0	0	0
Hyperthyroidism	3 (6%)	2 (4%)	2 (4%)	0	0
Hypoglycemia	0	1 (2%)	0	0	0
Hypothyroidism	3 (6%)	7 (15%)	0	0	0
Immune related hepatitis	0	0	1 (2%)†	0	0
Irritability	1 (2%)	0	0	0	0
Localised edema	1 (2%)	0	0	0	0

© 2025 Kristeleit R et al. *JAMA Oncology*.

Lymph node pain	1 (2%)	0	0	0	0
Metabolism and nutrition disorders - other: ketoacidosis	0	0	1 (2%)	0	0
Mucositis oral	4 (8%)	0	0	0	0
Muscle cramp	1 (2%)	0	0	0	0
Myalgia	1 (2%)	0	0	0	0
Nausea	5 (10%)	2 (4%)	0	0	0
Nervous system disorders - other: noninfective encephalitis	0	0	1 (2%)	0	0
Pain	2 (4%)	0	0	0	0
Pain in extremity	2 (4%)	0	0	0	0
Palmar-plantar erythrodysesthesia syndrome	1 (2%)	0	0	0	0
Pneumonitis	0	1 (2%)	0	0	0
Pruritus	8 (17%)	2 (4%)	0	0	0
Rash maculo-papular	6 (13%)	1 (2%)	0	0	0
Rash pustular	0	1 (2%)	0	0	0
Restlessness	1 (2%)	0	0	0	0
Sinus tachycardia	0	1 (2%)	0	0	0
Skin and subcutaneous tissue disorders - other: dermatitis	0	1 (2%)	0	0	0
Skin and subcutaneous tissue disorders - other: rash	2 (4%)	1 (2%)	0	0	0
Skin and subcutaneous tissue disorders - other: skin changes	2 (4%)	0	0	0	0
Stomach pain	1 (2%)	0	0	0	0
Vomiting	3 (6%)	0	0	0	0
Weight loss	0	1 (2%)	0	0	0
Any adverse event*	***10 (21%)***	***14 (29%)***	***9† (19%)***	***0 (0%)***	***0 (0%)***

*Maximum grade of each term per patient (i.e. each patient may be counted for more than one adverse event term)
**Maximum grade per patient (i.e. each patient counted only once)
†Eight patients had 1x grade 3 adverse event; one patient had 4x different grade 3 adverse events (alanine aminotransferase increased, aspartate aminotransferase increased, GGT increased and immune related hepatitis).

© 2025 Kristeleit R et al. *JAMA Oncology*.

eTable 4. Adverse Events of Any Relatedness (Grade 3-5 in Any Patient)

Adverse Event Term*	Grade 3 (N=48)	Grade 4 (N=48)	Grade 5 (N=48)
Abdominal pain	3 (6%)	0	0
Acute kidney injury	2 (4%)	0	0
Alanine aminotransferase increased	2 (4%)	0	0
Alkaline phosphatase increased	3 (6%)	0	0
Anaemia	2 (4%)	0	0
Arterial thromboembolism	0	0	1 (2%)
Ascites	2 (4%)	0	0
Aspartate aminotransferase increased	1 (2%)	0	0
Back pain	1 (2%)	0	0
Bronchopulmonary haemorrhage	1 (2%)	0	0
Colonic obstruction	1 (2%)	0	0
Confusion	1 (2%)	0	0
Constipation	1 (2%)	0	0
Dehydration	1 (2%)	0	0
Delirium	1 (2%)	0	0
Diarrhea	2 (4%)	0	0
Duodenal obstruction	1 (2%)	0	0
Duodenal ulcer	1 (2%)	0	0
Dyspnea	2 (4%)	0	0
Fatigue	3 (6%)	0	0
Fever	1 (2%)	0	0
Gastric haemorrhage	1 (2%)	0	0
GGT increased	1 (2%)	0	0
Headache	1 (2%)	0	0
Hypercalcemia	1 (2%)	2 (4%)	0
Hypertension	1 (2%)	0	0
Hyperthyroidism	2 (4%)	0	0
Immune related hepatitis	1 (2%)	0	0
Infection	1 (2%)	0	0
Infections and infestations - other: abscess	1 (2%)	0	0
Intracranial heamorrhage	1 (2%)	0	0
Lung infection	2 (4%)	0	0
Metabolism and nutrition disorders - other: ketoacidosis	1 (2%)	0	0
Nausea	1 (2%)	0	0
Nervous system disorders - other: noninfective encephalitis	1 (2%)	0	0
Pain	1 (2%)	0	0
Pelvic pain	1 (2%)	0	0
Pneumothorax	1 (2%)	0	0
Thromboembolic event	3 (6%)	0	1 (2%)
Urinary tract infection	2 (4%)	0	0
Vomiting	3 (6%)	0	0
Weight gain	1 (2%)	0	0
Any adverse event*	***25 (52%)***	***2 (4%)***	***2 (4%)***

*Maximum grade of each term per patient (i.e. each patient may be counted for more than one adverse event term).
**Maximum grade per patient (i.e. each patient counted only once).

© 2025 Kristeleit R et al. *JAMA Oncology*.

eTable 5. Quality of Life (QoL) by Visit (A) and Change From Baseline (B)

(A) Visit	*n*	Physical Well-Being (0-28) *mean (std.dev)*	Social Well-Being (0-28) *mean (std.dev)*	Emotional Well-Being (0-24) *mean (std.dev)*	Functional Well-Being (0-28) *mean (std.dev)*
Baseline	46	22.3 (4.46)	23.4 (4.49)	15.2 (5.41)	18.6 (6.08)
Week 6	33	22.0 (5.88)	23.6 (4.20)	16.5 (5.27)	18.2 (5.92)
Week 12	28	23.3 (4.61)	23.5 (4.90)	17.3 (5.46)	19.0 (6.66)
Week 24	16	25.9 (2.52)	24.9 (2.46)	17.8 (4.20)	21.3 (5.57)
Week 36	12	25.8 (3.07)	24.1 (3.78)	17.9 (4.53)	22.0 (5.62)
End of treatment	29	21.8 (5.72)	22.3 (4.54)	15.6 (4.72)	17.8 (6.36)
		**Ovarian Cancer Subscale (0-44)** *mean (std.dev)*	**Trial Outcome Index (0-100)** *mean (std.dev)*	**FACT-G total score (0-108)** *mean (std.dev)*	**FACT-O total score (0-152)** *mean (std.dev)*
Baseline	46	32.4 (6.38)	73.4 (14.24)	79.6 (14.90)	112.0 (19.33)
Week 6	33	31.9 (6.64)	72.1 (16.26)	80.3 (16.41)	112.2 (21.80)
Week 12	28	33.4 (7.20)	75.7 (16.97)	83.6 (17.99)	117.1 (24.26)
Week 24	16	35.0 (4.80)	82.2 (10.95)	89.9 (10.38)	124.9 (13.34)
Week 36	12	36.0 (4.76)	83.8 (11.31)	89.8 (13.66)	125.8 (16.15)
End of treatment	29	30.9 (7.73)	70.4 (17.31)	77.5 (16.37)	108.3 (23.14)

The higher the score, the better the QoL. N<10 for later timepoints (data not shown).

(B) Change from baseline	*n*	Physical Well-Being (0-28) *mean (std.dev)*	Social Well-Being (0-28) *mean (std.dev)*	Emotional Well-Being (0-24) *mean (std.dev)*	Functional Well-Being (0-28) *mean (std.dev)*
Week 6	33	-0.6 (5.03)	0.4 (3.92)	1.6 (2.96)	-0.0 (3.35)
Week 12	28	0.4 (4.48)	-0.5 (2.77)	1.6 (3.24)	0.4 (4.87)
Week 24	16	2.0 (3.87)	1.5 (6.80)	1.6 (4.94)	0.0 (5.48)
Week 36	12	2.0 (4.14)	-0.1 (4.62)	2.8 (4.53)	0.7 (6.29)
End of treatment	29	-1.3 (5.40)	-1.3 (3.43)	-0.3 (4.29)	-1.8 (6.72)
		**Ovarian Cancer Subscale (0-44)** *mean (std.dev)*	**Trial Outcome Index (0-100)** *mean (std.dev)*	**FACT-G total score (0-108)** *mean (std.dev)*	**FACT-O total score (0-152)** *mean (std.dev)*
Week 6	33	-0.3 (5.03)	-0.9 (11.36)	1.4 (9.78)	1.2 (13.65)
Week 12	28	0.3 (6.09)	1.1 (13.74)	1.9 (11.74)	1.9 (16.54)
Week 24	16	2.2 (5.73)	4.2 (13.44)	5.1 (9.87)	7.2 (14.00)
Week 36	12	3.5 (3.92)	6.1 (11.99)	5.3 (7.80)	8.7 (9.66)
End of treatment	29	-2.0 (7.87)	-5.1 (17.12)	-4.7 (14.84)	-6.7 (20.88)

Positive/negative change from baseline indicates better/worse QOL respectively. N<10 for later timepoints (data not shown).

© 2025 Kristeleit R et al. *JAMA Oncology*.

eFigure 1. Quality of Life by Visit (A), Change From Baseline (B) and Change From Baseline by Disease Status at 12 Weeks (C)

(A)

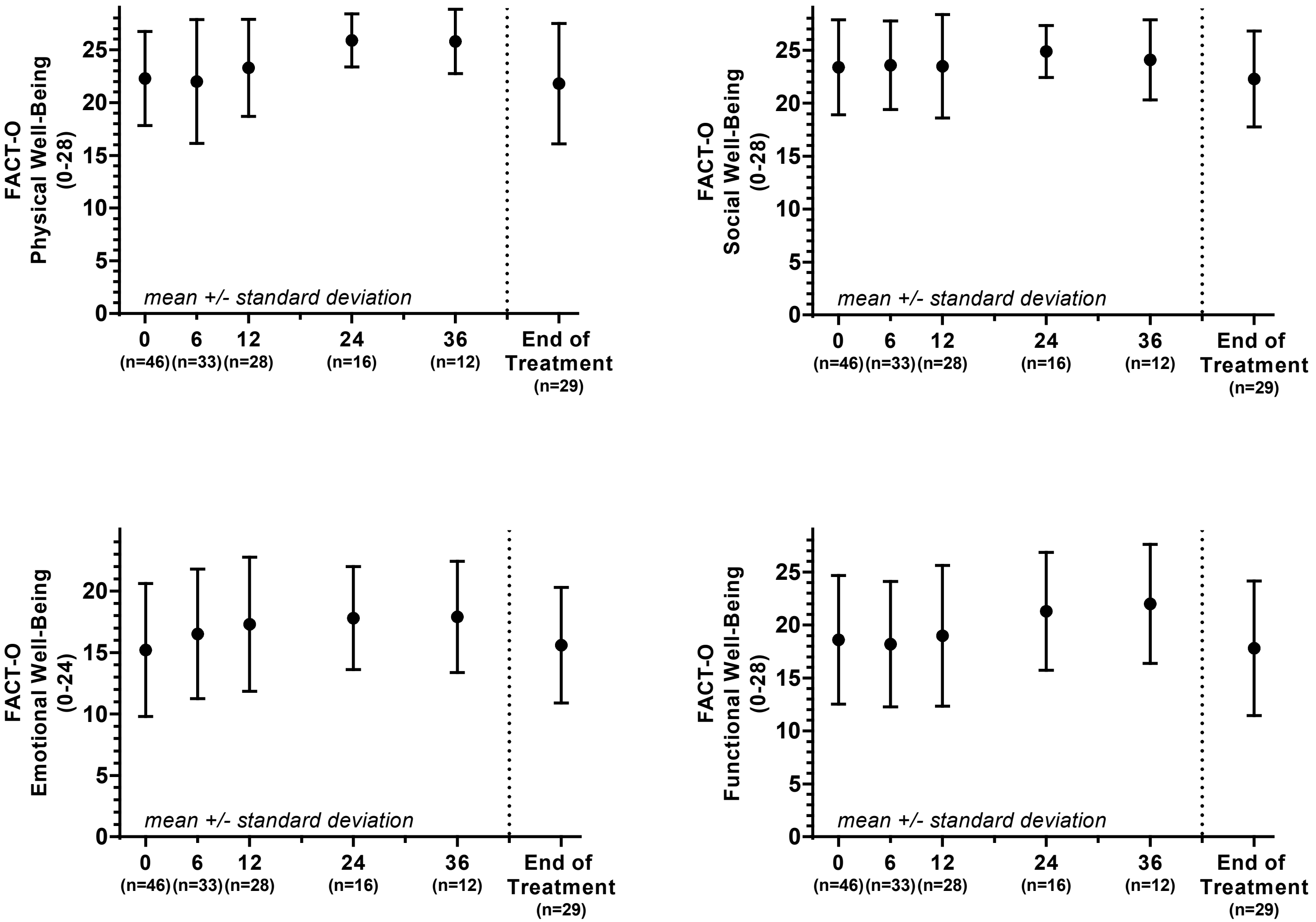

© 2025 Kristeleit R et al. *JAMA Oncology*.

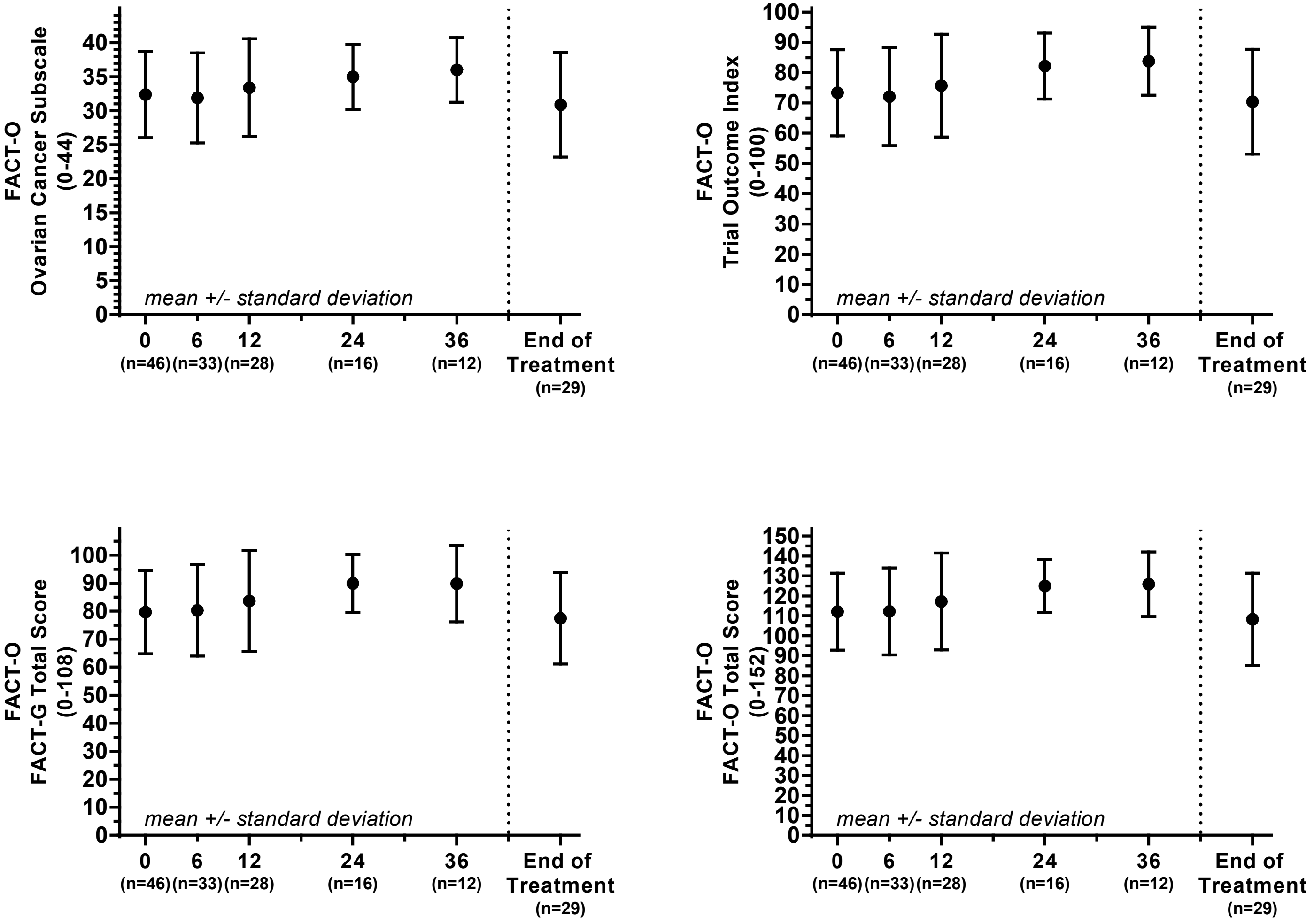

© 2025 Kristeleit R et al. *JAMA Oncology*.

(B)

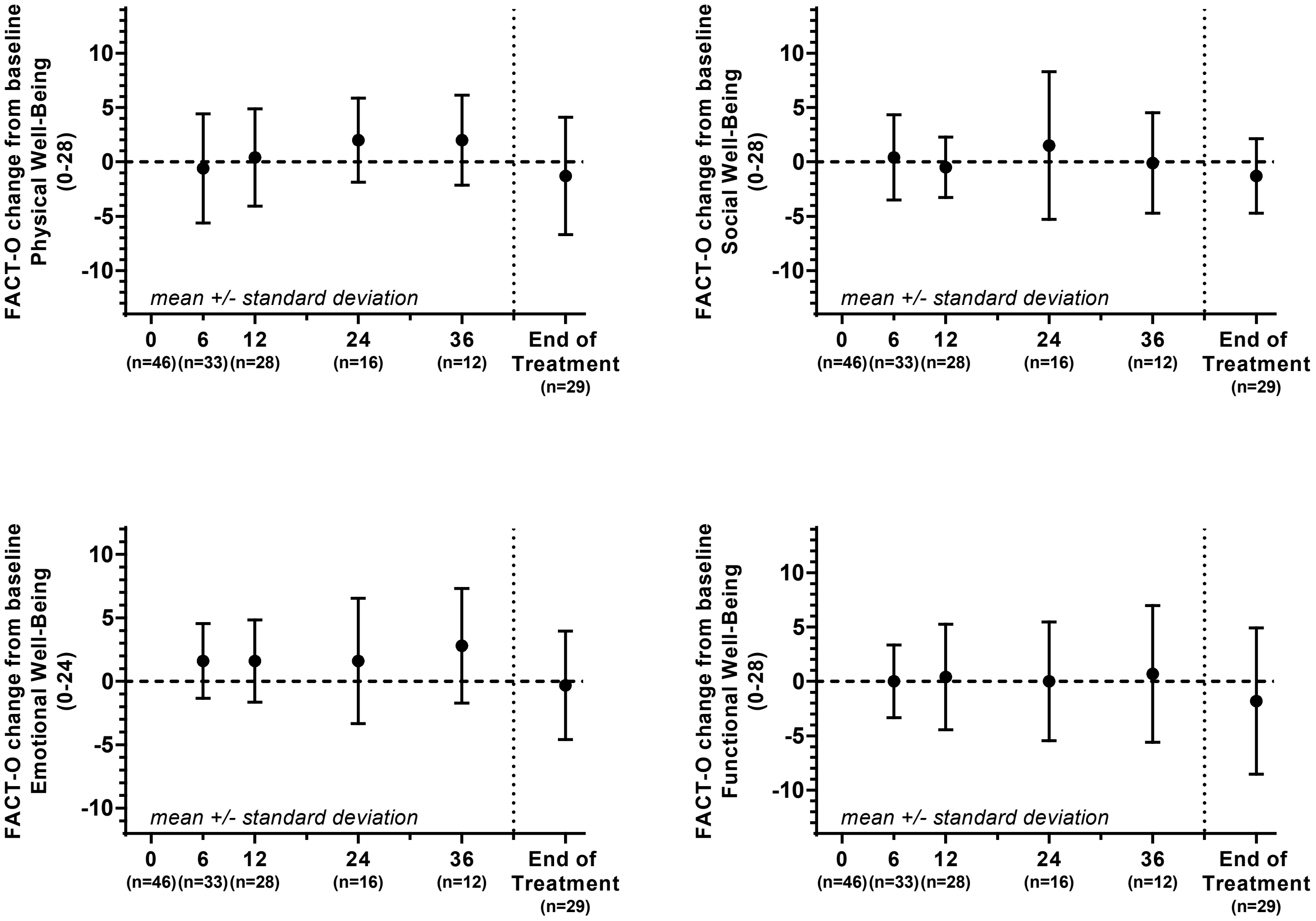

© 2025 Kristeleit R et al. *JAMA Oncology*.

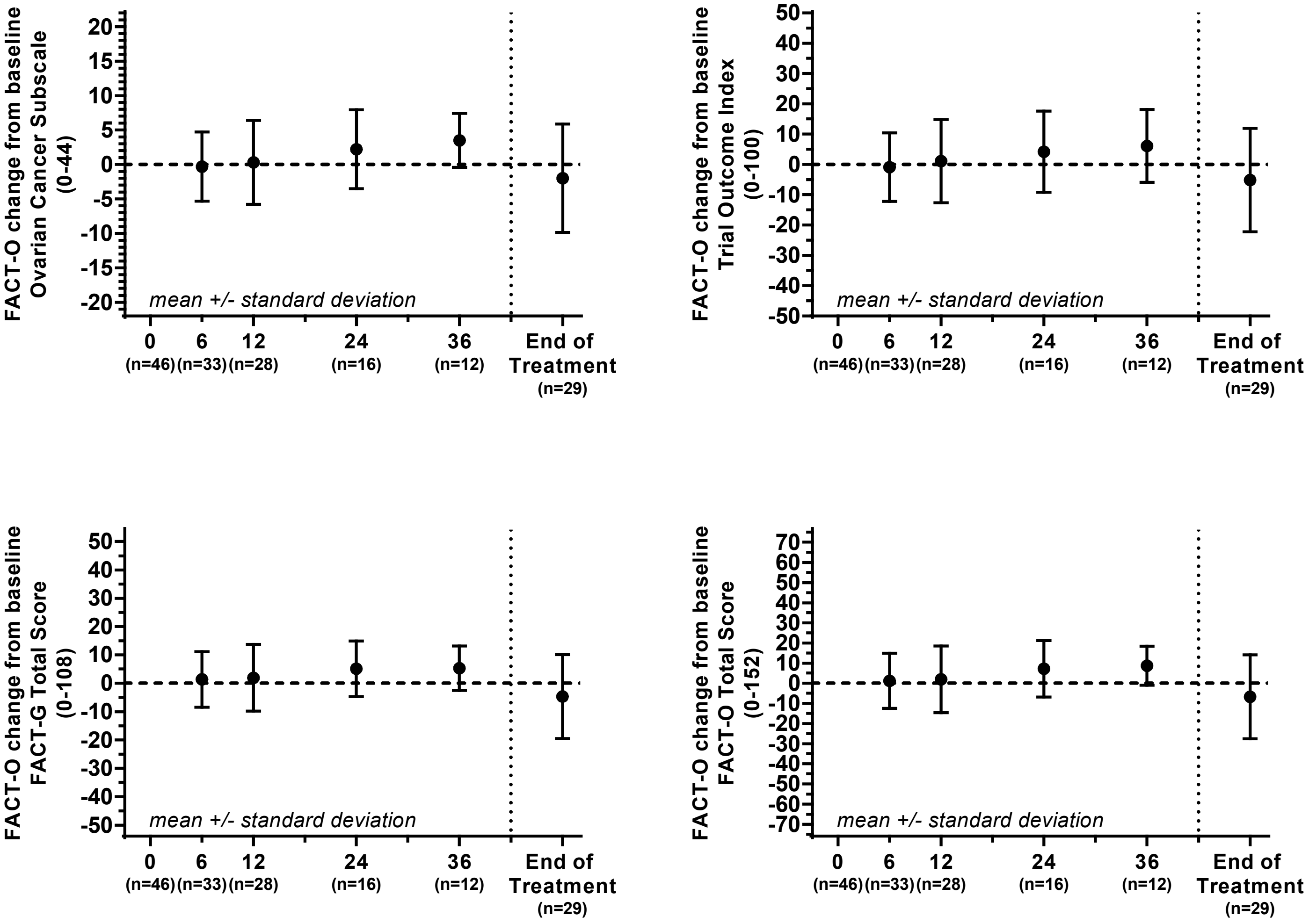

© 2025 Kristeleit R et al. *JAMA Oncology*.

(C)

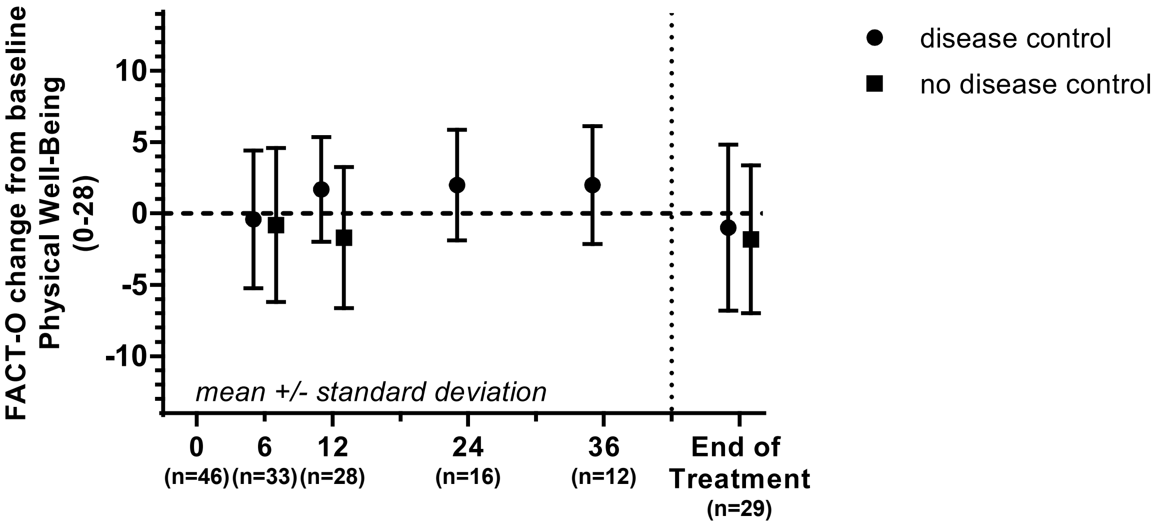

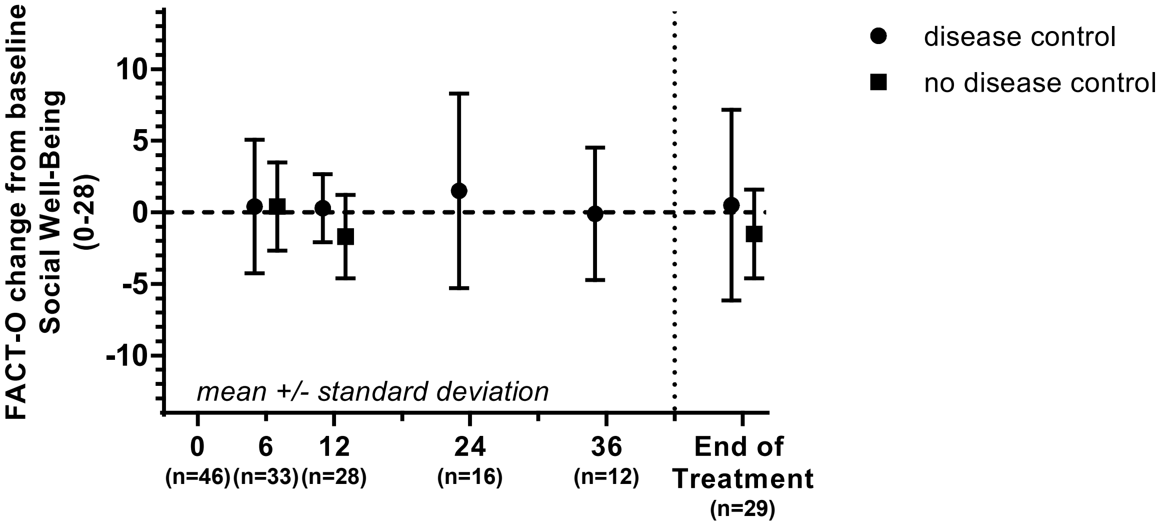

© 2025 Kristeleit R et al. *JAMA Oncology*.

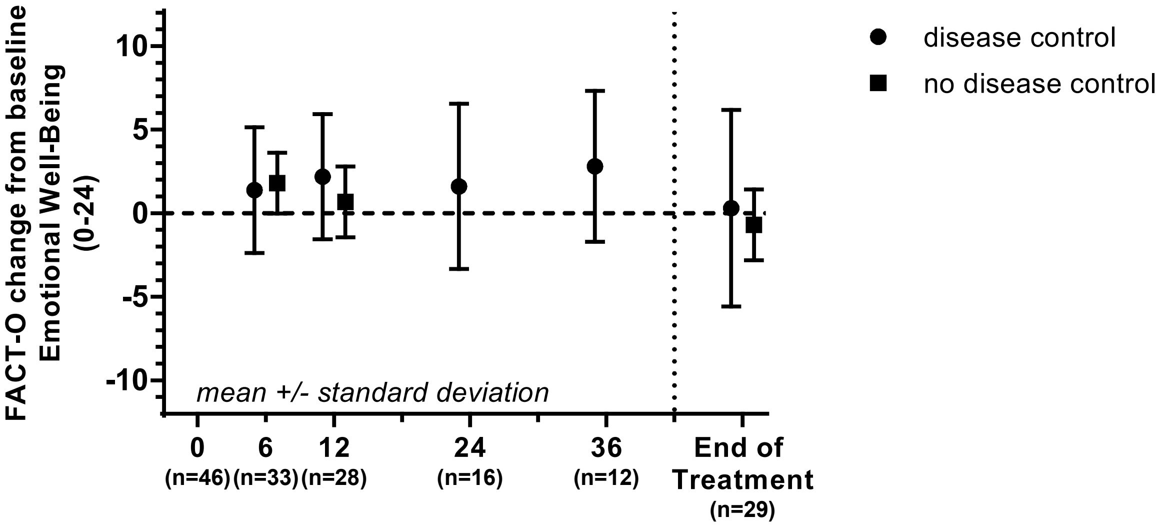

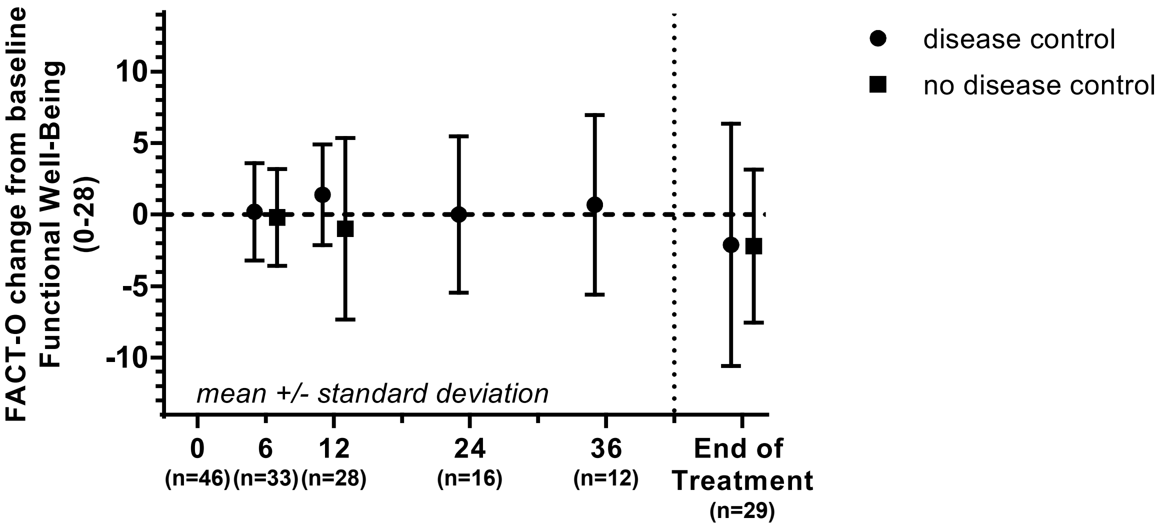

© 2025 Kristeleit R et al. *JAMA Oncology*.

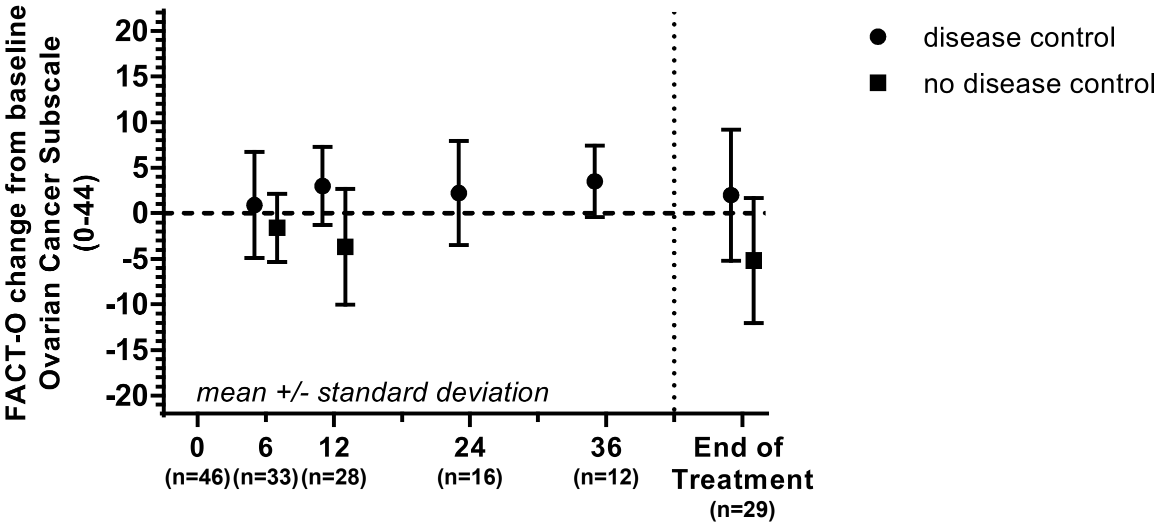

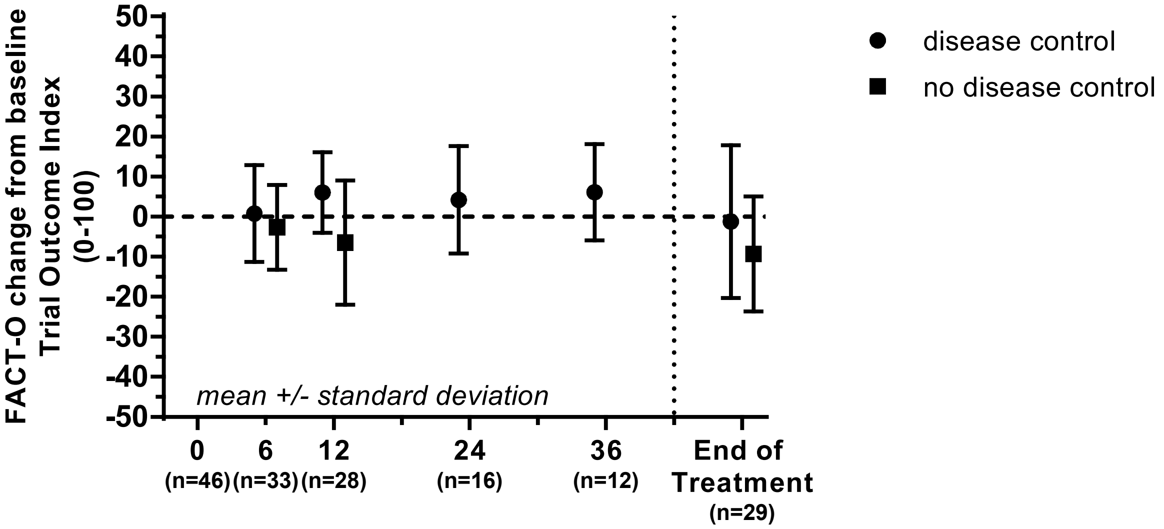

© 2025 Kristeleit R et al. *JAMA Oncology*.

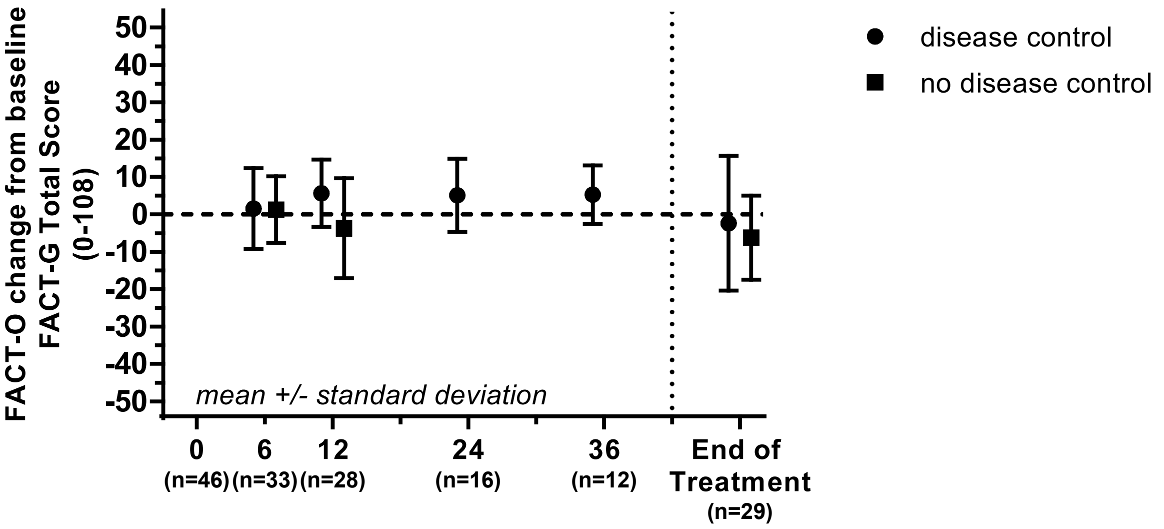

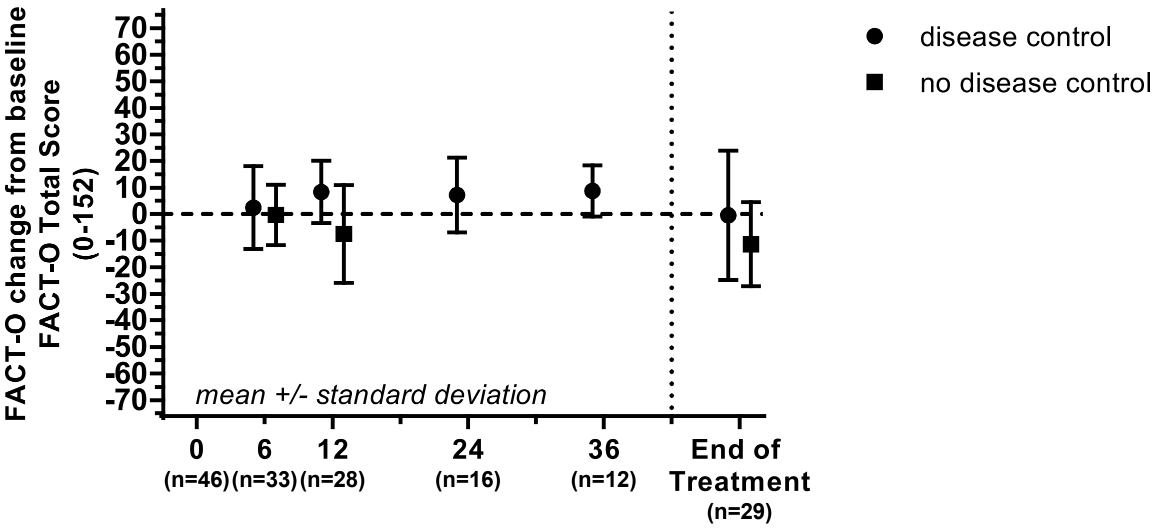

© 2025 Kristeleit R et al. *JAMA Oncology*.

eFigure 2. Progression-Free Survival by ARID1A (A), MMR (B), p53 (C), PD1 (D), PDL1 (E) and PD1/PDL1 Combined (F)

(A)

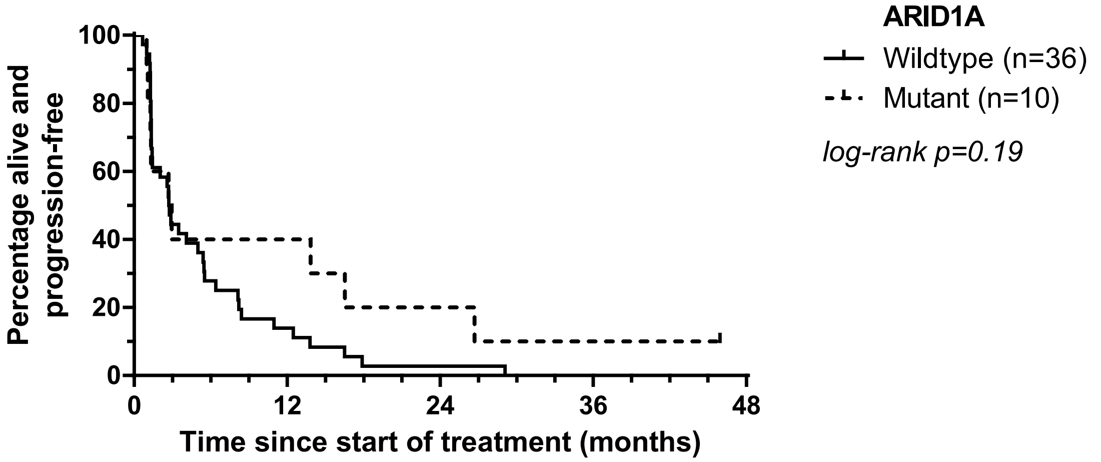

(B)

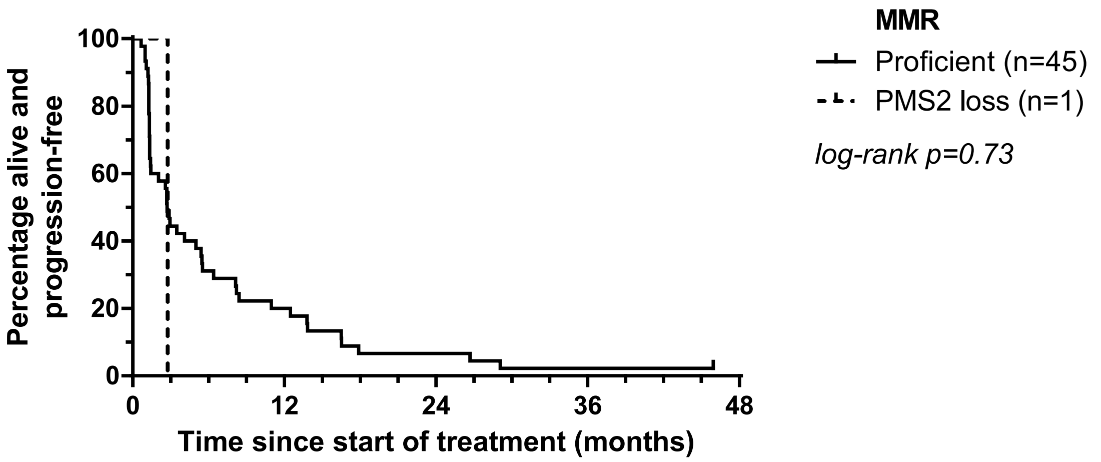

(C)

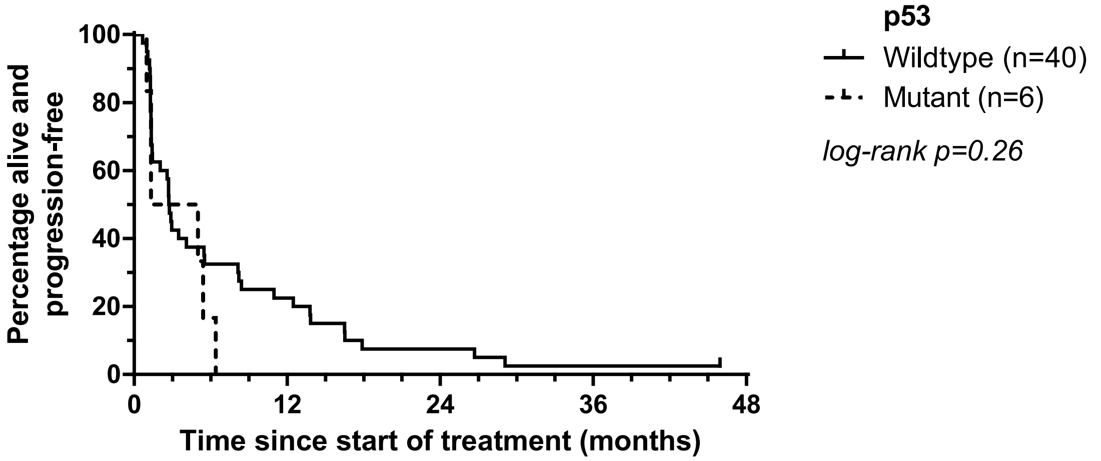

© 2025 Kristeleit R et al. *JAMA Oncology*.

(D)

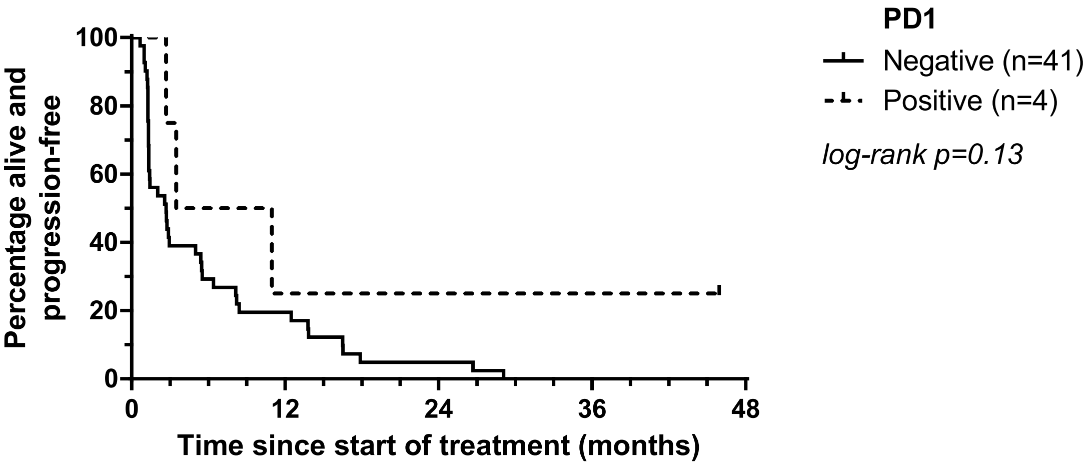

(E)

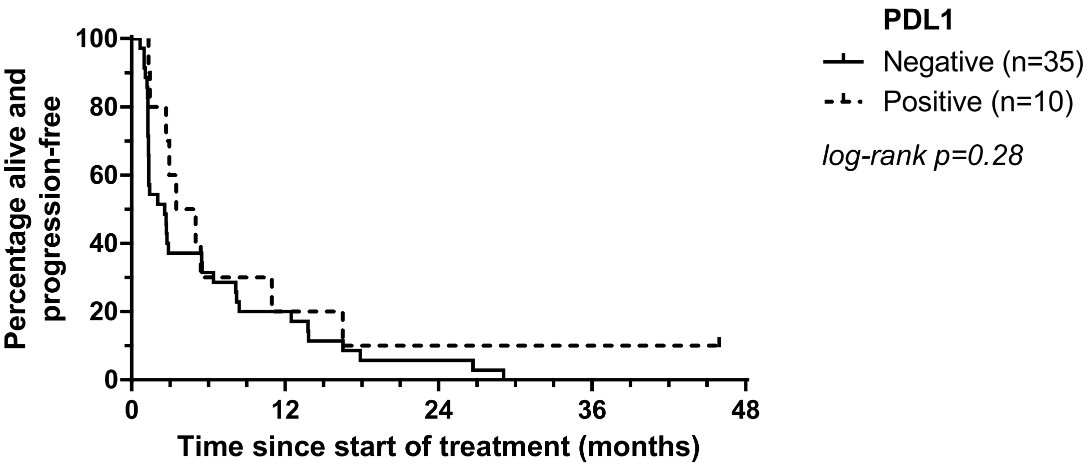

(F)

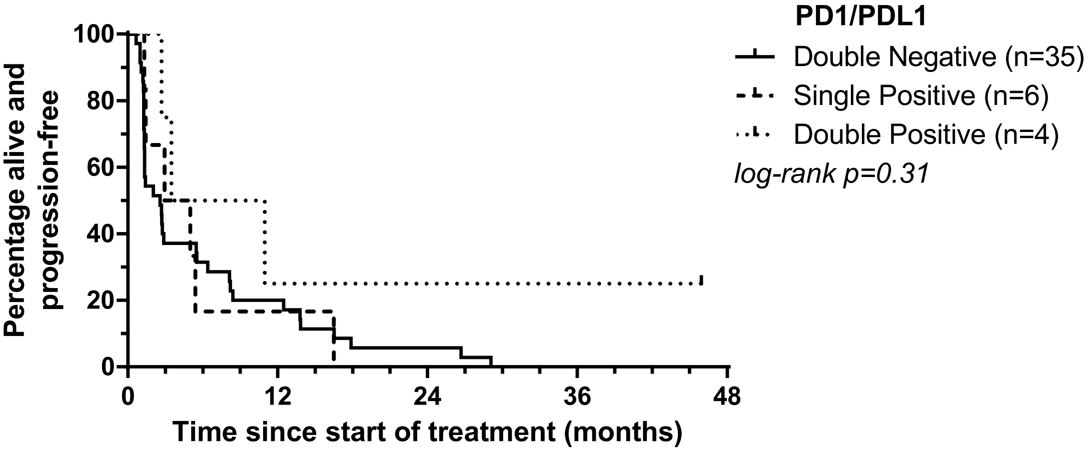

© 2025 Kristeleit R et al. *JAMA Oncology*.

eFigure 3. Overall Survival by ARID1A (A), MMR (B), p53 (C), PD1 (D), PDL1 (E) and PD1/PDL1 Combined (F)

(A)

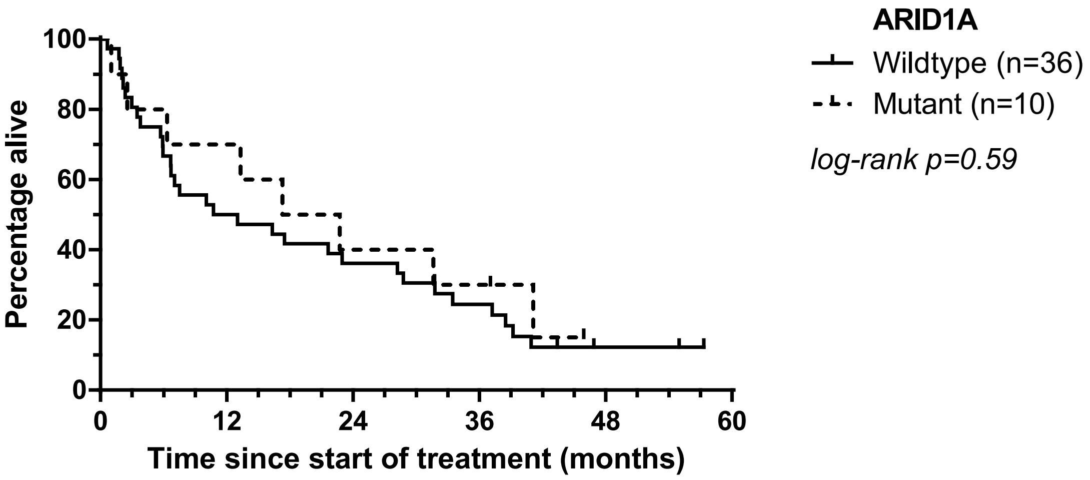

(B)

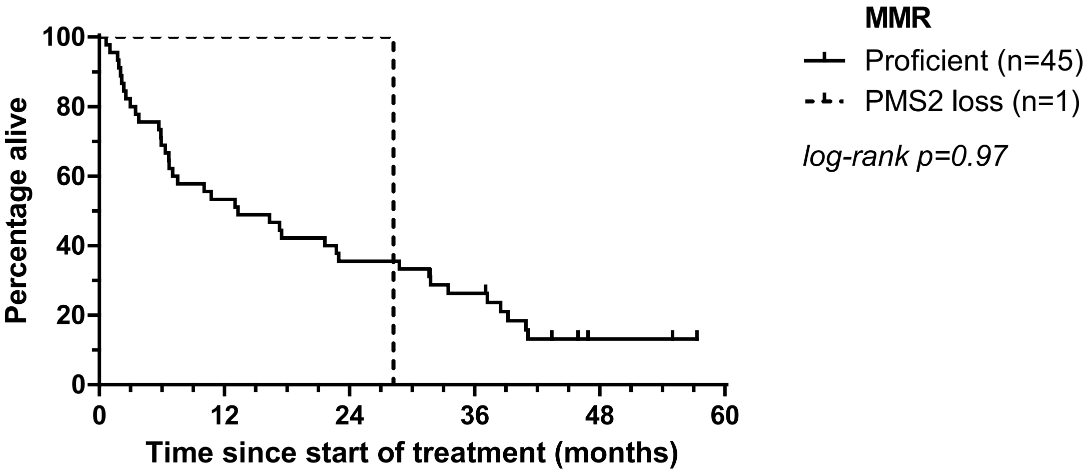

(C)

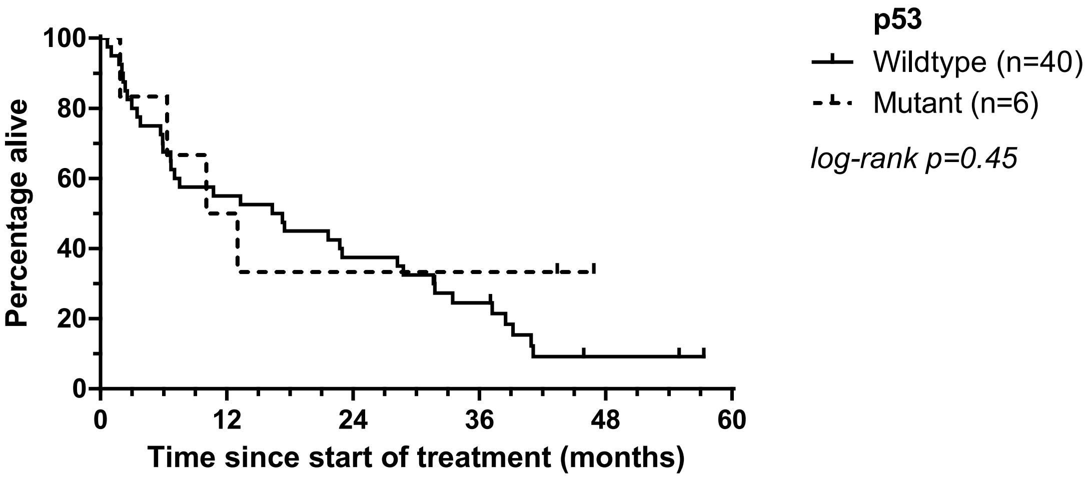

© 2025 Kristeleit R et al. *JAMA Oncology*.

(D)

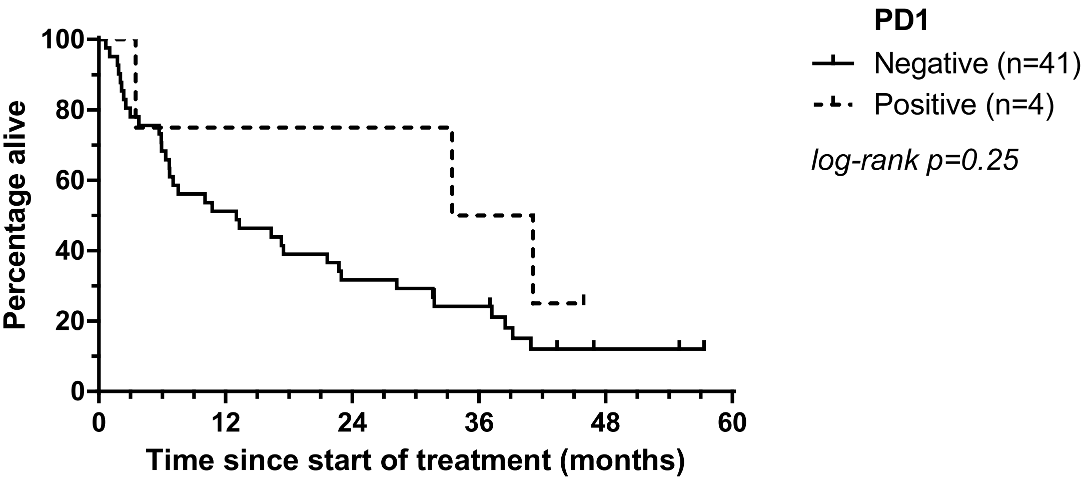

(E)

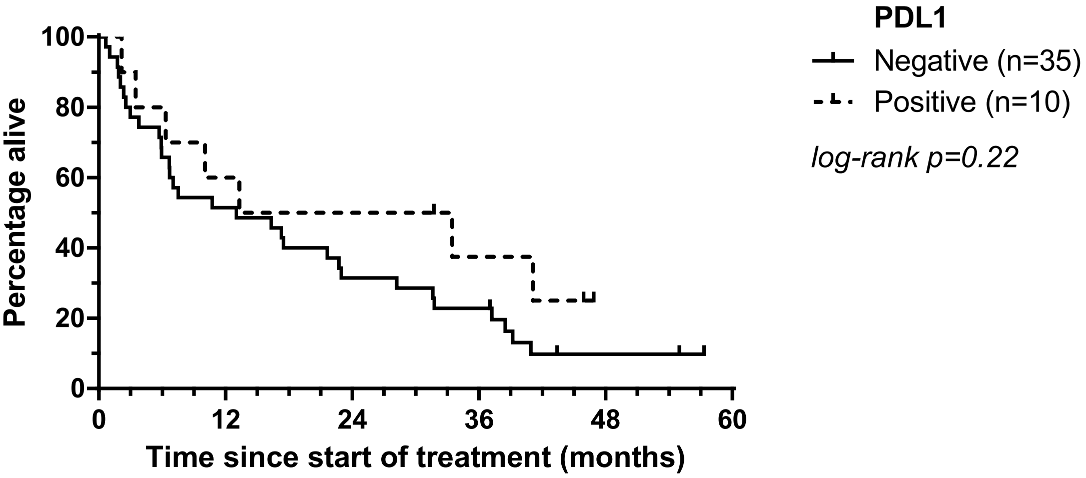

(F)

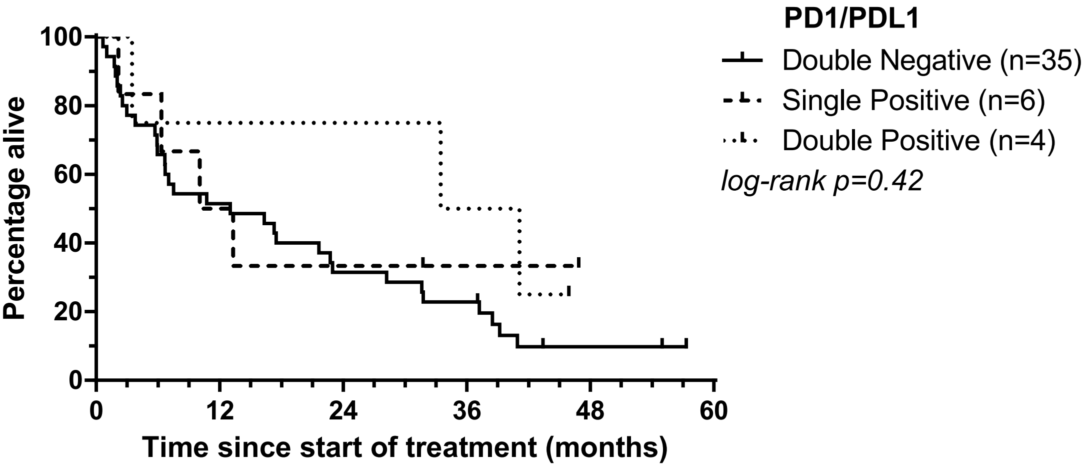

© 2025 Kristeleit R et al. *JAMA Oncology*.
