# Supplementary material for: Pembrolizumab in Patients With Advanced Clear Cell Gynecological Cancer: A Phase 2 Nonrandomized Clinical Trial
Source: JAMA Oncol. 2025 Feb 6;11(4):377–85. doi: 10.1001/jamaoncol.2024.6797 (PMC11803509; doi:10.1001/jamaoncol.2024.6797)
Supplement: Supplement 3. — Data Sharing Statement [file jamaoncol-e246797-s003.pdf]

Data Sharing Statement

Kristeleit. Pembrolizumab in Patients With Advanced Clear Cell Gynecological Cancer. *JAMA Oncol*. Published February 06, 2025. doi:10.1001/jamaoncol.2024.6797

Data
Additional Information: Clinicaltrials.gov
https://classic.clinicaltrials.gov/ct2/show/NCT03425565 NCT03425565
Data available: Yes
Data types: Deidentified participant data
How to access data: Requests for data can be made in writing to the Director of the CR UK & UCL Cancer Trials Centre, Professor Allan Hackshaw, a.hackshaw@ucl.ac.uk. Data sharing will be subject to Sponsor (UCL) review: https://www.ctc.ucl.ac.uk/DataSampleSharing.aspx
When available: With publication

Supporting Documents
Document types: None

Additional Information
Who can access the data: Researchers whose proposed use of the data has been approved.
Types of analyses: Specified purposes.
Mechanisms of data availability: With a signed data access agreement.
